# Automated Adaptive Absolute Binding Free Energy Calculations

**DOI:** 10.1021/acs.jctc.4c00806

**Published:** 2024-09-10

**Authors:** Finlay Clark, Graeme R. Robb, Daniel J. Cole, Julien Michel

**Affiliations:** † EaStCHEM School of Chemistry, University of Edinburgh, David Brewster Road, Edinburgh EH9 3FJ, United Kingdom; ‡ Oncology R&D, AstraZeneca, Cambridge CB4 0WG, United Kingdom; § School of Natural and Environmental Sciences, Newcastle University, Newcastle upon Tyne NE1 7RU, United Kingdom

## Abstract

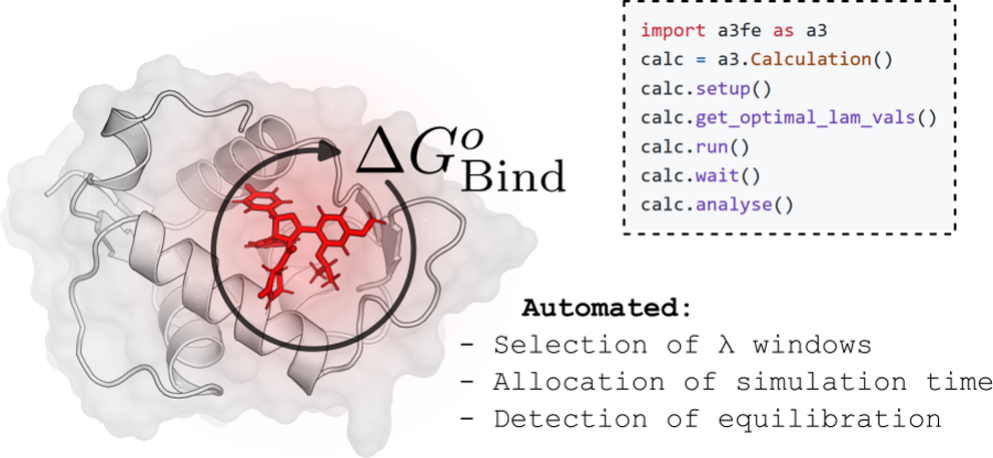

Alchemical absolute binding free energy (ABFE) calculations have substantial potential in drug discovery, but are often prohibitively computationally expensive. To unlock their potential, efficient automated ABFE workflows are required to reduce both computational cost and human intervention. We present a fully automated ABFE workflow based on the automated selection of λ windows, the ensemble-based detection of equilibration, and the adaptive allocation of sampling time based on inter-replicate statistics. We find that the automated selection of intermediate states with consistent overlap is rapid, robust, and simple to implement. Robust detection of equilibration is achieved with a paired *t*-test between the free energy estimates at initial and final portions of a an ensemble of runs. We determine reasonable default parameters for all algorithms and show that the full workflow produces equivalent results to a nonadaptive scheme over a variety of test systems, while often accelerating equilibration. Our complete workflow is implemented in the open-source package A3FE (https://github.com/michellab/a3fe).

## 1 Introduction

The binding affinity of a drug to its target is an important quantity in early stage drug discovery. Its quick and accurate prediction would allow the wasteful synthesis of weakly binding molecules to be avoided. Substantial progress has been made toward this goal, with rigorous “alchemical” free energy calculations based on molecular dynamics or Monte Carlo sampling recommended as the optimal method.^1−3^ Alchemical relative binding free energy (RBFE) calculations, which evaluate the relative difference in binding affinity between structurally related molecules, are now routinely applied in the hit-to-lead and lead optimization stages of drug discovery.^4^ However, standard RBFE calculations are limited to structurally similar molecules binding to the same target with the same binding mode.^5^

Alchemical absolute binding free energy (ABFE) calculations escape these constraints through their more general formulation.^6,7^ RBFE calculations are based on a thermodynamic cycle where two ligands are interconverted in solution and while bound to their target; ABFE calculations involve removing a single ligand’s intermolecular interactions in both environments.^8^ This makes ABFE calculations applicable to a wider class of problems,^9,10^ including accurately ranking diverse molecules in the final stages of high-throughput virtual screening.^11,12^

Unfortunately, the application of ABFE calculations is limited by their high computational cost. ABFE calculations require a relatively large modification of the system (complete removal of ligand intermolecular interactions) which increases the thermodynamic length around an ABFE cycle compared to an RBFE cycle.^13,14^ This necessitates more sampling for a result of equal precision. ABFE calculations are also particularly susceptible to sampling issues such as slow rehydration of the binding site and side-chain rearrangement.^15^ Calculating the relative binding free energy between a pair of similar ligands using a default ABFE protocol would be around an order of magnitude more expensive than with a default RBFE protocol,^12,16^ and would likely be less precise.

Clearly, ABFE calculations must become more efficient to realize their potential. A promising class of methods yield relative binding free energies between structurally dissimilar ligands while aiming to avoid the sampling issues associated with “emptying” the binding site.^17−21^ Approaches combining active learning of affinity predictions with free energy calculations have demonstrated substantial efficiency improvements by reducing the number of simulations required to identify potent binders.^22−26^ Nonequilibrium calculations involving “swarms” of short switching trajectories may decrease wall-clock time in some cases.^15,27−29^ A plethora of enhanced sampling techniques have been applied to alchemical free energy calculations, sometimes producing dramatic improvements in accuracy and efficiency.^30−45^ However, many of these techniques require system-specific tuning, yield highly system-specific benefits,^15^ may require frequent communication between otherwise independent replicas, and may even degrade performance.^46^

There are also areas in the standard ABFE cycle where substantial human and computer time is often wasted through manual setup or poor defaults, and where automation and adaptive algorithms may offer substantial efficiency improvements.^47−50^ This avenue for efficiency improvement is much less well-explored than the approaches mentioned above and may be particularly beneficial for ABFE calculations, which vary widely in terms of sampling requirements and equilibration and convergence behavior. Here, we investigate three of these areas.

The first area is the spacing of intermediate states. It is usually impossible to obtain an accurate estimate of a free energy difference with alchemical methods by sampling only at the end-states of interest. For overlap-based free energy estimators such as the Bennett Acceptance Ratio (BAR) or the Zwanzig equation,^51^ this is because sampling at one end-state almost never produces samples with non-negligible probability at the opposite end state. For thermodynamic integration (TI), errors are introduced because the potential of mean force (PMF) with respect to an alchemical variable (which smoothly interpolates the end state Hamiltonians) is poorly approximated. The problem is solved in both cases by sampling at intermediate states. The variable controlling the interpolation of intermediate states is usually called λ, and intermediate states may be referred to as λ windows.

Optimal intermediate states would minimize the standard error of the overall free energy estimate for a given total sampling time. However, selecting these would require knowledge of the statistical inefficiencies and divergences of the probability distributions along all possible paths between end states.^14,52^ It should be noted that the optimal path is influenced by the relative efficiencies of sampling at intermediate states, and is therefore dependent on the sampling methodology as well as the Hamiltonians. It appears challenging to obtain this information without costly simulations which would reduce overall efficiency. Nevertheless, a number of approximate approaches have been developed.

Blondel^53^ and later Pham and Shirts,^54^ derived schemes to select minimum-variance pathways between end-state Hamiltonians. Their derivations assumed infinitely close spacing of intermediate states along the pathway and ignored statistical inefficiencies, which reduced the optimized schedules’ efficiencies. Reinhardt and Grubmüller also ignored correlation times but accounted for the finite spacing of intermediate states.^55^ There have been attempts to reduce correlation times by smoothing energy barriers,^41,56^ and by constraining the selected path to avoid problematic regions of parameter space.^57,58^

A particularly simple approach is to optimize only the number and spacing of states along a predetermined path. We limit ourselves to this approach here. Most approaches to this problem involve short initial simulations, although König et al. developed a simple metric to estimate the required number of intermediate states based on the energy difference between minimized end-state structures.^59^ Several methods have been proposed that space intermediate states by equal thermodynamic distances. This has been shown to minimize total variance of the free energy estimate for any unbiased estimator, assuming infinitely close spacing of states.^60^ Methods which do,^61,62^ and do not,^63,64^ account for the autocorrelation of samples have been suggested. Methods based on heuristics have also been proposed.^65,66^ Recently, Zhang et al. built models to predict nonlocal phase-space overlap, which were used to select λ-schedules with equal phase space overlap between adjacent windows.^67^ However, the use of any protocol for intermediate state selection is still rare in alchemical free energy calculations, and extensive investigation of optimal selection protocols and potential benefits is lacking.

The second area is the allocation of sampling time. For a free energy calculation using information from two states, Bennett noted that the equal allocation of sampling time between states is at worst half-optimal.^51^ This is because the allocation of 1 h of sampling to each state (2 h total sampling) cannot yield a worse estimate than the optimal allocation of a total of 1 h of sampling between both states, given that the variance of the estimate decreases monotonically with the number of samples from each state. However, an absolute binding free energy calculation uses samples from many (*N*) states. The lower bound on the estimation efficiency with equal sampling time then falls to 1/*N* of the optimal allocation. Therefore large efficiency improvements may be made by the optimal allocation of sampling time when few states have a much higher computational cost per independent sample.

Sun et al. adaptively allocated simulation time during absolute hydration free energy calculations and an RBFE calculation.^68^ Time allocation was based on the time derivatives of the variance of the free energy estimates between adjacent λ windows obtained with the Bennett Acceptance Ratio for a single run.^51^ However, this did not substantially improve the efficiency of the RBFE calculation due to relatively short and similar autocorrelation times of the perturbed energy differences between windows. A similar approach is more likely to benefit ABFE calculations, which are more susceptible to dramatic variations in correlation time between windows as a result of sampling issues. Indeed, Mendoza-Martinez et al. demonstrated that the equilibration of ABFE calculations could be accelerated by allocating sampling time based on the uncertainties of the interwindow free energy changes.^69^ In contrast to Sun et al., errors were calculated from the difference between replicate runs, which is a robust method for uncertainty quantification.^70−75^ However, this protocol lacks a rigorous derivation, and no automated implementation is available. While this work focuses on the adaptive allocation of sampling time within single ABFE calculations, Li et al. showed how adaptively allocating simulation time to individual calculations within a network could substantially improve statistical precision.^50^

The final area is the detection of equilibration and convergence. Intermediate state simulations in free energy calculations are usually started from coordinates sampled with the ligand fully interacting. These coordinates may have a very low probability under the equilibrium distributions of other states, producing initial transients in the estimated free energy changes as the system relaxes. It is standard practice to discard samples from these equilibration (burn-in) periods to reduce bias in the final free energy estimate.^5^ Convergence refers to the approach of the free energy estimate to its asymptotic (infinite sampling) value. Methods to assess equilibration and convergence are essential to increase confidence that finite-time simulations are representative of the underlying stationary distributions. However, it is never possible to state this with certainty.^76^

In this work, free energy estimates are termed “unequilibrated” if they show clear bias compared to results from longer simulations, or if they change significantly with additional sampling time at late times. Otherwise, they are termed “equilibrated”. This definition is consistent with its use in the field, but does not guarantee the removal of bias compared to the true infinite sampling value. We refer to estimates as “unconverged” if there is evidence that they are unstable or inaccurate compared to their infinite sampling values. Hence, “unequilibrated” estimates are always “unconverged”, and “equilibrated” estimates may still be “unconverged”. This contrasts with frequent practice in the literature, where “equilibrated” estimates are always described as “converged”.

In free energy calculations, the most well-known equilibration detection protocols are those of Chodera and Yang et al.^77,78^ Yang et al. monitored the behavior of the reverse cumulative average of the free energy gradient. Contamination by nonequilibrated data was detected by deviation from normality. Chodera proposed a nonparametric method which selects the unequilibrated region by maximizing the effective sample size of the production region. Both methods are based on the data from a single run. As a result, both are susceptible to erroneously discarding the majority of simulation data when the system becomes trapped in a local minimum. Convergence is typically assessed using uncertainties obtained from a single run (after block-averaging or subsampling to remove correlation) or, more rigorously, from differences between repeated runs.^71^

Despite similarity of the underlying problem, different diagnostics are used in the Markov Chain Monte Carlo (MCMC) literature.^79^ The most common method for assessing sampling seems to be the Gelman–Rubin diagnostic,^80^ which effectively compares the variances estimated within and between MCMC chains. If individual chains (simulations) are initially overdispersed and become trapped in local minima, the interchain variances will be greater than the intrachain variances. This manifests as a Gelman–Rubin *R̂* > 1, indicating that individual chains have failed to converge to the same stationary distribution. MCMC equilibration detection methods include the Geweke test,^81^ which checks for a significant difference between the means of the initial and final portions of the data. These diagnostics have occasionally been used in molecular simulation (for example by García and Hasse) but,^82^ to our knowledge, they are yet to be exploited in free energy calculations.

Here, we attempt to improve the efficiency of ABFE calculations by developing and evaluating automated protocols in each of the areas mentioned: the spacing of intermediate states, the allocation of sampling time, and the detection of equilibration. Where possible, we use ensemble-based metrics to increase robustness. We have sought to minimize user time, in addition to computational time, by implementing our protocols in an easy-to-use open-source python package, https://github.com/michellab/a3fe A3FE, which is itself based on BioSimSpace and SOMD.^83,84^

## 2 Theory

### 2.1 The Optimum Allocation of Resources Along a Path

In this work, we assume that our path through alchemical space is already defined (by our choice of soft-core potentials, etc.), and that progress along this path is controlled by a single interpolating variable, λ. We want to determine the optimal allocation of sampling time along this path which minimizes the uncertainty in the final free energy estimate for a constant total sampling time.

It is convenient to start by assuming infinitely close spacing of intermediate states, which makes the uncertainties of estimators such as TI, Zwanzig, and BAR equivalent (see Section S1). Under this assumption, the estimated free energy change between λ = 0 and 1 is,^85^
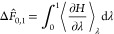
1The Hamitonian, *H*(λ, **x**), is dependent on λ and on the positions and momenta of all particles in the system, **x**, but is written *H* for brevity. The free energy change calculated is only an estimate (denoted by the hat operator) because we cannot fully enumerate the desired ensemble. Instead, ⟨...⟩_λ_ denotes an average obtained from statistical sampling (molecular dynamics) at λ and taken to be equivalent to the ensemble average (the ergodic hypothesis). The variance of the estimated free energy change is
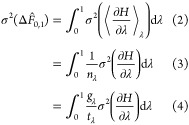
2where *n*_λ_ is the number of uncorrelated samples obtained at λ,  is the variance of the mean gradient of the Hamiltonian with respect to λ,  is the variance of the gradient of the Hamiltonian with respect to λ,  is the statistical inefficiency at λ, *t*_λ_ is the sampling time at λ, and we have assumed independent sampling at different values of λ. Defining the fractional sampling time as , where *t*_Tot._ is the total sampling time for the transformation, yields,
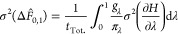
5The total fractional sampling time must equal 1. σ^2^(Δ*F̂*_0,1_) can be minimized subject to this constraint by creating the Lagrangian function
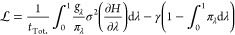
6and setting its derivative with respect to π_λ_ to 0 for all λ
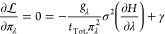
7
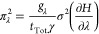
8
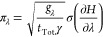
9The integrals over λ have disappeared because this condition must be true for all λ. This approach is similar to that of Sun et al. but is based on TI rather than BAR.^68^Equation 8 rearranges to , where  is the derivative of the variance of the estimated free energy change (eq 4) with respect to *t*_λ_ at a fixed value of λ. This means that the time derivative of the variance of the mean is a constant for all λ when the allocation of sampling time is optimum, as discussed by Sun et al.^68^ The intuition is that if there are states with higher time derivatives of the variance, then sampling time would have been better allocated to these than states where the additional sampling time reduced uncertainty less.

The Lagrange multiplier, γ, can be determined by substituting eq 9 into the condition ∫_0_^1^π_λ_dλ = 1:
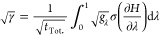
10Hence the choice of fractional sampling time allocation which minimizes the overall uncertainty is
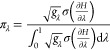
11Substituting this into eq 5 to obtain the minimum variance of the free energy change estimates provides a limit for the minimum variance of the overall free energy change,
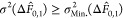
12
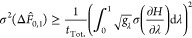
13
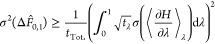
14The expected fractional reduction in simulation time required for equivalent uncertainty with optimal spacing can be quantified with
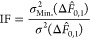
15which is equivalent to the improvement factor (IF) of Lundborg et al. when σ^2^(Δ*F̂*_0,1_) is obtained with a uniform distribution of sampling time.^62^

We could have presented an equivalent discussion in terms of thermodynamic length.^13,14,52,60,62^, or equivalently , can be regarded as a measure of thermodynamic length which accounts for autocorrelation. If autocorrelation is ignored, *g* = 1 for all values of λ and the appropriate measure of thermodynamic length is . However, the assumption of *g* = 1 means that minimizing this length does not minimize overall uncertainty.

In equilibrium free energy calculations, simulations are usually carried out at discrete values of λ. Two methods to modify π to minimize σ^2^(Δ*F̂*_0,1_) are changing the density of λ widows, and changing the allocation of sampling time to each window.

#### 2.1.1 Changing the Density of Windows

**Figure 1:**
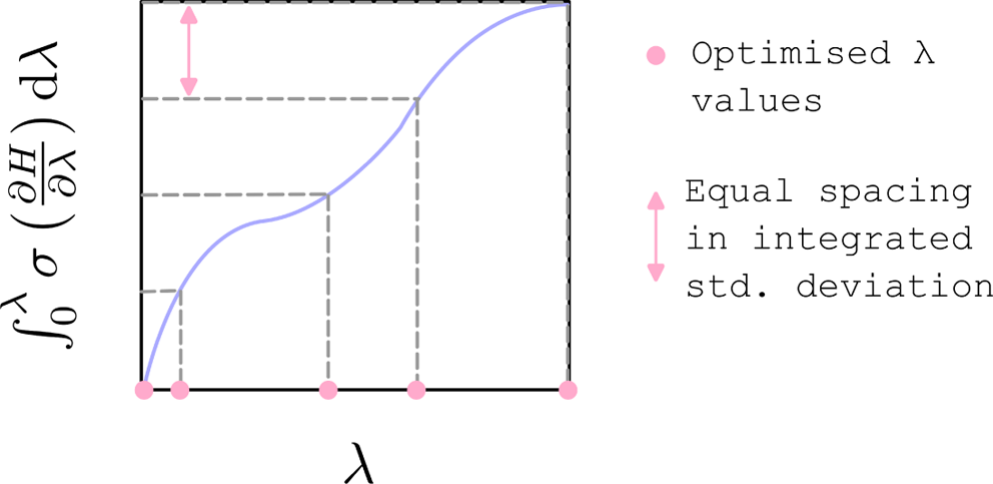
An illustration of the λ-spacing algorithm, where  is used. The variation of  with λ is estimated from short initial simulations with a fixed default λ schedule and used to estimate  as a function of λ. The selected “thermodynamic speed” determines the constant step size in , which is used to select the optimized λ-schedule. Regions where  increases quickly as a function of λ suggest quickly changing probability distributions in configuration space, which produce more densely spaced λ windows.

π can be increased around a given value of λ by increasing the density of λ windows. For a transformation with *K* windows where there is an equal allocation of sampling time to each window, each of the *K* – 1 Δ*λ* “steps” is effectively allocated a fraction of  of the total simulation time. The approximate (due to the earlier assumption of infinitely close states) optimum spacing between windows *k* and *k* + 1, Δ*λ*_*k*,*k*+1_, can be found from eq 11:
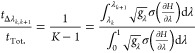
16
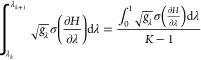
17
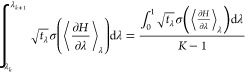
18Alternatively, assuming *g* = 1 for all λ,
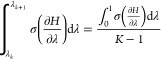
19Hence, to determine the optimal spacing, we need an estimate of  or  as a function of λ. One way to obtain this is from an initial short set of simulations with nonoptimal spacing. The windows can then be spaced according to eqs 19 or 18 by ensuring that the areas under the  or  curves between each value of λ are equal. We can either specify *N*_Wind._, or the entire right-hand sides of eqs 19 or 18. We term these “thermodynamic speeds” following Minh, who discussed the spacing of windows according to eq 19.^63^ We specify a constant thermodynamic speed, ensuring that equivalent spacing is obtained between different transformations of varying thermodynamic length. This algorithm is illustrated in Figure 1 for the case where  is used.

A common approach to spacing λ windows is to aim for consistent and sufficient overlap between adjacent windows.^86^ The uncertainty of the BAR estimator can be expressed in terms of overlap,^51^ but also in terms of the variance of the gradient (under our assumption of infinitely close window spacing, see Section S1). Hence, these spacing approaches are closely related and the variance of the gradient must predict the overlap in the limit of infinitely closely spaced windows. Intuitively, this is because the dimensionless variance of the gradient is the average squared relative change in probability with λ
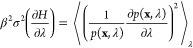
20where *p*(**x**, λ) is the probability of observing the phase-space coordinates **x** at λ and  (Section S2).

#### 2.1.2 Changing the Allocation of Sampling Time

Varying window spacing with a constant time allocation per window is one strategy to achieve optimal sampling times at each λ, minimizing overall uncertainty. A complementary strategy is to allocate different sampling times to each window.

For *K* windows, we can approximate eq 5 as
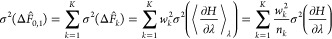
21where there are *K* states with state number *k*, *w*_*k*_ is the state weight given by the numerical integration method, and Δ*F*_*k*_ is the contribution of a single state to the overall free energy change, such that Δ*F*_0,1_ = ∑_*k* = 1_^*K*^Δ*F*_*k*_.

From eq 11, we can write
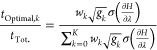
22where *t*_Optimal,*k*_ is the estimate of the optimal sampling time at state *k*. The data currently available from state *k* can be used to effectively estimate the statistical inefficiency according to , where *t*_Current,*k*_ is the current sampling time at state *k* and *n*_Current,*k*_ is the current number of uncorrelated samples. Additionally, the denominator of eq 11 can be written in terms of the minimum achievable variance of the free energy change estimate (eq 14)
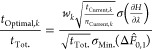
23
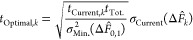
24eq 24 shows how to estimate the optimal sampling time at state *k* from the current state sampling time and standard error of the state free energy change contribution, σ_Current_(Δ*F̂*_*k*_). However, we avoid allocating sampling time with a specified *t*_Tot._ for each calculation because this reduces the efficiency over multiple calculations compared to specifying a shared “runtime constant”, 
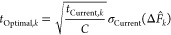
25This allows the total simulation time per calculation to vary to reduce the overall uncertainty for the set of calculations. Repeated cycles of simulation and re-estimation of the optimum sampling time with eq 25 are performed until the estimated optimum sampling time is equal to, or less than, the current sampling time.

Sun et al. derived an adaptive time allocation protocol based on the intrarun BAR errors.^68^ Our approach differs in that we calculate the uncertainties (σ_Current_(Δ*F̂*_*k*_)) from the differences between independently equilibrated replicate runs, rather than from intrarun fluctuations. To allow easy decomposition of the uncertainties into contributions from single states, increasing independence, we base our algorithm on the errors from TI rather than BAR. Theoretically, each set of repeat simulations at a given state can be run independently. During the preparation of this work, Yu et al. proposed a similar algorithm.^87^

### 2.2 Detection of Equilibration

Our equilibration detection heuristic takes inspiration from the Gewke test,^81^ which checks the equality of means of initial and final portions of the data. Typically, the first 10% and last 50% of the data are used. Significantly different means are taken to indicate a systematic drift and therefore lack of equilibration.

We aim to detect systematic drift of the free energy estimate from an ensemble of independent replicates while ignoring systematic differences between the replicate estimates. Therefore, we perform a paired *t*-test between the free energy estimates from the first 10% and final 50% of the data for each replicate. This is repeated while sequentially discarding more data from the start of the simulation up to some threshold. The data is accepted as equilibrated when there is no evidence for a significant difference between the paired samples at the 95% confidence level.

## 3 Methods

### 3.1 System Preparation

In general, the initial test systems were prepared from crystal structures, retaining crystal waters and discarding all other nonprotein and nonligand atoms. Details of initial structure preparation for individual systems are given in Section S3 and the rationale for system selection is given in Section 4.1. Alternate atom locations with the greatest occupancy were selected (or the A state, if occupancies were equal). Missing atoms were added using pdb4amber.^88^ The protonation states of all residues were assigned using H++ (version 3.2).^89^ Proteins and crystal waters were parametrized with the AMBER ff14SB and TIP3P force fields using antechamber 22.0.^90−92^ Ligands were protonated using Open Babel (version 3.0.0) and parametrized with OpenForceField 2.0.0 and AM1-BCC partial charges through BioSimSpace (version 2023.0.0).^83,93−96^ The protein–ligand complexes and free ligands were solvated with TIP3P water and 0.15 M of NaCl in rhombic dodecahedral boxes, ensuring a minimum distance of 15 Å between the solute and the box edge. The Cyclophilin D systems were taken from the “abfe_2fs” input supplied by Alibay et al. (GAFF2 with AM1-BCC partial charges, ff99SB-ILDN, and TIP3P force fields).^97,98^ These were used with two minor modifications—the ligands were extracted and reparameterized with GAFF2.11 and AM1-BCC partial charges using antechamber 22.0 to avoid issues with noninteger charges, and the boxes were expanded to rhombic dodecahedral boxes with a minimum solute-box edge distance of 15 Å to allow the use of a 12 Å cutoff for reaction-field electrostatics.

### 3.2 Molecular Dynamics Protocols

All solvated systems (both “initial” and obtained from Alibay et al.) were subject to the standard A3FE minimization and equilibration workflow, in which all simulations were performed using GROMACS 2021.3 through BioSimSpace (version 2023.0.0).^99^ Systems were energy minimized for 1000 steps, followed by equilibration in the *NVT* ensemble (5 ps with all nonsolvent atoms restrained and heating from 0 to 298 K, followed by 50 ps with restraints on all backbone atoms for the complexes only, then 50 ps with no restraints). *NPT* equilibration was then performed at 1 atm and 298 K (400 ps with restraints on nonsolvent heavy atoms, followed by 1 ns with no restraints). Finally, independent 5 ns *NPT* equilibration runs were carried out for each independent replicate run for all systems to provide varied starting conformations. All restraints used a force constant of 10 kcal mol^–1^ Å^–2^. The V-rescale and C-rescale algorithms were used for the relevant equilibration steps, with time constants of 1 and 4 ps, respectively.^100,101^ All equilibration molecular dynamics simulations used a time step of 2 fs and a cutoff of 8 Å for short-range interactions, with electrostatic interactions computed with the PME algorithm.^102^

### 3.3 Absolute Binding Free Energy Calculations

#### 3.3.1 Individual Calculations

ABFE calculations were carried out using the double decoupling method of Gilson et al.,^6^ according to the thermodynamic cycle shown in Figure 2 of Clark et al.^103^ The simulations with the ligand in solvent collectively make up the free leg, while those with the receptor–ligand complex make up the bound leg. Sets of calculations where interactions of a given type are introduced or removed are termed stages: receptor–ligand restraints were introduced, charges were scaled, and Lennard-Jones (LJ) terms were scaled in the restrain, discharge, and vanish stages, respectively. Standard free energies of binding were calculated according to
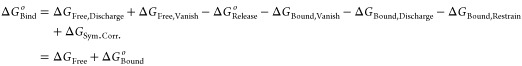
26where Δ*G*_Sym. Corr._ includes any required symmetry corrections and Δ*G*_Release_^*o*^ is the free energy of releasing the noninteracting ligand to the standard state volume.^104^ Δ*G*_Free_ and Δ*G*_Bound_^*o*^ are the overall free and bound leg contributions to Δ*G*_Bind_^*o*^, where Δ*G*_Bound_^*o*^ includes all terms other than Δ*G*_Free,Discharge_ and Δ*G*_Free,Vanish_. The relative receptor–ligand external degrees of freedom were restrained using Boresch restraints and Δ*G*_Release_^*o*^ was calculated using an analytical correction term.^7,105^ The coupling parameter, λ, was scaled from 0 to 1 to gradually introduce Boresch restraints, and to remove charges and LJ terms in the relevant stages. λ linearly scaled the magnitude of restraint force constants and charges, while a soft core potential based on that of Zacharias et al. was used to scale LJ terms (soft-core parameter set to 2.0).^106^

**Figure 2:**
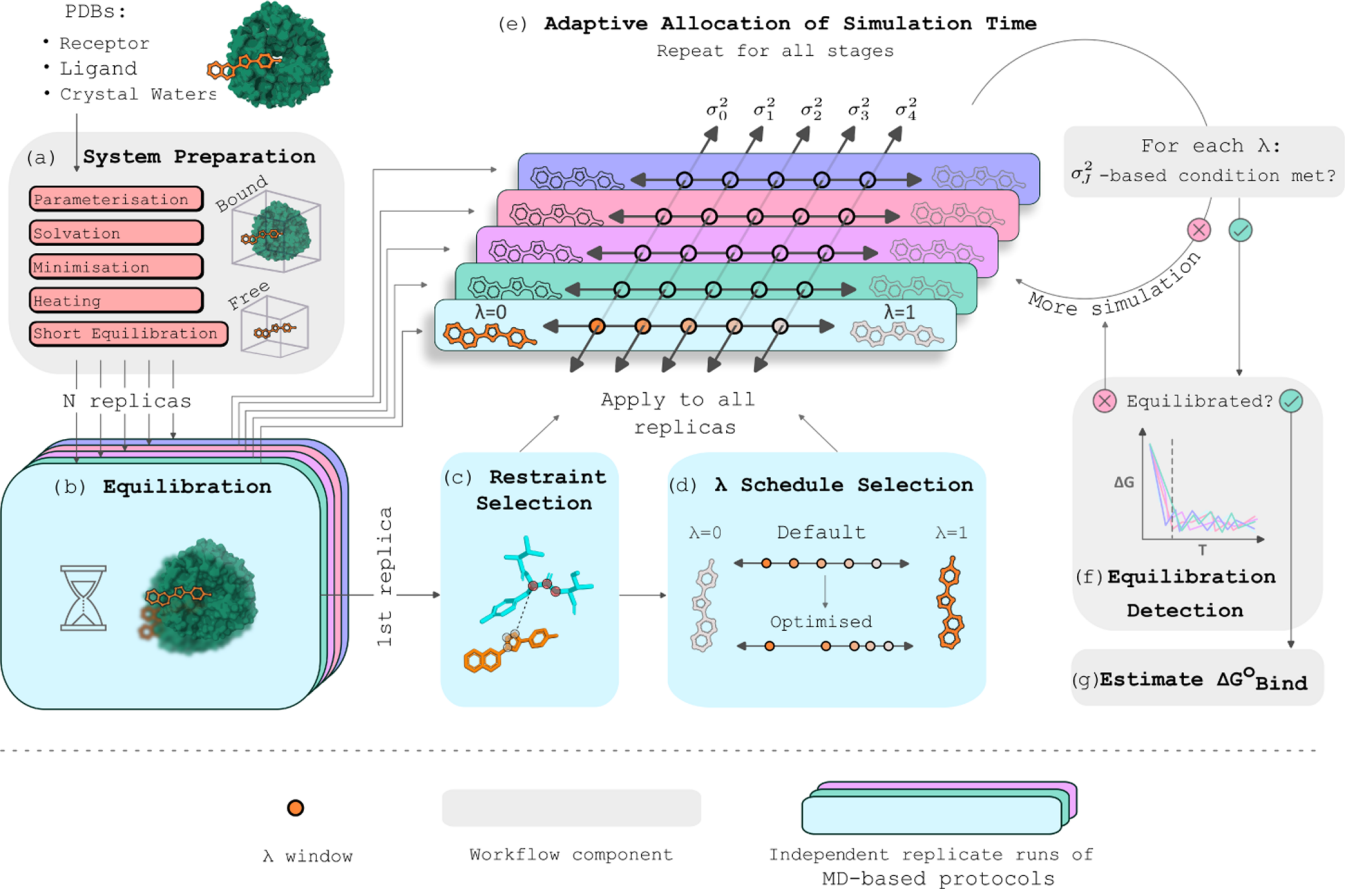
Scheme of the automated absolute binding free energy workflow implemented in the A3FE python package. Following (a) initial system preparation, (b) individual 5 ns equilibration runs were carried out for each of the *N* replicate runs for each leg to provide diverse input conformations for the free energy calculations. (c) The first of these for the bound leg was analyzed to select Boresch restraints. (d) A single short replicate was run for each of the default λ values to provide an estimate of the variance of the gradient, from which the final λ values were calculated. The same Boresch restraints and λ scheme were used for all replicate production runs. (e) Simulation time was allocated by iteratively calculating the inter-run standard errors of the per-window Δ*G* estimates, estimating the optimal run time for each window, and allocating sampling time accordingly. Once the estimated optimal sampling times were achieved, (f) the equilibration time was determined for each stage using an ensemble-based equilibration detection heuristic, (g) and the overall free energy changes were calculated using MBAR.

Individual simulations were carried out using Sire/OpenMM Molecular Dynamics (SOMD),^107,108^ which is available within Sire (version 2023.1.3).^84^ OpenMM’s LangevinMiddleIntegrator was used (friction coefficient of 1 ps^–1^, coupled to a heat bath at 298 K).^109^ Pressure was maintained at 1 atm using a Monte Carlo barostat (isotropic box scaling attempts every 25 time steps). Hydrogen mass repartitioning (with a repartitioning factor of 3) was used to allow a time step of 4 fs, as done in earlier ABFE studies with this simulation engine.^110,111^ Bonds to hydrogen were constrained. A cutoff of 12 Å was used for all nonbonded interactions and electrostatics were treated with the reaction field method with a dielectric constant of 78.3.^112^ We did not correct for the truncation of the tails of the LJ potentials because we found this produced negligible corrections. Energy minimization with a maximum of 1000 iterations was performed prior to every simulation. The gradient of the free energy with respect to λ was calculated every 200 timesteps.

#### 3.3.2 Overall Workflow

The complete workflow (Figure 2) was carried out using the python package A3FE (version 0.1.0), which is available on Github at https://github.com/michellab/a3fe and is built on BioSimSpace and SOMD.^83,84^ The final 5 ns equilibration run of the fully interacting complex for the first replicate was analyzed to select the Boresch restraints using BioSimSpace. This algorithm fits the force constants to the fluctuations observed for prospective sets of anchor points, then selects the stable anchor set which maximally restricts the configurational volume available to the fully decoupled ligand. This effectively mimics strong native receptor–ligand interactions and ensures that Δ*G*_Bound,Restrain_ ≈ 1.23 kcal mol^–1^ (see Section S6 in Clark et al. for a discussion and Hedges et al. for a tutorial).^103,113^ The same restraints were used for all replicate runs to ensure that the free energy gradients at a given λ value would converge to the same values with infinite sampling. Different initial configurations were used for each leg of each repeat run, as extracted from the end of the 5 ns equilibration step. Within each leg, the same initial configuration was used for each λ-window.

The λ-schedule from Clark et al.,^103^ which was manually optimized for the MIF/MIF180 system, was used as a default. This used 8 windows for the free discharge stage, 18 for the free vanish stage, 6 for the bound restrain stage, 8 for the bound discharge stage, and 36 for the bound vanish stage. This schedule was used to run all nonadaptive simulations for the initial test systems. For adaptive simulations, the λ-schedule at each stage was generated by running very short simulations (0.1 ns, no replicates) at all default λ values. These were used to estimate  as a function of λ, and the new λ-schedule was generated so that the area under the  vs λ curve was equal between adjacent λ values. The size of these areas, and hence the spacing of the windows, were determined by the user-specified “thermodynamic speed” parameter.^63^

The adaptive runs began with 0.2 ns simulations for all replicates. Per-window free energy changes were calculated from the product of  and weights from the trapezoidal rule. From the inter-run deviations, the standard errors of the mean free energy changes were calculated and used to predict the optimal run times according to eq 25. Additional simulation time was then allocated accordingly, and iterations of sampling time allocation and analysis were performed until predicted optimum sampling times agreed with the allocated sampling times. Equal sampling times were allocated to all replicate windows at the same value of λ. To promote smooth convergence to the “optimal” time, the total sampling time for each window was no more than doubled during each cycle. To smooth the allocation of sampling time between windows, it was enforced that the total sampling time for a given window could be no less than half that of the adjacent windows. Total sampling time was controlled with the user-input runtime constant (*C* in eq 25). To obtain true optimum efficiency, times and statistical inefficiencies should be measured in terms of computing time (or power consumption/ financial cost), rather than in nanoseconds of simulation time. This accounts for the differences in computational costs of different systems. To account for the different computational costs of different legs for different systems, *t*_Current,*k*_ and *t*_Optimal,k_ were multiplied by the relative computational cost of the current calculation leg, resulting in an overall factor of  on the right-hand side of eq 25. Costs were calculated for all systems from the λ selection simulations, which were all run on the same machine with four GeForce RTX 2080 SUPER GPUs. These were expressed relative to the MIF180 bound leg, which had an absolute computational cost of 0.21 h ns^–1^. Hence, eq 25 was used as shown with simulation times for the MIF180 bound legs, but where the computational cost was greater, the predicted optimal time was reduced. The approach of using relative costs was intended to improve the repeatability of the workflow—once users have calculated the absolute cost of the MIF180 bound leg, for which input files are supplied, this can be supplied to A3FE and the resultant simulation times (in ns) should be comparable to those shown here. The effect of varying hardware was removed by expressing computational time in GPU hours on GeForce RTX 2080 SUPER GPUs, obtained by multiplying simulation time in ns by the absolute cost calculated during the λ selection simulations.

Equilibration detection was performed after the adaptive allocation of sampling time was complete. This was done by splitting all time-ordered data for a stage into 100 blocks, so that each contained the same fraction of data (but not necessarily sampling time) from each λ window. The free energy change at each percent of total stage simulation time was computed using the Multistate Bennett Acceptance Ratio (MBAR) as implemented in pymbar 3.1.1.^51,114^ The mean Δ*G* estimates for the first 10% and last 50% of the data were calculated for each run and a paired *t*-test was performed on the per-run differences. This was repeated after discarding 17, 33, and 50% of total simulation time. Equilibration was equated with the first *p*-value greater than 0.05. Unequilibrated data were discarded and Δ*G*_Bind_^*o*^ was calculated with pymbar. The result was expressed as a mean over all *N* replicates and uncertainties were calculated as 95% *t*-based confidence intervals (using *N* – 1 degrees of freedom). Where not provided, uncertainties in experimental affinity data were assumed to be 0.5 kcal mol^–1^.^115,116^

## 4 Results and Discussion

### 4.1 The Behavior of Non-Adaptive Runs

**Figure 3:**
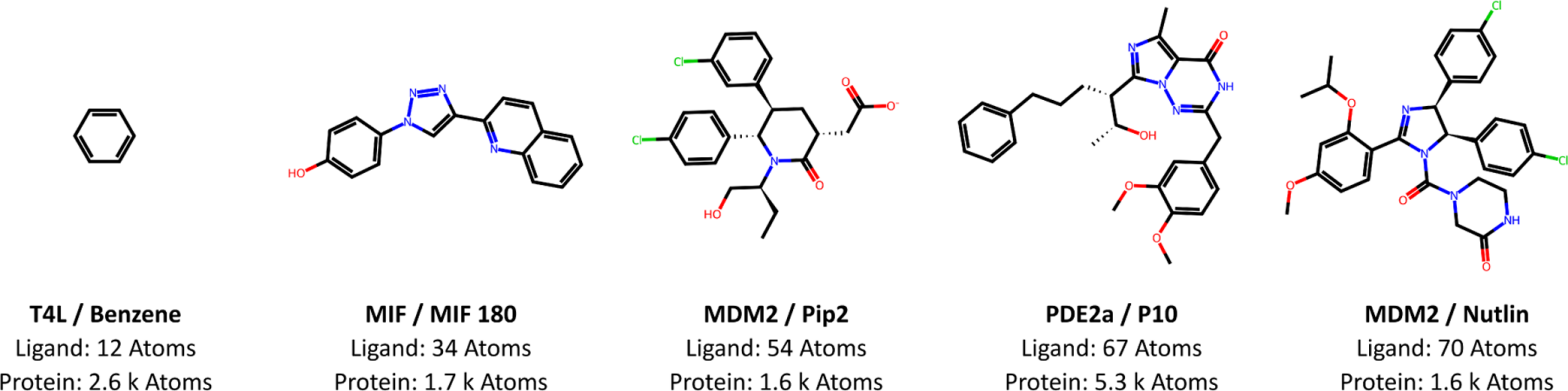
Summary of initial test complexes. Drawn with RDKit.^121^

To inform the development of our adaptive protocols, we first performed nonadaptive ABFE calculations on five protein–ligand systems (Figure 3). These were intended to span a range of ligand sizes, rates of equilibration, and sampling challenges. T4L/Benzene is a common test system for ABFE methodologies;^7,117^ MIF/MIF180 is subject to slow rehydration of the binding site upon ligand decoupling;^103,118^ the MDM2 complexes were used to develop the group’s previous adaptive protocol;^69^ and the slow equilibration of the PDE2a complex was highlighted by Huggins.^119^ T4L, MIF, MDM2, and PDE2a denote the L99A mutant of T4 lysozyme, human macrophage migration inhibition factor, mouse double minute 2 homologue with a truncated lid, and phosphodiesterase 2a, respectively (Section S3). We ran 5 independent replicate runs for each system, all using the manually optimized λ-schedule from our previous work.^103^ First, we investigated short time scale behavior by running all windows for a total of 0.2 ns and using all data for analysis. We then carried out a more realistic protocol optimized for MIF/MIF180 in our previous work—all windows were run for 6 ns with the initial 1 ns discarded to equilibration, other than the bound vanish stages, which were run for 8 ns with the initial 3 ns discarded to equilibration. Finally, we investigated longer time scale behavior by running all windows for 30 ns and discarding the first 10 ns of data to equilibration. The protocols are referred to as 0.2, 6, and 30 ns. As we aimed to compare between protocols rather than with experiment, we tolerated two potential sources of error which we may not have otherwise; the *syn*-*anti* isomerization in MIF180 was rarely sampled, in contrast to our previous work using GAFF2.11,^103,118^ and we performed calculations for the charged ligand Pip2 using a reaction field treatment of electrostatics without maintaining neutrality of the box or applying corrections.^120^

#### 4.1.1 None of the Overall Free Energy Changes are Strictly Converged

Our main interest was in the equilibration and convergence behavior of the nonadaptive runs, and their relative performance compared to adaptive protocols. However, we include experimental binding free energies in Table 1 to check that our results are reasonable. The 30 ns computed free energies are broadly similar to the experimental values, giving some assurance that our default protocol is reasonable. We note that the calculated values appear excessively negative for ligands larger than benzene, in particular the MDM2 ligands. This is to be anticipated for a wide variety of sampling challenges in equilibrium ABFE calculations, because the bound leg simulations are started from the holo structure. Any failure to relax toward the apo structure and free ligand conformations during the simulation can be regarded as erroneous “preorganization” of the system for binding, producing erroneously favorable estimates of binding. This effect can generally be expected to increase for larger ligands with more substantial sampling challenges and more favorable binding affinities, and may be avoided using bidirectional nonequilibrium switching protocols which account for the apo state.^28^ While we make broad comparisons with experimental affinities here, we note that these are from different sources, including biochemical assays.

**Table 1:** Non-Adaptive Δ*G*_Bind_^*o*^ for Initial Test Systems^a^

	0.2 ns	6 ns	30 ns	exp. Δ*G*_Bind_^*o*^	exp. source
T4L	–4.74 ± 0.79	–3.80 ± 0.87	–4.03 ± 0.80	–5.19 ± 0.16	ITC, Morton et al.^122^
MIF	–16.56 ± 2.49	–10.16 ± 1.27	–10.48 ± 1.14	–8.98 ± 0.28	FPA, Cisneros et al.^123^
MDM2-Pip2	–17.32 ± 1.68	–13.49 ± 1.27	–13.94 ± 0.86	–9.11 ± 0.01	ITC, Michelsen et al.^124^
PDE2a	–29.40 ± 2.89	–17.26 ± 1.49	–16.96 ± 1.97	–14.35 ± 0.50*	SPA, Obach et al.^125^
MDM2-Nutlin	–17.88 ± 4.07	–15.56 ± 2.25	–16.92 ± 1.98	–11.14 ± 0.27	ITC, Mendoza-Martinez et al.^69^

a All quantities in kcal mol^–1^. Calculation uncertainties stated as 95% confidence intervals based on the variance of 5 replicate runs, assuming Gaussian distributions, while experimental uncertainties are given as standard deviations. Symmetry corrections are included. A detailed breakdown of the components of the calculated binding free energies is given in Table S1 and all Boresch restraint parameters are given in Table S2. * Uncertainty estimated based on Hahn et al.^115,116^ ITC, FPA, and SPA denote isothermal titration calorimetry, fluorescence polarization assay, and scintillation proximity assay.

More pertinently, the initial test systems show a range of equilibration behaviors. While the results obtained for the brief 0.2 ns runs are not significantly different to those from the costly 30 ns runs for T4L, there are significant and substantial differences for MDM2-Pip2, MIF, and especially PDE2a. As shown by the breakdown of binding energy components (Table S4) and Figure S1, the dominant contribution to slow equilibration comes from the bound vanish legs, where sampling is impeded by the protein and where a second-order phase-change like transition occurs as the Van der Waals interactions are removed.^126^ The diversity of equilibration behavior suggests that equal-time equilibration rules are likely to be suboptimal. However, all cases of slow equilibration are similar in that the overall free energies become less favorable with increasing sampling time, which is to be expected given the discussion of sampling issues above.

The uncertainties in Table 1 do not generally decrease proportionally to the inverse square root of sampling time. This suggests that the increased sampling time does not produce a proportional number of independent samples; rather independent runs are becoming trapped in separate regions of conformational space.^73,127^ Again, Table S4 shows that sampling in the bound leg, and in particular the bound vanish leg is to blame. In contrast to the bound leg, the uncertainties for the free leg generally decrease with sampling time.

While the 6 and 30 ns runs appeared “equilibrated”, we performed further checks to assess convergence. For strictly converged simulations, we would expect replicate runs to sample the same distributions of our parameter of interest. We tested whether the gradients obtained for independent runs were sampled from the same distributions using the Kruskal–Wallis H-test (after subsampling according to the statistical inefficiency determined individually for each run).^77^Figure S2 shows that the proportion of windows showing significant differences at the 95% confidence level is always above 70% for the bound vanish and bound discharge stages. However, for the free legs, the fraction increases with ligand size from around 0 for T4L to similar to the bound fractions for MDM2-Nutlin. This indicates increasing sampling issues with increasing ligand size. All calculations contain many windows where replicate runs produce significantly different median gradients, meaning that none of our calculations are strictly converged, even after 30 ns sampling time per window. We emphasize that our results would be judged as “converged” by the standards of most of the literature; the reported lack of convergence is due to our strict criterion. In Section S7, we reanalyze results from Alibay et al.^97,128^ to demonstrate that most literature ABFE results are likely also unconverged by this criterion. This confirms that replicate runs are essential for reliable free energy estimates and uncertainty quantification for the bound leg runs, and many free leg runs, at least without improved sampling.^46,70,71^ In accordance with Wan et al., this suggests that the optimal number of replicates for a given sampling time is likely to be the maximum number of replicates which remain long enough to achieve sufficient equilibration.^127^ We note that averaging results from multiple replicate runs which become trapped in separate regions of configuration space is not theoretically rigorous,^129^ because there is no guarantee that the distribution among these conformational regions is not biased by the starting conformations. However, this approach appears robust in practice.^70^

The Gellman–Rubin diagnostic is a popular metric used to diagnose convergence in MCMC simulations based on the difference between intrarun and inter-run variances.^80^ However, we found that the tendency of the uncertainties not to decrease with increasing sampling time (the tendency of systems to remain trapped in local minima) meant that this diagnostic was not useful for determining the termination point of our simulations. Effectively, the convergence of our simulations often did not improve with increasing sampling time, and therefore the degree of convergence was not a useful stopping criterion. We note that a more recent version of the Gelman–Rubin diagnostic may be more useful for this purpose.^130^ Still, we found this diagnostic to be very useful for highlighting windows where substantial sampling problems occurred (Section S13).

#### 4.1.2 The Standard Deviations of the Gradients Equilibrate Quickly, while the Standard Errors Equilibrate Slowly

**Figure 4:**
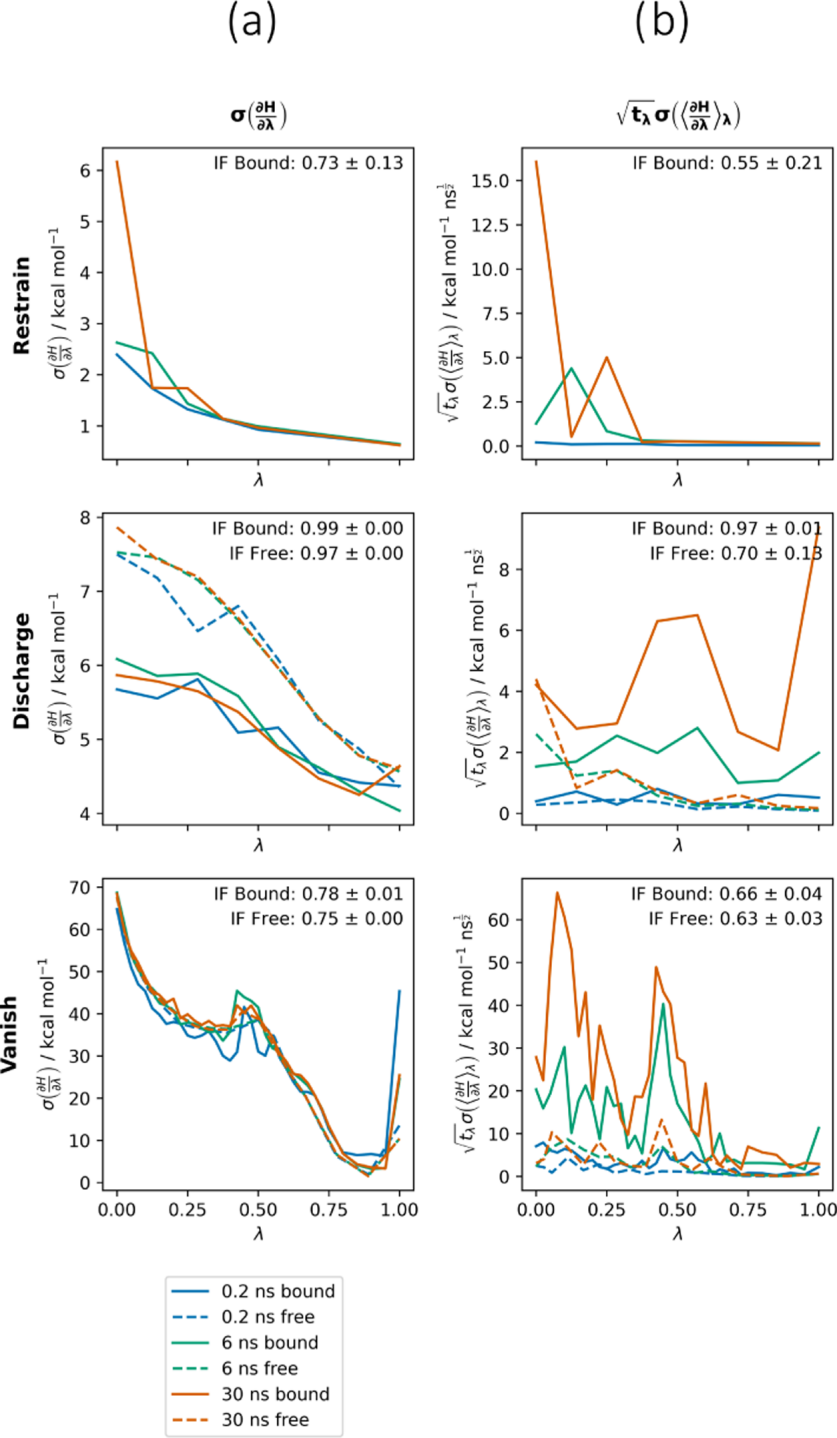
(a) Standard deviation of the gradient and (b) time-normalized standard error of the mean gradient against λ for all calculation stages for PDE2a. Improvement factors (IF, eq 15) are computed with respect to equally spaced λ-windows.

The standard deviation of the gradient  and the time-normalized standard error of the mean gradient  are plotted against λ for PDE2a in Figure 4. This illustrates the general trends for all test systems (the plots for all remaining systems are given in Section S8).

The standard deviations are in excellent agreement between the 0.2, 6, and 30 ns runs, illustrating rapid equilibration (Figure 3A). In general, the curves of  are extremely similar between the vanish stages, but  is slightly higher for the discharge legs. This may stem from greater rearrangement of the polar solvent environment than of the less polar binding pocket upon discharging. The shapes of the standard deviation curves are very similar between systems for the vanish stages, with the exception of the bound vanish stage for T4L, which shows a substantially lower standard deviation around λ = 0.5 (Figure S4). This is likely because the volume vacated by the decoupled ligand is not filled by water in the hydrophobic T4L L99A binding site.

In contrast to the standard deviations, the time-normalized standard errors of the means of the gradients appear unequilibrated (Figure 4b). Increasing sampling time produces higher  values which again reflects the fact that the uncertainties do not generally decrease with sampling time. This increase can happen for two reasons; first, because the replicates remain trapped in local minima in configuration space, producing constant  at increased time and hence increased . In other words, our estimate of the statistical inefficiency has failed to converge because no transitions between the relevant conformational minima have been observed. Alternatively, this can be due to replicates exploring new local minima in configuration space, increasing both  and . As expected from the uncertainties in Table S4,  is generally much smaller and more consistent with increasing simulation times for the free legs over the bound legs, reflecting the fact that the protein environment poses substantial sampling challenges which dramatically increase the statistical inefficiency. The exception to this is the free vanish stage for Nutlin, which shows a substantial peak in  around λ = 0.5 which is similar in magnitude between the bound and free legs. This is due to the overlap of intramolecular groups (Section S9).  is dependent on the statistical inefficiency, which is determined by the slowest timescales of exchange between conformational minima which produce different gradients. As a result, it is noisy. Despite the noise, some trends can be observed:  is generally highest between λ = 0 and 0.6 for the bound vanish stages where the ligand is more strongly interacting with the protein environment, but subsequently drops as the ligand becomes weakly interacting and sampling is improved. For the bound restrain stage, there are often peaks at λ = 0 where the restraint is absent and the ligand can explore poses which were not observed during the restraint-fitting simulations.

These observations have consequences for the design of adaptive protocols. Because  converges quickly, minimum variance protocols can be found from short test simulations. These protocols are equivalent to minimum thermodynamic length protocols where the metric does not account for statistical inefficiency. Because  equilibrates slowly, minimum standard error of the mean protocols cannot easily be found from short simulations, and require adaptive protocols. These protocols are equivalent to minimum thermodynamic length protocols where the metric accounts for statistical inefficiency.

If we wish to use the MBAR free energy estimator, we must calculate the energies of samples obtained at a given value of λ at all other values of λ. Protocols which adaptively change the number of λ-windows according to eq 18 are then inconvenient because of the need to compute energies at all values of λ at which may be used. This can result in a substantial overhead. Therefore, we select λ values to achieve minimum variance using eq 19 based on a short set of initial simulations of negligible cost.  was determined as a function of λ for a default set of windows using a single replicate run with 0.1 ns sampling per window. This is effectively equivalent to selecting states to achieve equal and sufficient overlap when statistical inefficiency is ignored. We then adaptively allocate simulation time to achieve minimum standard error of the mean according to eq 25.

### 4.2 Performance of Window Spacing, Time Allocation, and Equilibration Detection Algorithms

#### 4.2.1 The Window Spacing Protocol is Reliable and Spacing Impacts Equilibration

**Figure 5:**
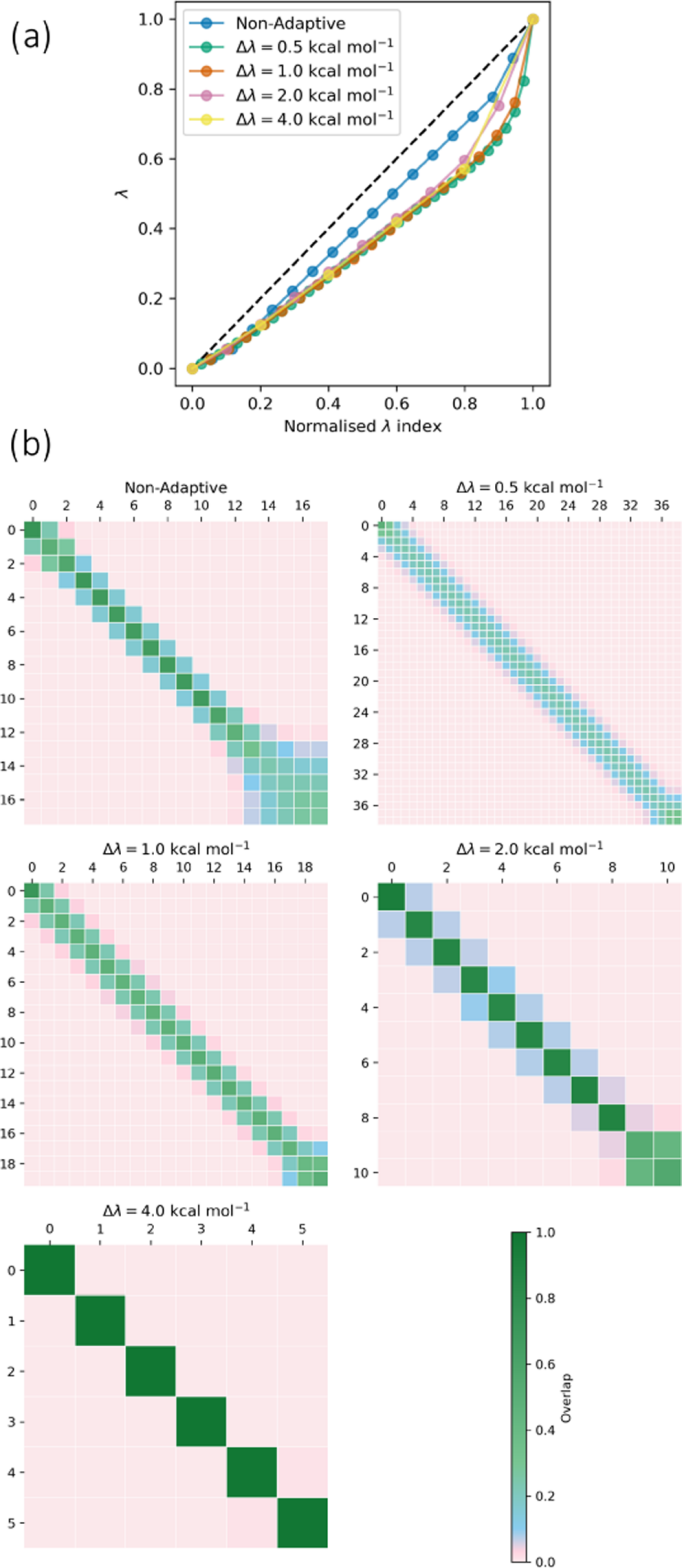
Automated selection of λ windows for the free vanish stage for MIF. (a) λ against normalized λ index  and (b) overlap matrices using a manually optimized λ-schedule, and using the automated method to space windows with thermodynamic speeds of 0.5, 1.0, 2.0, and 4.0 kcal mol^–1^. The automated method produces more consistent off-diagonal overlap than the manually optimized schedule. Plots for all other stages are shown in Section S10.

To independently assess the performance of the automated λ-spacing protocol, we carried out tests on the MIF complex where equal sampling time was allocated to each window (i.e., the window spacing algorithm was used but the adaptive sampling algorithm was not). We generated λ-schedules using thermodynamic speeds of 0.5, 1.0, 2.0, and 4.0 kcal mol^–1^ (eq 19). The cost of the protocol was negligible, but it generated more consistent off-diagonal overlap values than the manually optimized schedule (Figure 5). This was especially true for the vanish stages, where the thermodynamic length varied least linearly as a function of λ. Figure 5a shows λ against the normalized λ index , where a steeper curve of of λ against the normalized index indicates a lower density of windows. Here, the adaptive protocols reduces the density of states close to λ = 1, avoiding the increase in overlap observed for the nonadaptive protocol in this region (Figure 5a). As expected based on Section 4.1.2, the same number of windows were normally selected between the bound and free legs, and the number of windows approximately halved when the thermodynamic speed was doubled (Figure S13). A speed of 2.0 kcal mol^–1^ appeared close to the maximum, because low off-diagonal overlaps were observed (according to the rule-of-thumb that off-diagonal values should not be lower than 0.03).^86^ This is in agreement with the results of Rizzi.^64^

Simulations with the generated λ-schedules showed no visible reduction in the overall standard error of the mean of the free energy change for equivalent sampling time compared to the “manually-optimized” protocol (Figure S14); rather, all schedules other than that with 4.0 kcal mol^–1^ speed showed similar inter-run uncertainties. This is as expected given that the protocol did not account for differences in statistical inefficiency between stages. This may have also been anticipated from the results of Nguyen and Minh, who found that given sufficient overlap, the uncertainty collapsed purely as a function of total sampling time.^131^ However, the 4.0 kcal mol^–1^ thermodynamic speed protocol often showed substantially greater uncertainty, as anticipated from the negligible overlap.

**Figure 6:**
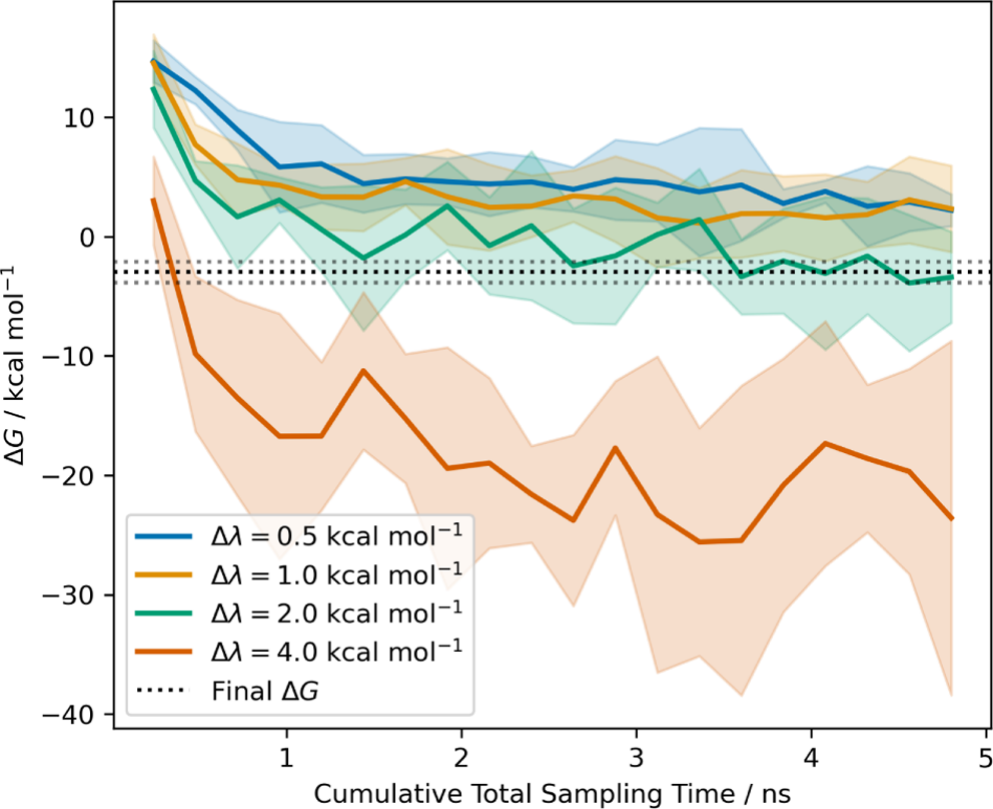
Equilibration of the MIF bound vanish stage against total sampling time with varying λ-window spacing. The mean is shown as a solid line, and shaded regions indicate 95% *t*-based confidence intervals based on inter-replicate differences. Wider spacing leads to accelerated equilibration toward the 30 ns result (black-dotted line with 95% CI shown as sparse dotted lines). The longer-time equilibration and results for the free vanish stage are shown in Section S10.

The most substantial effect of increasing the spacing up to 2.0 kcal mol^–1^ was that equilibration was accelerated (Figure 6) for a given total simulation time. This was discussed by Rizzi.^64^ The effect is intuitive—reducing the number of windows increases the total sampling time per window for a given total simulation time. This produces faster relaxation for a given computational cost. This suggests that for slowly relaxing systems where replica exchange is not used, the thermodynamic speed should be made as large as possible without negatively impacting the MBAR estimate through insufficient overlap. A speed of 2.0 kcal mol^–1^ appears to work well for this system. If this method were used with replica exchange, benefits would still be expected at relatively high thermodynamic speeds,^64^ but the need for a sufficiently high exchange rate may favor lower speeds.^131^

This protocol is effectively a simplified version of those of Minh and Rizzi.^63,64^ We believe that it has a more rigorous basis than more empirical approaches which minimize free energy differences between windows,^65^ because it effectively targets equal divergences of the probability distributions between adjacent states. Adding a constant energy offset to a Hamiltonian would produce a free energy difference while maintaining perfect overlap, and therefore spacing states according to free energy differences has a weak theoretical foundation. This protocol requires that the λ-schedule for the trial simulations is sufficiently dense to faithfully capture variations in  with λ. However, for a reasonably chosen alchemical path (e.g., a reasonable choice of soft-core) there should be no sudden changes in  with λ (Figure 4) meaning that the initial λ-schedule does not have to be particularly dense. The advantage of using a fixed set of initial states is that initial simulations can be run in parallel, which may reduce wall-clock time compared to iterative schemes.^66^ This protocol is also simpler than others based on similar principles.^67^ Given that the automated protocol is simple to implement, appears robust, and produces superior λ-schedules compared to more costly manual selection, we recommend its use in alchemical free energy workflows.

#### 4.2.2 The Adaptive Sampling Scheme Concentrates Sampling Where There are Sampling Issues

To isolate the effect of the adaptive time allocation algorithm, it was tested while λ-schedules were kept constant. This algorithm should provide the greatest advantage when two conditions are met: there is a sampling issue which is restricted to a few windows, and the time scale of the sampling issue is not dramatically longer than a typical λ simulation. This would mean that the default equal-time allocation would be a poor choice, and that the uncertainty could be affordably reduced with additional sampling of problematic windows.

To test whether the adaptive scheme produced benefits in a favorable case, we performed adaptive and nonadaptive runs for the free vanish stage of MDM2-Pip2. This was selected because it had the most favorable SEM-based improvement factor of any stage (0.37 ± 0.13, Figure S6), indicating a sampling issue localized to a few windows. The adaptive and nonadaptive time-allocation runs used the same optimized λ-schedule with speed 1.0 kcal mol^–1^. To provide improved statistics, 20 replicates were used for each protocol. A runtime constant of 1 × 10^–10^ kcal^2^ mol^–2^ ns^–1^ was used for the adaptive runs and the nonadaptive data were truncated slightly above 2 ns per simulation per window to give an equal total computational cost.

**Figure 7:**
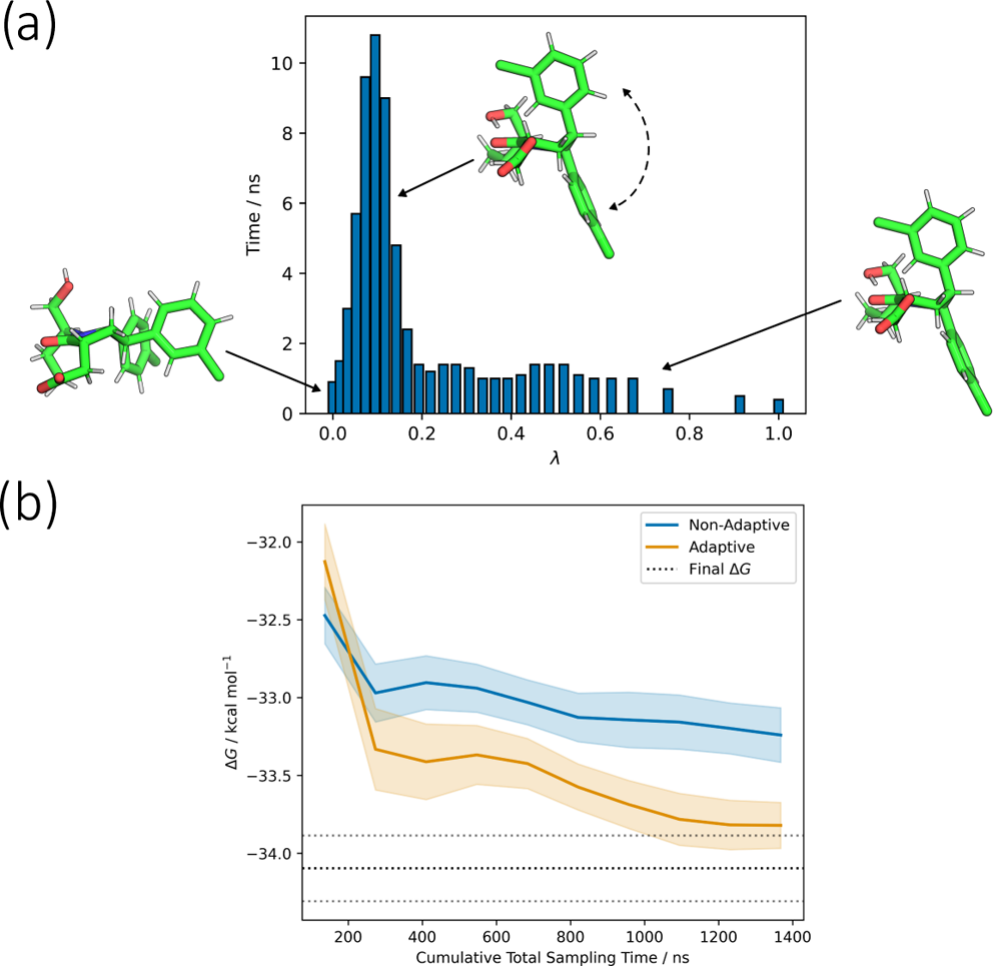
(a) Allocation of sampling time against λ for the Pip2 free vanish leg. Sampling times shown are per-simulation, and 20 replicate simulations were used for each λ-window. The fluctuation between conformations around λ = 0.1 causes divergence of the free energy estimates between runs, and produces a peak in the sampling times. (b) Equilibration of the free vanish stage toward the 30 ns result (black dotted line with sparser dotted lines showing 95% CI). Data was split into 10 blocks before analysis with MBAR. Shaded areas show 95% *t*-based CIs. The adaptive and nonadaptive runs use the same λ-schedule, and therefore the accelerated equilibration is due to the concentration of sampling time around the problematic λ = 0.1 region by the adaptive sampling algorithm.

Figure 7 shows the allocation of sampling time for the adaptive protocol. At λ = 0, the intramolecular Lennard-Jones forces are still fully active and the two chlorophenyl rings of Pip2 remain parallel throughout the simulation. Above λ = 0.2, these forces are substantially weakened, and the ligand relaxes so that the rings point in opposite directions. There is an intermediate regime around λ = 0.1 where the ligand slowly exchanges between both conformations, substantially increasing the uncertainty of the free energy differences between runs. This produces a spike in the allocated simulation time. The main effect of the differing allocation of sampling time is to accelerate equilibration (Figure 7). At longer sampling times, the adaptive protocol produces estimates which are significantly closer to the final 30 ns estimate than the nonadaptive protocol. At very short timescales this trend is reversed, which suggests that fast relaxations are better sampled by the equal-time protocol, while the adaptive protocol more effectively samples the slow exchange between different ring orientations.

We note that while the adaptive algorithm produces desirable improvements in the rate of equilibration, it was intended to reduce uncertainty. It fails to reduce inter-run uncertainty for Pip2, at least at earlier total sampling times (Figure S18). This is explained by the large bias in initial conformations, in which the chlorophenyl rings are always parallel. Concentrating sampling around the λ = 0.1 region in fact initially increased the inter-run uncertainty, because it resulted in a wider, but less biased, spread of free energies; however, uncertainty compared to the long-time result was reduced. If each window were initialized by a conformation which had been thoroughly equilibrated at the given value of λ, this algorithm should also reduce inter-run uncertainty.

We further tested the adaptive protocol on the bound vanish stage of the T4L complex, again with 20 runs per protocol and with a shared λ-schedule selected with a speed of 1.0 kcal mol^–1^ (Section S13). Here, the sampling issue resulted from the occasional water molecule moving into the empty binding site around λ = 1.0. This issue was also noted by Lagardère et al.^34^ The adaptive algorithm allocated substantially more sampling time to the affected windows, but in contrast to the Pip2 example, this did not produce any observable benefit. This is because the time scale of water entry to the binding site is far greater than what was allocated by our algorithm. Hence, we expect that the adaptive algorithm will rarely offer dramatic within-stage improvements, because the timescales of most sampling issues are expected to be much longer than the simulation times that can reasonably be allocated. This is in agreement with the recent work of Yu et al.,^87^ who investigated the performance of a similar algorithm for computing the hydration free energies of trivalent rare earth elements in ionic liquids. They found that the algorithm offered substantial reductions in uncertainty at equivalent computational cost for the Coulombic contribution at 600 K. However, at 300 K the time scale of the sampling issue was dramatically increased and the adaptive protocol offered little improvement over equal-time allocation.

One advantage of our time allocation protocol compared to replica-exchange methods is that information does not have to be shared frequently between simulations. This makes the workflow substantially easier to deploy to high-performance computing clusters, where individual simulations can be parallelized across different nodes. If the only metric used to decide simulation time was the inter-replicate Δ*G* uncertainty for a given window, then only the *N* replicates at each window would have to finish before further simulation time could be allocated to that window. However, we also share some information between λ windows, as we require that the total simulation time allocated is no less than half that of the adjacent windows. This ensures that information about problematic regions is effectively shared between nearby windows, but it also increases the coupling between simulations, which now require information from adjacent windows before they can be resubmitted. Despite the reduction in coupling, we note that our protocol is likely to be less efficient than replica-exchange-based methodologies in terms of absolute computing time, especially highly flexible schemes such as ensemble of expanded ensemble.^132^ In principle, there is no reason why this algorithm should not be applied in combination with replica exchange methods; for example, windows with low statistical inefficiencies could be run for less time between exchange attempts than windows with high statistical inefficiencies. However, this is more complicated, since the statistical inefficiency at a given λ window becomes a function of the sampling at all other λ windows and the interwindow exchange probabilities, making it less clear where additional sampling time would be best allocated. Alternatively, windows showing large inter-run Δ*G* deviations could be targeted by enhanced sampling algorithms. For example, if many replicates were used, the low dimensional descriptor which best discriminates between the run mean Δ*G* estimates may be sought and used to guide enhanced sampling.

#### 4.2.3 The Adaptive Sampling Algorithm Behaves Predictably for the Free Legs, and Less Predictably for the Bound Legs

We sought to understand the reproducibility of the allocated sampling times between repeat runs of the entire protocol, and the effect of changing simulation parameters on the time allocated. In this section, all algorithms (adaptive simulation time allocation, automated window spacing, and equilibration detection) were used. Table 2 shows the allocation of sampling time between protocols labeled according to their runtime constant (r, kcal^2^ mol^–2^ ns^–1^), the thermodynamic speed used for window spacing (s, kcal mol^–1^), and the number of independently equilibrated repeat runs per protocol (n). The overall free energies are generally not significantly different to the nonadaptive 30 ns result. There is reasonable consistency between the times allocated for repeats of the r0.005–s1–n5 protocol, showing that similar sampling issues and related uncertainties are detected between overall repeats. Sampling time is mostly concentrated around λ = 0.4 during the bound vanish stage, where water begins to enter the binding site.^103^ In the ideal case, where the uncertainty decreases proportionally to the square root of sampling time, all protocols with the same runtime constant should be allocated equal sampling times, regardless of the number of λ-windows or number of repeats. This appears to be the case for the free legs, where doubling the number of replicates, or halving or doubling the number of λ-windows does not appear to affect the allocated sampling time. In contrast, the bound vanish leg simulations are prone to becoming stuck in local minima and the uncertainties do not generally decrease as expected with additional sampling time (Section 4.1.1). Here, increasing the number of λ-windows (r0.0005–sOrig–n5, r0.0005–s0.5–n5) or the number of replicates (r0.0005–s1–n10) appears to decrease the total allocated sampling time, likely indicating that reduced uncertainty is achieved with equivalent sampling time when the number of independent simulations is greater, in agreement with Wan et al.^127^ However, this trend is not replicated for the bound discharge leg. Increasing the λ-spacing with speeds of 2 and 4 kcal mol^–1^ generally increased the total sampling time, but did not have a reliable effect on the sampling time for the bound stages, possibly indicating lower reliability of sampling issue detection with fewer independent simulations. Surprisingly, the *r*0.0005–*s*4–*n*5 result remained reasonable despite extremely poor overlap (for the bound vanish leg, the highest off-diagonal overlap was 0.02). When the runtime constant was reduced by a factor of 5, the time allocations for the free leg increased by a factor of approximately √5 as expected, although the proportional increase was greater for the bound stages. Overall, this indicates that the algorithm is robust and behaves as expected when uncertainties decrease in proportion to the square root of the sampling time, as assumed. However, when simulations become trapped in local minima, the overall sampling time allocation can be affected by parameters other than just the runtime constant.

**Table 2:** Variation of Allocated Simulation Time with Simulation Parameters for MIF^a^

	parameters			simulation times/ns						
protocol	run time constant/kcal^2^ mol^–2^ ns^–1^	thermodynamic speed/kcal mol^–1^	no. replicates	bound restrain	bound discharge	bound vanish	free discharge	free vanish	total	Δ*G*_bind_^*o*^/kcal mol^–1^
r0.005, s1, n5, repeat 1	0.005	1.0	5	14	111	282	19	62	488	–11.80 ± 1.87
r0.005, s1, n5, repeat 2	0.005	1.0	5	21	112	306	16	62	517	–11.37 ± 0.74
r0.005, s1, n5, repeat 3	0.005	1.0	5	14	126	308	12	46	506	–11.28 ± 1.66
r0.005, sOrig., n5	0.005	default spacing	5	6	126	196	12	44	384	–12.12 ± 1.07
r0.001, s1, n5	0.001	1.0	5	98	311	1286	32	114	1840	–11.37 ± 0.59
r0.005, s1, n10	0.005	1.0	10	14	112	185	17	66	394	–12.27 ± 0.88
r0.005, s0.5, n5	0.005	0.5	5	6	100	190	15	54	366	–12.05 ± 1.56
r0.005, s2, n5, repeat 1	0.005	2.0	5	8	324	406	14	52	803	–9.52 ± 0.67
r0.005, s2, n5, repeat 2	0.005	2.0	5	17	430	257	12	56	772	–11.15 ± 1.56
r0.005, s4, n5	0.005	4.0	5	12	79	551	16	62	720	–9.47 ± 1.91

a Δ*G*_bind_^*o*^ shown with with 95% *t*-based confidence intervals. In the protocol names, r denotes the run time constant (kcal^2^ mol^–2^ ns^–1^), s denotes the thermodynamic speed (kcal mol^–1^) used to determine the λ-window spacing, and n denotes the number of replicates in each ensemble run. sOrig denotes the use of the default λ-spacing. A detailed breakdown of the calculated free energies is given in Table S3.

We note that the ability of the adaptive algorithm to detect sampling issues is highly dependent on using diverse starting conformations. We performed two adaptive runs of the T4L system with 5 replicate runs, a runtime constant of 0.0005 kcal^2^ mol^–2^ ns^–1^, and λ-windows spaced with a speed of 1 kcal mol^–1^. The first protocol used the default of independently equilibrated (in the fully interacting state) structures for each repeat, while the second protocol used the input structure generated for the first repeat for all repeats. A dramatically greater allocation of sampling time for the bound vanish stage of the first protocol (458 ns) was observed compared to the second protocol (16 ns). This suggests that using different starting velocities alone may be insufficient to sample diverse local minima in the initial stages of the adaptive runs. However, the shared starting structure protocol produced an overall result (−5.26 ± 0.35 kcal mol^–1^) in better agreement with experiment (−5.19 ± 0.16 kcal mol^–1^) than the diverse starting structure protocol (−4.33 ± 0.67 kcal mol^–1^), perhaps suggesting that entry of any waters to this binding site is an artifact of simulation.

#### 4.2.4 Detecting Equilibration Based on Multiple Replicates Improves Reliability

**Figure 8:**
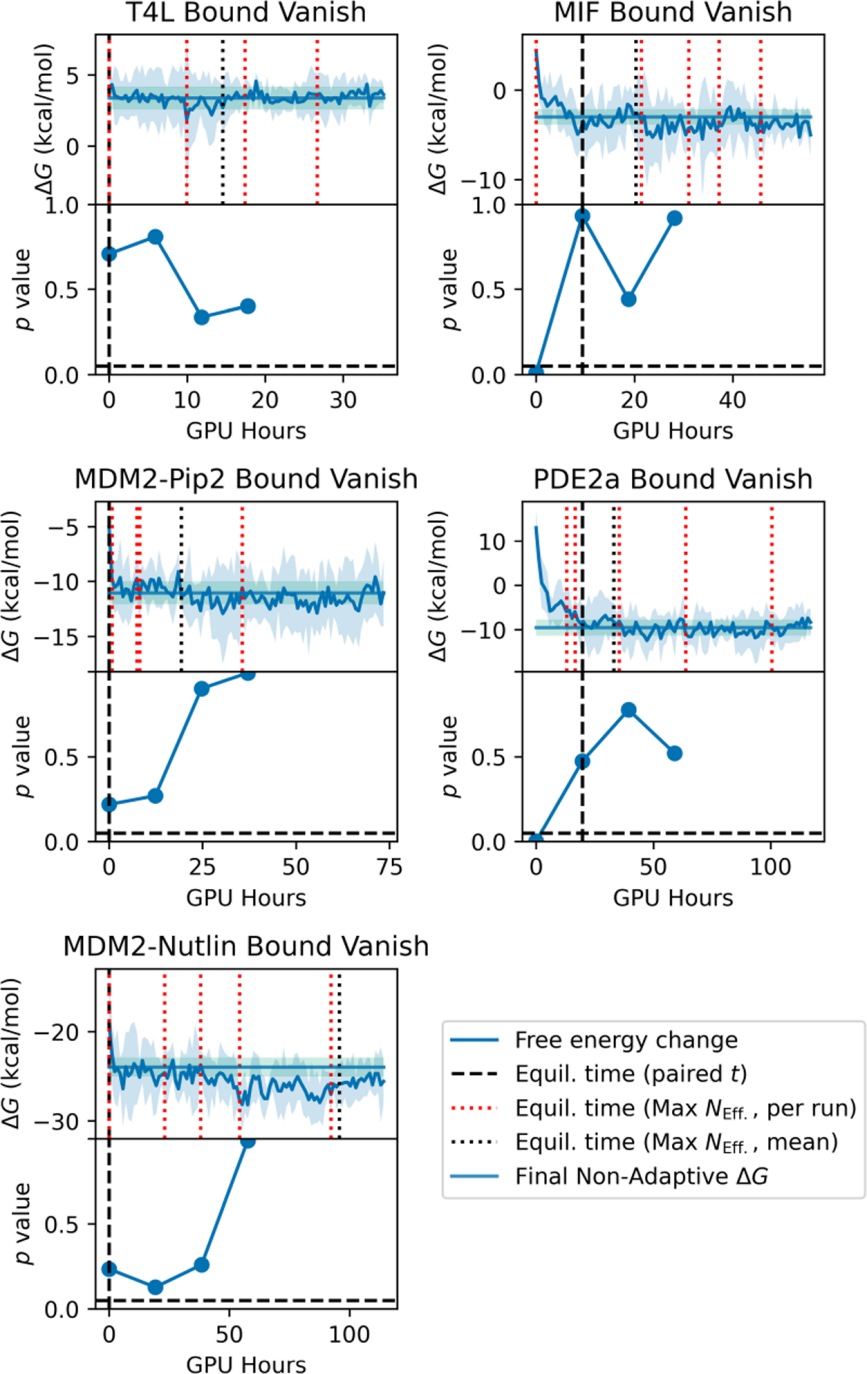
Selection of equilibration times for the bound vanish stage by the paired *t*-test method and Chodera’s method (applied to both the mean trace and individual replicates).^77^ The upper windows show traces of Δ*G* obtained by dividing the data up into 100 equal blocks and running MBAR on each. Shaded areas indicate 95% inter-run *t*-based confidence intervals. Final Non-Adaptive Δ*G* are taken from the nonadaptive 30 ns runs. Lower windows show the *p*-values obtained by truncating the data up to the time shown and performing paired *t*-tests on the first 10% and last 50% of the remaining data. The first *p*-value > 0.05 is used as a heuristic to indicate equilibration.

We assessed our ensemble-based equilibration detection method based on its performance on the bound vanish stage for all initial test systems (Figure 8). These were run adaptively with the parameters given in Section 4.3, meaning that the adaptive window spacing and time allocation algorithms were used in combination with the equilibration detection heuristic. This stage was chosen as it displayed the most pronounced equilibration behavior. In particular, the MIF and PDE2a complexes showed pronounced initial transients, which were successfully removed by the paired *t*-test method. For all other systems, any initial transients were substantially smaller and this method discarded no time to equilibration. Inspection of the equilibration times for all stages showed that the paired *t*-test method rarely discards any data when there is no visible initial transient (Figure S24). This is in contrast to the popular method of Chodera which often discards a substantial amount of data even when there is no obvious initial transient, even when it is applied to the mean trace (see MDM2-Nutlin bound vanish in Figure 8, T4L bound restrain in Figure S24). We compared the absolute differences between the Δ*G* estimates obtained with both equilibration detection methods to the final 30 ns nonadaptive bound vanish results (Figure S25). In every case, the difference is smaller for the paired *t*-test, which could be due to reduced variance arising from retaining more of the data. However, we have a small sample size of 5, and there is no evidence for a significant difference between the two methods at 95% confidence based on the Wilcoxon signed-rank test (*p* = 0.06).

The paired *t*-test method is only applicable to an ensemble of simulations, but Chodera’s method has the advantage that it can be applied to single runs as well as the mean trace. However, applying Chodera’s method to the individual runs for the bound vanish stages reveals substantial variability of the times discarded, generally ranging from no data discarded to nearly all data removed. This is concerning because we care about removing systematic bias in our ensemble result, where the bias results from a relaxation process shared by all runs. We expect this to have a comparable time scale between runs, and therefore replicates should have similar equilibration times. It appears that explicitly using information about the inter-run reproducibility of the transient in the paired *t* method increases robustness. During initial testing, we found the paired *t*-test method to be substantially more sensitive to the presence of initial transients than an unpaired *t*-test on the initial and final portions of the data.

While the performance of the paired *t*-test method appeared promising, we note several potential limitations. First, it is not a rigorous statistical test but a heuristic; *p* > 0.05 shows no significant evidence for drift of the free energy estimate, but we take it to mean that there is no drift of the free energy estimate (“The Fallacy of the Transposed Conditional”).^133^ The use of a parametric test may not be justified. Furthermore, like all equilibration detection methods we are aware of, this method requires that initial simulations have been run on a time scale comparable to that of the initial transient; very slow equilibration will not be apparent from very short simulations. Our workflow likely depends on the correlation between slow initial relaxation and large inter-run uncertainty, which means that sufficient sampling time is allocated to the bound vanish legs to allow the initial transients to be detected and removed. In addition, the method is sensitive to parameters such as the number of *t*-tests performed and the number of replicate runs. With increasing numbers of tests, the chances of falsely “detecting equilibration” early increase. This could be mitigated by taking the equilibration point to be the first test with *p* < 0.05 starting from the test with most truncation. With more replicate runs, the power of the test increases and a lack of equilibration is more likely to be detected.

One deficiency of Chodera’s method is that it never explicitly signals lack of equilibration, but rather selects an optimal truncation point given the data that has already been collected. The paired *t*-test method could in principle detect a lack of equilibration, triggering the allocation of additional simulation time, although this was not observed for this test set.

### 4.3 Overall Performance of an Optimized Adaptive Protocol

“Optimal” parameters were selected for the adaptive algorithms based on the tests described above. We selected a runtime constant of 0.0005 kcal^2^ mol^–2^ ns^–1^ as this generally produced runs of sufficient length to match the nonadaptive 30 ns results. We retained our default of 5 replicate runs as this seemed to represent a reasonable compromise between fast equilibration and reliable detection of sampling issues, although we did not investigate this in detail. Because we did not use replica exchange and hence did not have to consider replica mixing, we spaced λ-windows with a large thermodynamic speed of 2 kcal mol^–1^ to accelerate equilibration. Finally, we retained the default equilibration detection parameters as these appeared to deliver robust performance.

As an initial test of overall performance, we applied the adaptive protocol to the set of initial test systems. This is effectively a “training” set as we used it to select the adaptive parameters, and it is therefore not representative of prospective performance. Hence, we then assessed the “optimized” protocol on an unseen “test” set.

#### 4.3.1 The Adaptive Protocol Accelerates the Equilibration of the Initial Test Set

For all systems, only two λ windows were selected for turning on the restraints, but high overlap (>0.2) was observed for this stage (Figure S26). This reflects the fact that the restraint selection algorithm selects restraints which minimally perturb the ligand’s native interactions. As expected from the  plot (Figure S4), similar λ-schedules were usually generated for the same transformation in the free and bound legs, meaning that reasonable schedules for the bound leg may be determined from the cheaper free leg initial test simulations. However, there were exceptions—for example, more windows were used for the PDE2a free discharge leg than the bound discharge leg due to the greater variance of the gradient observed in the free leg. A detailed breakdown of the sampling time allocations is provided in Section S18. Generally, the compute time was concentrated in the bound discharge and especially the bound vanish stages, and increased with increasing ligand size.

The “optimized” adaptive protocol produced extremely similar results to the long time 30 ns and manually optimized 6 ns nonadaptive protocols (Table 3). This was despite using over 3 times less GPU time than the 6 ns protocol, over 14 times less than the 30 ns protocol (Table S5), and requiring no manual tweaking of the equilibration times of λ-schedules. We note that this is not an entirely fair test, as we used some of these systems to optimize the adaptive algorithm parameters in the previous sections. However, this provides further reassurance that the parameters and overall protocol are robust, and shows that the adaptive protocol produces equivalent results to a manually optimized nonadaptive protocol at lower computational cost.

**Table 3:** Predicted Δ*G*_Bind_^*o*^ for Initial Test Systems with Adaptive and Long-Run Non-Adaptive Protocols^a^

	“optimized” adaptive	30 ns non-adaptive
T4L	–4.28 ± 0.73	–4.03 ± 0.80
MIF	–10.54 ± 0.78	–10.48 ± 1.14
MDM2-Pip2	–13.98 ± 1.67	–13.94 ± 0.86
PDE2a	–18.36 ± 1.49	–16.96 ± 1.97
MDM2-Nutlin	–16.14 ± 1.92	–16.92 ± 1.98

a All quantities in kcal mol^–1^. Calculation uncertainties stated as 95% confidence intervals based on the variance of 5 replicate runs, assuming Gaussian distributions. Symmetry corrections are included (Section S5). A detailed breakdown of the components of the calculated binding free energies is given in Table S4.

**Figure 9:**
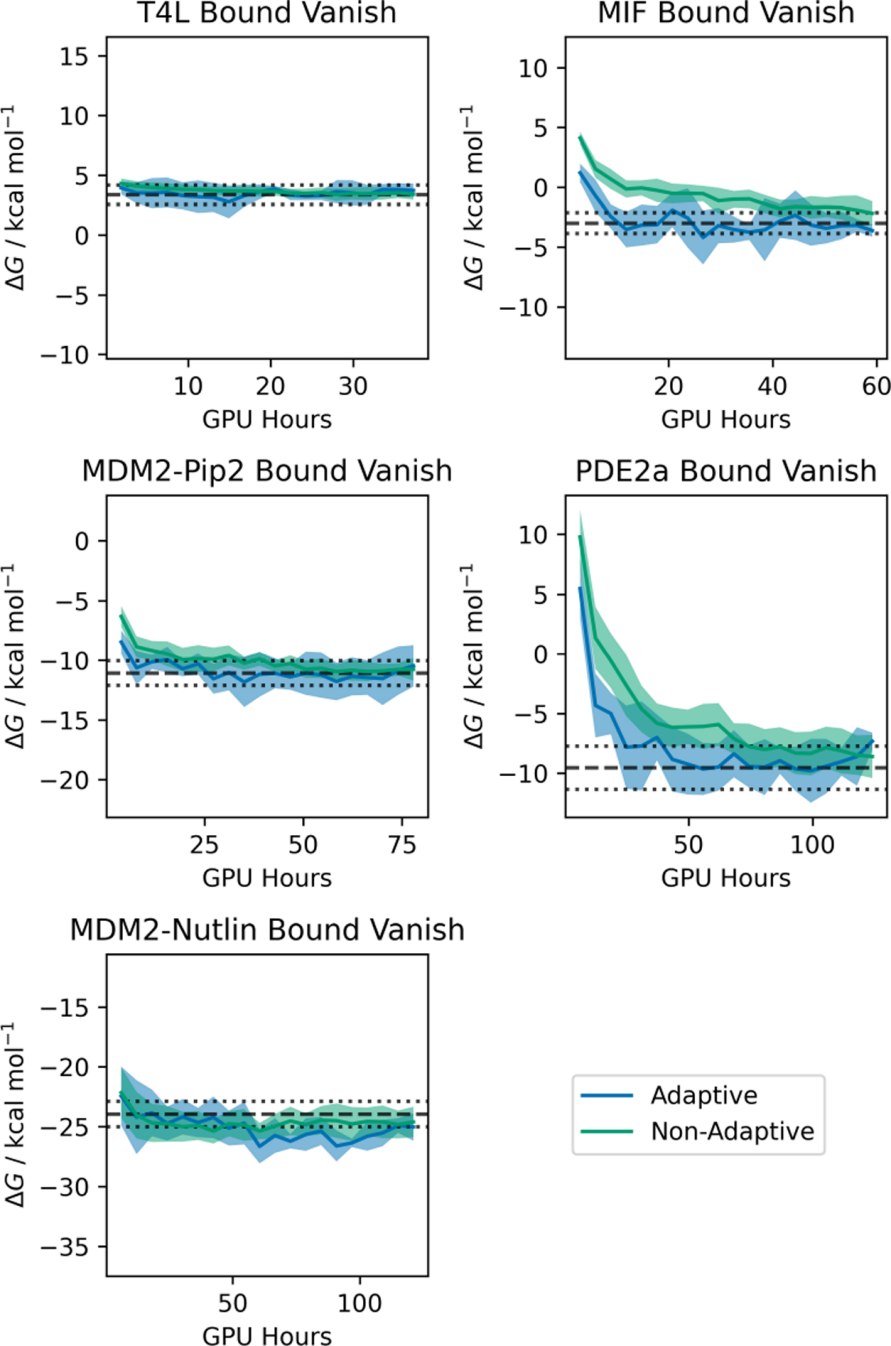
Estimated Δ*G* against sampling time for the bound vanish stages of the initial test systems. Data were split into 20 equal blocks and MBAR was run on each block. “adaptive” refers to the “optimized” adaptive protocol, and the nonadaptive results were obtained by averaging the nonadaptive 6 and 30 ns runs to more clearly show the differences in equilibration behavior. The data for the nonadaptive runs was truncated before analysis so that the per-stage computational costs were equal to the adaptive calculation. For this reason the 95% *t*-based confidence intervals (shaded areas) are expected to be a factor of √2 smaller for the nonadaptive protocol. The final nonadaptive Δ*G* is taken from the 30 ns nonadaptive result (black dotted line with 95% CIs shown by sparser dotted lines). The *y*-axis spacings are the same for all plots. Plots for all stages are shown in Section S20.

We may also have tried to save compute simply by shortening the nonadaptive runs. To investigate the feasibility of this, we compared the free energy estimates obtained from the adaptive and nonadaptive protocols at equivalent computational time, ignoring equilibration and retaining all data. Figure 9 shows that the adaptive protocol produces substantial increases in the rate of equilibration for the bound vanish stages for MIF and PDE2a, the two systems with the most pronounced initial transients. As these stages make the dominant contributions to overall calculation cost, this produces accelerated equilibration of the entire data set (Figure S33). Because the allocated sampling times are not sharply peaked for these stages (Figure S28), we attribute this improvement to the wider λ spacing. Manually selecting such widely spaced windows while retaining sufficient overlap would be extremely labor-intensive, illustrating the potential of the adaptive protocol to minimize human, as well as computer time. Even if the nonadaptive protocols were run for the same total compute time as the adaptive protocol for the bound vanish stages, Figure 9 shows very long equilibration times would be required for MIF and PDE2a. Identifying the requirement for such long equilibration times would be time-consuming; alternatively, uniformly applying very long equilibration times would be wasteful. In contrast, the adaptive procedure only discards data to equilibration for the problematic MIF and PDE2a systems (Figure 8). This suggests inherent advantages to the automated, adaptive protocol even when the total computational cost is equivalent.

Although the adaptive algorithm produced improvements in the rate of equilibration, we found no clear differences between the uncertainty in the free energy estimates at equivalent computational effort either within stages (Figure S32), or over entire calculations (Figure S34). This likely reflects two facts: that the -based improvement factors are fairly close to 1 (Figure S5), indicating little scope for improvement with adaptive time allocation; and that allocating time to problematic windows often does not reduce uncertainties because individual runs become stuck in local minima, indicating that the timescales of sampling issues are too long. An alternative approach would be to allocate more replicates to problematic λ windows, but this would reduce the rate of equilibration.

Despite the lack of uncertainty reduction, the algorithm successfully allocated sufficient time to achieve equilibration in the bound vanish legs, while allocating dramatically less time to most free stages. This again suggests a correlation between slow equilibration and large inter-run uncertainty.

#### 4.3.2 The Adaptive Protocol Shows Robust Performance on an “Unseen” Test Set

We sought to test the robustness of our workflow using a set of new systems which were not used to select the “optimal” workflow parameters. We selected the Cyclophilin-D set from Alibay et al.’s study of ABFE in the fragment optimization process.^97^ Excluding ligand 4 (because its affinity fell outside the SPR detection limits) produced a set of 9 fragments and merged molecules with a wide dynamic range.^134^ Alibay et al.’s results were reanalyzed and plotted excluding this ligand in Section S21.

First, we performed nonadaptive runs for all ligands using the same number of λ-windows for each stage as Alibay et al. We allocated 5 ns per window with 1 ns discarded to equilibration, which reflects common practice in the literature. Reassuringly, the nonadaptive SOMD runs produced a very similar correlation with experiment compared to the GROMACS runs of Alibay et al. (*R*^2^ values of 0.81_0.46_^0.91^ and 0.79_0.38_^0.95^ (upper and lower 95% CIs), respectively—see Table S6), despite the allocation of around 4 times less simulation time. As found by Alibay et al., our predictions were systematically more negative than experiment (Table 4). A more detailed analysis and comparison to the results of Alibay et al. is given in Section S21.

**Table 4:** Predicted Δ*G*_Bind_^*o*^ for Cyclophilin D^a^

	adaptive	nonadaptive 5 ns	Alibay	exp. Δ*G*_Bind_^*o*^
Ligand 2	–10.56 ± 1.06	–10.52 ± 1.21	–8.18 ± 0.76	–9.06 ± 0.50
Ligand 3	–5.06 ± 2.78	–4.73 ± 1.21	–4.71 ± 0.27	–2.93 ± 0.50
Ligand 4	–4.92 ± 1.92	–6.93 ± 1.79	–4.14 ± 1.00	–2.90 ± 0.50
Ligand 8	–7.30 ± 0.73	–7.34 ± 1.06	–7.24 ± 0.73	–4.04 ± 0.50
Ligand 14	–14.38 ± 2.17	–15.44 ± 1.39	–12.92 ± 0.54	–11.22 ± 0.50
Ligand 16	–9.27 ± 1.79	–10.49 ± 2.04	–10.54 ± 0.60	–8.42 ± 0.50
Ligand 27	–9.14 ± 1.67	–10.81 ± 1.42	–10.21 ± 1.36	–7.57 ± 0.50
Ligand 39	–13.99 ± 1.97	–13.58 ± 1.09	–12.62 ± 0.59	–8.43 ± 0.50
Ligand 40	–14.72 ± 0.88	–14.51 ± 0.81	–11.78 ± 0.65	–8.08 ± 0.50

a All quantities in kcal mol^–1^. Calculation uncertainties stated as 95% *t*-based confidence intervals based on the variance of 5 replicate runs, assuming Gaussian distributions. “Alibay” results were taken from Alibay et al.,^97^ and the experimental results were taken from Grädler et al., who used surface plasmon resonance.^134^ The experimental uncertainties were assumed to be 0.5 kcal mol^–1^. A detailed breakdown of the components of the calculated binding free energies is given in Table S7.

**Figure 10:**
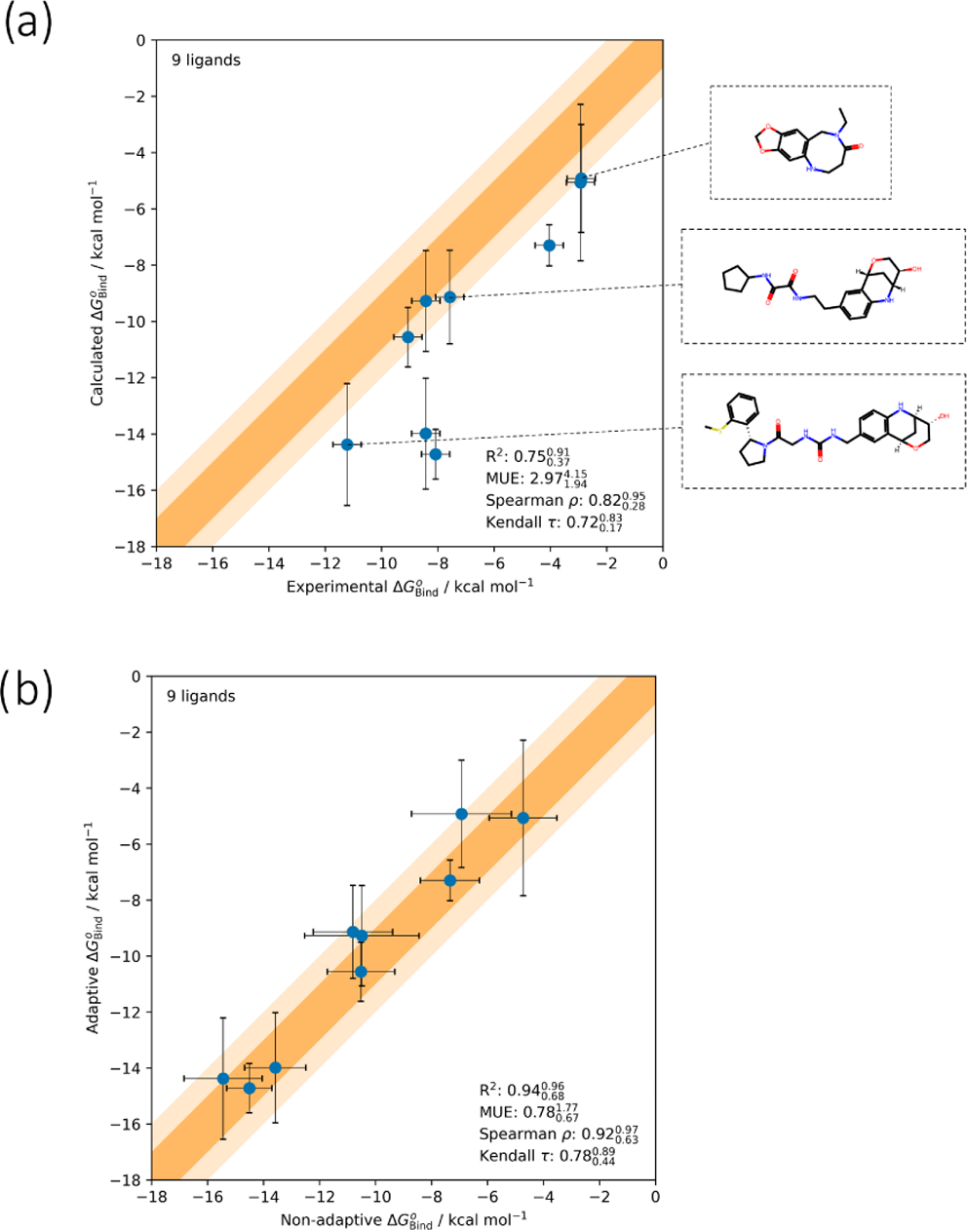
(a) Experimental free energies of binding for Cyclophilin D against predicted free energies of binding obtained using the “optimized” adaptive protocol and (b) comparison of results obtained using the “optimized” adaptive protocol and the nonadaptive protocol. Selected ligands are shown in (a) to illustrate the structural diversity. Experimental free energies were obtained from Grädler et al.^134^ The darker and lighter shaded areas show 1 and 2 kcal mol^–1^ deviations from exact agreement, respectively. Error bars show 95% confidence intervals, which were assumed to be 0.5 kcal mol^–1^ for experiment and calculated from the deviation between 5 replicate runs for the predictions. 95% confidence intervals on statistics were calculated by bootstrapping with 10000 iterations of resampling.

Recalculating all free energies with the “optimized” adaptive protocol produced extremely similar results to the nonadaptive protocol (Figure 10 and Table 4) with good correlation with experiment, albeit for a small set of ligands. There was no significant evidence at 95% confidence for differences in either the uncertainty between the adaptive and nonadaptive runs, nor the unsigned error compared to experiment (*p* = 0.36 in both cases using the Wilcoxon ranked-sign test). A relatively large amount of simulation time was allocated to these calculations using the same parameters as for the initial test systems, because the bound systems were relatively small and inexpensive (around half the cost of the MIF bound system). However, the adaptive protocol was still around 1.5 times cheaper than the nonadaptive protocol, and around 6 times cheaper than the protocol of Alibay et al. Unlike the MIF and PDE2a systems, these complexes equilibrated quickly, meaning that the accelerated equilibration afforded by the adaptive protocol did not provide a substantial advantage (Figure S42). However, the fact that the adaptive protocol produced equivalent results to the nonadaptive protocol on an “unseen” test set suggests that it is robust and that our “optimal” parameters are likely to perform well for other systems. Furthermore, the adaptive protocol performed sensible optimizations without any human intervention, for example reducing the number of λ windows for the restrain stage from 12 (which produced very high overlap) to 2. A more detailed analysis is given in Section S22.

## 5 Conclusions

We have presented an automated workflow for ABFE calculations based on the automated selection of λ windows, the ensemble-based detection of equilibration, and the adaptive allocation of sampling time based on inter-replicate uncertainties in the per-window free energy estimates. Our central conclusions are:

(1) The automated selection of λ windows to give consistent overlap can be achieved using very short initial test simulations with nonoptimal spacing. This is cheap, robust, and simple to implement. Increasing the spacing of windows can accelerate equilibration by reducing the concentration of total sampling time at the start of simulations.
(2) An ensemble-based equilibration detection heuristic based on a paired *t*-test between the free energy estimates at initial and final portions of a run appears robust.
(3) Adaptively allocating sampling time based on the uncertainty in free energy changes between repeat runs increased sampling times where there were sampling issues. The bound vanish leg was usually allocated the most computing time, because it usually involved the most severe sampling issues. In a rare case where the sampling issue had a time scale amenable to simulation, this algorithm accelerated equilibration. However, we generally found no evidence that the adaptive time allocation algorithm reduced uncertainty for equivalent computational cost, likely because the timescales of sampling issues could not be matched by our simulations.
(4) We found reasonable default parameters for all algorithms and tested the performance of our overall workflow on a range of systems. In all cases, the adaptive protocol produced free energy estimates equivalent to the nonadaptive protocols. For systems which showed slow equilibration, the adaptive protocol substantially increased the rate of equilibration, likely due to the wide spacing of λ windows.
(5) We have provided an open-source implementation of all algorithms in the python package A3FE, which is available on GitHub (https://github.com/michellab/a3fe)

We hope this work helps to facilitate the more efficient, automated calculations which will be required to unlock the potential of ABFE calculations for drug discovery.

Here, we have taken the narrow view that the optimal ABFE workflow would produce minimal overall uncertainty in all free energy estimates for a fixed computational cost. However, practitioners in industrial drug discovery may not need precise estimates of affinities for weak binders, so long as they are correctly identified as weak. It is also likely more useful to accurately predict the affinities of dissimilar ligands with distinct receptor interactions than more similar ligands for which Δ*G*_Bind_^*o*^ may be predicted with more confidence using a machine learning model trained on previous ABFE results. Future work may take a more realistic view of the “optimal” workflow, for example by quickly terminating simulations which show weak binding. This is likely to be robust because, as we have discussed, unequilibrated free energy estimates tend to overestimate affinity. Tighter coupling between free energy protocols and active learning protocols may also be useful—for example, the number of replicas and simulation time may be increased in proportion to the uncertainty of a machine-learned predictor of affinity.

## Data Availability

The A3FE package is available on GitHub at https://github.com/michellab/a3fe. Example input files, all data analyzed, and analysis code is available at https://github.com/michellab/Automated-ABFE-Paper.

## Supporting Information Available

Detailed descriptions of the preparation of the initial test systems; detailed results, restraint parameters, and analysis of the nonadaptive and adaptive runs for the initial test and Cyclophilin D systems (PDF)
