# Supplementary material for: Automated Adaptive Absolute Binding Free Energy Calculations
Source: J Chem Theory Comput. 2024 Sep 10;20(18):7806–28. doi: 10.1021/acs.jctc.4c00806 (PMC11428140; doi:10.1021/acs.jctc.4c00806)
Supplement: Supplementary file 1 — ct4c00806_si_001.pdf [file ct4c00806_si_001.pdf]

Supplementary Information for Automated Adaptive Absolute Binding Free Energy Calculations

Finlay Clark,† Graeme R. Robb,‡ Daniel J. Cole,¶ and Julien Michel*,†

†*EaStCHEM School of Chemistry, University of Edinburgh, David Brewster Road, Edinburgh EH9 3FJ, United Kingdom*

‡*Oncology R&D, AstraZeneca, Cambridge CB4 0WG, United Kingdom*

¶*School of Natural and Environmental Sciences, Newcastle University, Newcastle upon Tyne NE1 7RU, United Kingdom*

E-mail: julien.michel@ed.ac.uk

S1 Uncertainties of TI, Zwanzig, and BAR in the Limit of Infinitely Close States

Under the assumption of infinitely closely-spaced intermediate states, the uncertainties of the TI, Zwazig, and BAR estimates become equivalent. This discussion is similar to that given by Nguyen and Minh.[1]

S1.1 TI

In the theoretical limit of infinitely many states, the free energy change can be calculated with thermodynamic integration according to

$$\widehat{\Delta F} = \int_0^1 \left\langle \frac{\partial H}{\partial \lambda} \right\rangle_\lambda \mathrm{d}\lambda, \tag{S1}$$

where $\widehat{\Delta F}$ denotes the estimated free energy change and $\langle ... \rangle_\lambda$ denotes an average obtained at a fixed value of λ.

Assuming independent sampling at different values of λ, the total variance can be obtained by integrating the uncertainties at each value of λ

$$\sigma^2(\widehat{\Delta F}) = \int_0^1 \sigma^2 \left(\left\langle \frac{\partial H}{\partial \lambda} \right\rangle_\lambda \right) \mathrm{d}\lambda. \tag{S2}$$

S1.2 Zwanzig

The free energy difference between states 0 and 1 can be calculated using samples from state 0 using the Zwanzig equation

$$\widehat{\Delta F} = -\beta^{-1} \ln \left\langle e^{-\beta \Delta u(\boldsymbol{x})} \right\rangle_0, \tag{S3}$$

where $\langle ... \rangle_0$ denotes an average over samples obtained at state 0, $\beta = \frac{1}{k_\mathrm{B}T}$, and $\boldsymbol{x}$ is a point in configuration space. When the states are close enough in λ such that $\beta \Delta u(\boldsymbol{x}) << 1$ can be assumed, the exponential term in Equation S3 can be approximated using a first-order Taylor expansion

$$\widehat{\Delta F} \approx -\beta^{-1} \ln \langle 1 - \beta \Delta u(\boldsymbol{x}) \rangle_0 \tag{S4}$$

$$\approx -\beta^{-1} \ln(1 - \langle \beta \Delta u(\boldsymbol{x}) \rangle_0) \,. \tag{S5}$$

Taking another Taylor expansion yields

$$\widehat{\Delta F} \approx \langle \Delta u(\boldsymbol{x}) \rangle_0 \,. \tag{S6}$$

In the case of several intermediate states, Equation S6 could be applied repeatedly to obtain the overall free energy change

$$\widehat{\Delta F} \approx \sum_{k=1}^{K-1} \langle \Delta u_{k+1,k}(\boldsymbol{x}) \rangle_k \,, \tag{S7}$$

where k is the state number and there are K states. In the limit of infinitely close states this becomes equivalent to Equation S1 and hence the uncertainty is also given by Equation S2.

S1.3 BAR

The Bennett Acceptance Ratio (BAR) can be understood as reweighting from the mixture distribution, π_{Mix}, constructed with samples from two states.[2,3]

$$\widehat{F_1} = -\beta^{-1} \ln \left\langle \frac{e^{-\beta u_1(\boldsymbol{x})}}{\widehat{\pi}_{\text{Mix}}(\boldsymbol{x})} \right\rangle_{\pi_{\text{Mix}}} , \tag{S8}$$

$$\widehat{\pi}_{\text{Mix}}(\boldsymbol{x}) = \frac{N_0}{N_0 + N_1} e^{-\beta u_0(\boldsymbol{x}) + \beta \widehat{F_0}} + \frac{N_1}{N_0 + N_1} e^{-\beta u_1(\boldsymbol{x}) + \beta \widehat{F_1}}, \tag{S9}$$

where N_k is the total number of samples from state k. Setting $\widehat{F_0} = 0$ and assuming $N_0 = N_1 = N$

$$\widehat{\Delta F} = -\beta^{-1} \ln \left\langle \frac{e^{-\beta u_1(\boldsymbol{x})}}{\frac{1}{2}\left(e^{-\beta u_0(\boldsymbol{x})} + e^{-\beta u_1(\boldsymbol{x}) + \beta \widehat{\Delta F}}\right)} \right\rangle_{\pi_{\text{Mix}}} . \tag{S10}$$

Dividing the top and bottom of the fraction by $e^{-\beta u_0(\boldsymbol{x})}$ yields

$$\widehat{\Delta F} = -\beta^{-1} \ln \left\langle \frac{e^{-\beta \Delta u(\boldsymbol{x})}}{\frac{1}{2}\left(1 + e^{-\beta \Delta u(\boldsymbol{x}) + \beta \widehat{\Delta F}}\right)} \right\rangle_{\pi_{\text{Mix}}} . \tag{S11}$$

As the states become infinitely close, $\beta\Delta u(\boldsymbol{x})$ and $\beta\Delta\widehat{F}$ become much less than 1. This justifies the use of first-order Taylor expansions of the exponentials

$$\widehat{\Delta F} = -\beta^{-1}\ln\left\langle \frac{1-\beta\Delta u(\boldsymbol{x})}{1-\frac{1}{2}(\beta\Delta u(\boldsymbol{x})-\beta\widehat{\Delta F})} \right\rangle_{\pi_{\text{Mix}}}. \tag{S12}$$

Using the fact that $\frac{1}{1-x} = 1 + x$ for $x << 1$ (where x is $\frac{1}{2}(\beta\Delta u(\boldsymbol{x}) - \beta\widehat{\Delta F})$)

$$\widehat{\Delta F} = -\beta^{-1}\ln\left\langle (1-\beta\Delta u(\boldsymbol{x}))\left(1+\frac{1}{2}(\beta\Delta u(\boldsymbol{x})-\beta\widehat{\Delta F}\right)\right\rangle_{\pi_{\text{Mix}}} \tag{S13}$$

$$= -\beta^{-1}\ln\left\langle 1-\frac{1}{2}\beta\Delta u(\boldsymbol{x})-\frac{1}{2}\beta\widehat{\Delta F}\right\rangle_{\pi_{\text{Mix}}}, \tag{S14}$$

where we have ignored the cross terms (such as $(\beta\Delta u(\boldsymbol{x}))^2$), which are negligible. As the two states become infinitely close in λ, the weights of each state in π_{Mix} become equal, and $\langle ...\rangle_{\pi_{\text{Mix}}}$ becomes the arithmetic mean over the two states. Furthermore, the configurations sampled in each state become indistinguishable, meaning that $\langle ...\rangle_{\pi_{\text{Mix}}} \approx \langle ...\rangle_0 \approx \langle ...\rangle_1$. Arbitrarily choosing state 0, and taking the first-order Taylor expansion of the logarithm yields

$$\widehat{\Delta F} \approx \langle \Delta u(\boldsymbol{x})\rangle_0, \tag{S15}$$

which is the same as Equation S6., Hence, the uncertainty is given by Equation S2, as before.

S2 The Relationship Between the Variance of the Gradient and Overlap

The variance of the gradient of the free energy with respect to λ, sampled at some value of λ, is

$$\text{Var}\left(\frac{\partial H}{\partial\lambda}\right) = \left\langle \left(\frac{\partial H}{\partial\lambda}\right)^2 \right\rangle_\lambda - \left\langle \frac{\partial H}{\partial\lambda}\right\rangle_\lambda^2 \tag{S16}$$

where $H(\boldsymbol{x};\lambda)$ is the Hamiltonian which depends on the sampled point in configuration space, $\boldsymbol{x}$, and the coupling parameter, λ. $\langle ... \rangle_\lambda$ denotes an average at a given value of λ.

As discussed by Blondel,[4] offsetting all energies at a given value of λ by a constant does not affect the variance. For simplicity, the offset is chosen to be $F(\lambda)$, the free energy at a given value of λ, and the new Hamiltonians are denoted by H'. Hence

$$\mathrm{Var}\left(\frac{\partial H}{\partial \lambda}\right) = \left\langle \left(\frac{\partial H'}{\partial \lambda}\right)^2 \right\rangle_\lambda - \left\langle \frac{\partial H'}{\partial \lambda} \right\rangle_\lambda^2 \tag{S17}$$

We also note that

$$e^{-\beta H'(\boldsymbol{x},\lambda)} = e^{-\beta H(\boldsymbol{x},\lambda)+\beta F(\lambda)} = p(\boldsymbol{x},\lambda) \tag{S18}$$

where $p(\boldsymbol{x},\lambda)$ is the normalised probability of sampling phase-space point $\boldsymbol{x}$ at a given value of λ.

Because all λ states have been offset by their free energies, the free energy changes between the new states is 0, and therefore $\left\langle \frac{\mathrm{d}H'}{\mathrm{d}\lambda} \right\rangle_\lambda = 0$, simplifying Equation S18

$$\mathrm{Var}\left(\frac{\partial H}{\partial \lambda}\right) = \left\langle \left(\frac{\partial H'}{\partial \lambda}\right)^2 \right\rangle_\lambda \tag{S19}$$

Noting that

$$\frac{\partial H'}{\partial \lambda} = \frac{\frac{\partial H'}{\partial \lambda} e^{-\beta H'}}{e^{-\beta H'}} = -\frac{1}{\beta}\frac{\partial \log p(\mathbf{x},\lambda)}{\partial \lambda} \tag{S20}$$

Equation S19 can be rewritten:

$$\beta^2 \mathrm{Var}\left(\frac{\partial H}{\partial \lambda}\right) = \int \left(\frac{\partial \ln p(\mathbf{x},\lambda)}{\partial \lambda}\right)^2 p(\mathbf{x},\lambda)\mathrm{d}\mathbf{x} \tag{S21}$$

This tells us that the dimensionless variance of the gradient is the Fischer information. Rewriting this equation

$$\beta^2 \mathrm{Var}\left(\frac{\partial H}{\partial \lambda}\right) = \left\langle \left(\frac{1}{p(\mathbf{x},\lambda)}\frac{\partial p(\mathbf{x},\lambda)}{\partial \lambda}\right)^2 \right\rangle_\lambda \tag{S22}$$

shows that the dimensionless variance of the gradient is the expected value of the squared relative probability change when λ is varied.

S3 Details of Structure Preparation for Initial Test Systems

Where details of structure preparation are omitted below, the steps described in Section 3.1 were followed.

S3.1 T4L

The complex of benzene with the L99A mutant of T4 lysozyme was prepared from PDB ID 4W52 as described in Section 3.1.[5]

S3.2 MIF

The complex of human macrophage migration inhibitory factor (MIF) with the ligand MIF180 was prepared as described by Clark et al..[6] The only differences were the use of Open Force Field 2.0.0 for the small molecule, rather than GAFF2.11, and the use of a rhombic dodecahedral box, rather than a cubic box.

S3.3 MDM2-Pip2

For the complex of MDM2 (with truncated lid - "Short") with Pip2, AMBER prm7 and rst7 files were obtained from Mendoza-Martinez et al..[7] The only modifications made were the reparameterisation of the ligand with Open Force Field 2.0.0 with AM1-BCC partial charges (rather than the original GAFF), and the resolvation using a rhombic dodecahedral box, as described in Section 3.1. The experimental binding affinity was taken from the "17-125" column for the Pip-2 ITC data in Table 1 of Michelsen et al..[8]

S3.4 PDE2a

A PDB structure for Phosphodiesterase 2a in complex with ligand "P10" was obtained from Huggins,[9] specifically from https://github.com/djhuggins/Holoware-TestCases/blob/main/PDE2/ff14_tip3[illegible] Due to all water molecules having incorrect angles for TIP3P, water hydrogens were removed and reintroduced using tleap from ambertools 22.0. Otherwise, preparation was performed as described in Section 3.1.

S3.5 MDM2-Nutlin

The complex of mouse double minute 2 homolog (MDM2) (with truncated lid - "Short") with Pip2 was prepared by aligning PDB ID 4WT2 to PDB ID 4HG7. The protein structure was taken from 4WT2 from residue 17 (S) to the end (110, V), as this most closely matched the experimental construct. The ligand and crystallographic waters were taken from 4HG7, and subsequent preparation was performed as described in Section 3.1.

S4 Detailed Results for Non-Adaptive ABFE Calculations on Initial Test Systems

Table S1: Components of Non-Adaptive $\Delta G^o_{\mathrm{Bind}}$ for Initial Test Systems[a]

	Bound Restrain	Bound Discharge	Bound Vanish	Free Discharge	Free Vanish	Restraint Correction	Symmetry Correction	Exp. $\Delta G^o_{\mathrm{Bind}}$
T4L 0.2 ns	2.03 ± 0.15	0.27 ± 0.13	4.04 ± 0.73	2.07 ± 0.03	-7.14 ± 0.19	-7.08	-0.41	-5.19 ± 0.16
T4L 6 ns	1.97 ± 0.06	0.21 ± 0.05	3.10 ± 0.96	2.05 ± 0.01	-7.25 ± 0.05	-7.08	-0.41	-5.19 ± 0.16
T4L 30 ns	1.96 ± 0.02	0.18 ± 0.02	3.38 ± 0.81	2.05 ± 0.00	-7.24 ± 0.02	-7.08	-0.41	-5.19 ± 0.16
MIF 0.2 ns	1.70 ± 0.21	19.12 ± 2.56	2.21 ± 1.43	14.35 ± 0.13	-17.58 ± 0.27	-10.35	-0.65	-8.98 ± 0.28
MIF 6 ns	1.71 ± 0.07	18.04 ± 1.28	-3.51 ± 0.45	14.23 ± 0.02	-17.85 ± 0.05	-10.35	-0.65	-8.98 ± 0.28
MIF 30 ns	1.81 ± 0.10	17.68 ± 1.03	-2.98 ± 0.87	14.17 ± 0.11	-17.84 ± 0.03	-10.35	-0.65	-8.98 ± 0.28
MDM2-Nutlin 0.2 ns	2.64 ± 1.18	139.34 ± 1.25	-21.78 ± 4.02	140.59 ± 1.23	-48.12 ± 1.57	-9.85	0.00	-11.14 ± 0.27
MDM2-Nutlin 6 ns	2.33 ± 0.79	138.82 ± 1.16	-25.06 ± 1.88	140.17 ± 0.34	-49.49 ± 0.51	-9.85	0.00	-11.14 ± 0.27
MDM2-Nutlin 30 ns	2.33 ± 0.34	138.17 ± 0.89	-23.94 ± 1.06	140.16 ± 0.23	-50.39 ± 0.67	-9.85	0.00	-11.14 ± 0.27
MDM2-Pip2 0.2 ns	1.65 ± 0.09	54.28 ± 0.75	-6.88 ± 1.38	53.93 ± 0.36	-32.34 ± 1.26	-10.14	0.00	-9.11 ± 0.01
MDM2-Pip2 6 ns	1.67 ± 0.03	52.82 ± 0.38	-11.12 ± 0.71	53.54 ± 0.12	-33.81 ± 0.69	-10.14	0.00	-9.11 ± 0.01
MDM2-Pip2 30 ns	1.76 ± 0.17	52.74 ± 0.39	-11.06 ± 1.05	53.46 ± 0.11	-34.10 ± 0.21	-10.14	0.00	-9.11 ± 0.01
PDE2A 0.2 ns	1.74 ± 0.17	214.78 ± 0.85	4.20 ± 2.70	214.23 ± 0.35	-33.15 ± 1.53	-10.24	0.00	-14.35 ± 0.50
PDE2A 6 ns	1.84 ± 0.17	214.54 ± 0.54	-9.55 ± 1.70	213.49 ± 0.18	-34.16 ± 0.56	-10.24	0.00	-14.35 ± 0.50
PDE2A 30 ns	1.94 ± 0.14	214.22 ± 0.62	-9.53 ± 1.81	213.40 ± 0.12	-33.97 ± 0.34	-10.24	0.00	-14.35 ± 0.50

[a] All quantities in kcal mol^{-1}. Uncertainties stated as 95 % confidence intervals based on the variance of 5 replicate runs, assuming Gaussian distributions.

S5 Restraints Parameters and Discussion of Symmetry Corrections

Table S2: Parameters for Boresch restraints for initial test systems, as labelled in Figure 3 of Clark et al..[6] K refers to a force constant and 0 denotes an equilibrium value.

	T4L	MIF	MDM2-Pip2	PDE2A	MDM2-Nutlin
r1	1550	952	1307	4395	1330
r2	1530	950	1295	4393	1318
r3	1552	959	1309	4408	1332
l1	4	10	19	11	28
l2	3	13	14	10	6
l3	5	20	20	12	21
r_0 / Å	7.69	5.66	5.92	6.59	7.05
θ_{A0} / Å	1.30	2.14	1.80	1.71	1.32
θ_{B0} / Rad	1.48	1.48	1.28	1.27	1.43
ϕ_{A0} / Rad	2.56	1.84	-2.57	2.35	-2.78
ϕ_{B0} / Rad	2.94	3.09	1.05	0.00	1.21
ϕ_{C0} / Rad	1.41	0.22	-0.96	0.21	0.91
k_r / kcal mol^{-1} Å^{-2}	6.20	18.00	8.06	12.42	7.82
$k_{\theta\mathrm{A}}$ / kcal mol^{-1} Rad^{-2}	28.76	87.34	92.70	76.28	62.12
$k_{\theta\mathrm{B}}$ / kcal mol^{-1} Rad^{-2}	24.82	89.72	88.14	85.28	141.90
$k_{\phi\mathrm{A}}$ / kcal mol^{-1} Rad^{-2}	59.86	101.06	76.44	228.30	53.84
$k_{\phi\mathrm{B}}$ / kcal mol^{-1} Rad^{-2}	0.80	115.14	161.32	139.72	132.66
$k_{\phi\mathrm{C}}$ / kcal mol^{-1} Rad^{-2}	55.18	99.32	149.54	101.36	199.86

The symmetry corrections shown in Table S1 were selected as follows: for T4L, benzene was observed to rotate freely about its 6-fold axis of symmetry at $\lambda = 0$ during the restraining simulations, but was not observed to flip over. Therefore, we included a correction of $-k_{\mathrm{B}}T \ln 2$. For MIF, we included a correction of $-k_{\mathrm{B}}T \ln 3$ to account for the three-fold symmetry of the protein. No symmetry corrections were required for MDM2-Pip2, MDM2-Nutlin, or PDE2a.

S6 Estimated Free Energy Changes Against Sampling Times for Initial Non-Adaptive Runs

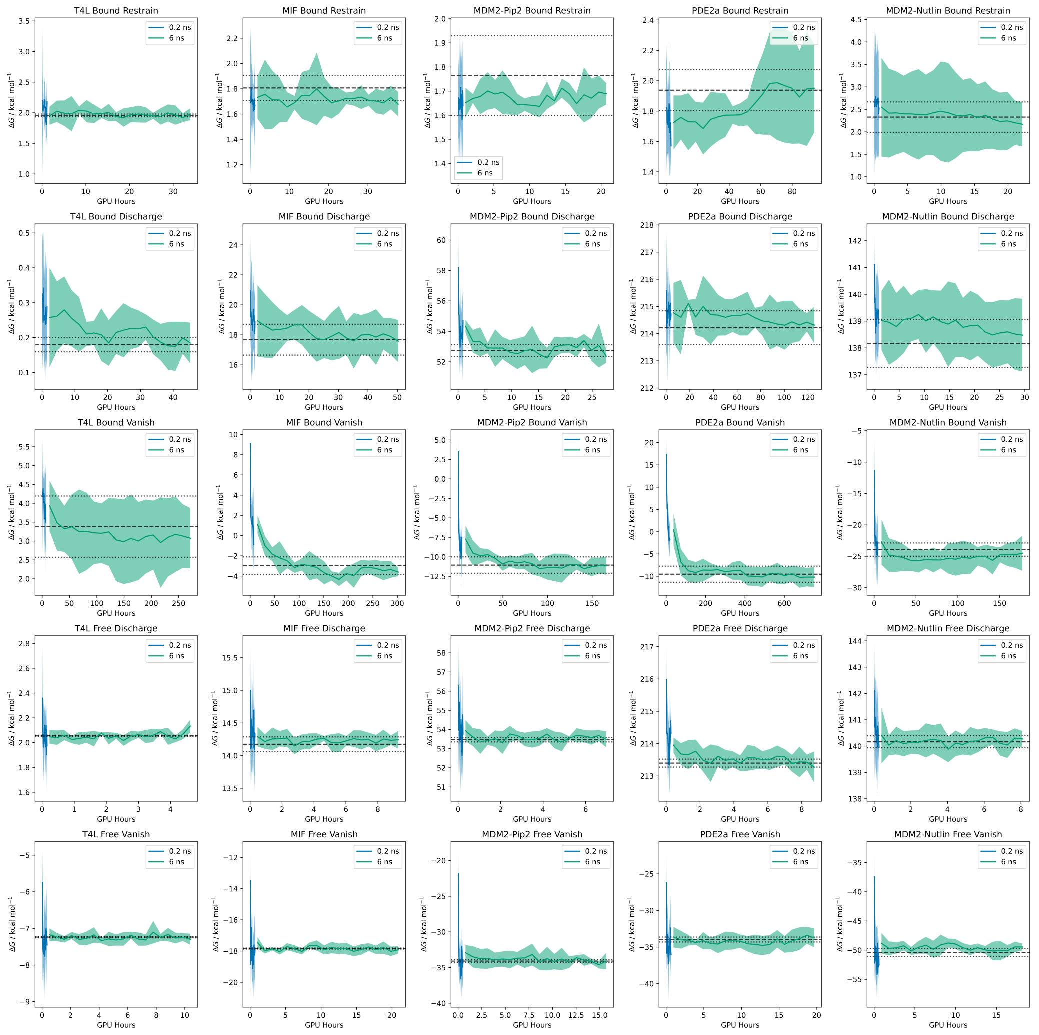

Figure S1: Estimated free energy changes against sampling times for all stages of all initial non-adaptive runs. Traces were calculated by dividing the data for each run into 20 blocks and performing MBAR individually on each block. No data were discarded to equilibration. Shaded areas show the 95 % t-based confidence intervals based on deviations between replicate runs. Results are shown for the 0.2 and 6 ns runs, and the final 30 ns result (discarding the initial 10 ns of each window to equilibration) is shown as a dotted line, with 95 % CI boundaries shown as sparser dotted lines.

S7 Summary of Kruskal-Wallis H-tests on Gradient Distributions

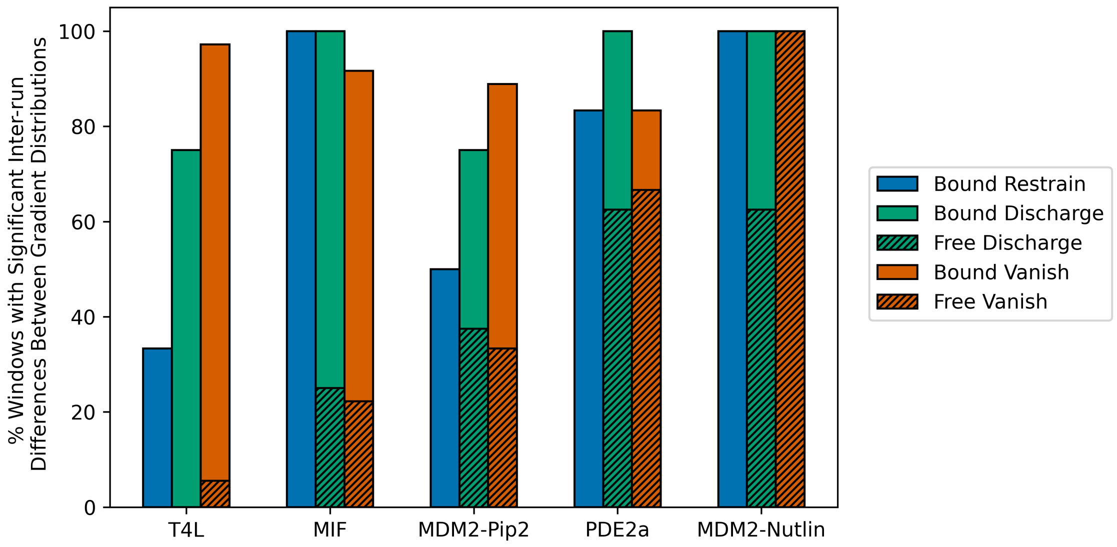

Figure S2: Percentage of λ-windows for each stage for which there is a significant difference between gradient distributions, as indicated by $p< 0.05$ from the Kruskal-Wallis H-test. This is always high for the bound discharge and bound vanish stages, and increases from around 0 to > 60 with ligand size for the free stages.

Figure S2 shows significant differences between gradient distributions between replicate runs for most λ-windows for all bound leg simulations. A strict criterion for convergence is that all repeat simulations should sample from the same distribution. By this criterion, none of the calculations are strictly converged, as discussed in the main text. To illustrate that this is likely also true for most literature ABFE studies, we reanalysed the results of Alibay et al..[10,11] The analysis (Figure S3 yielded similar results, confirming that most literature ABFE results are also likely unconverged by this strict criterion.

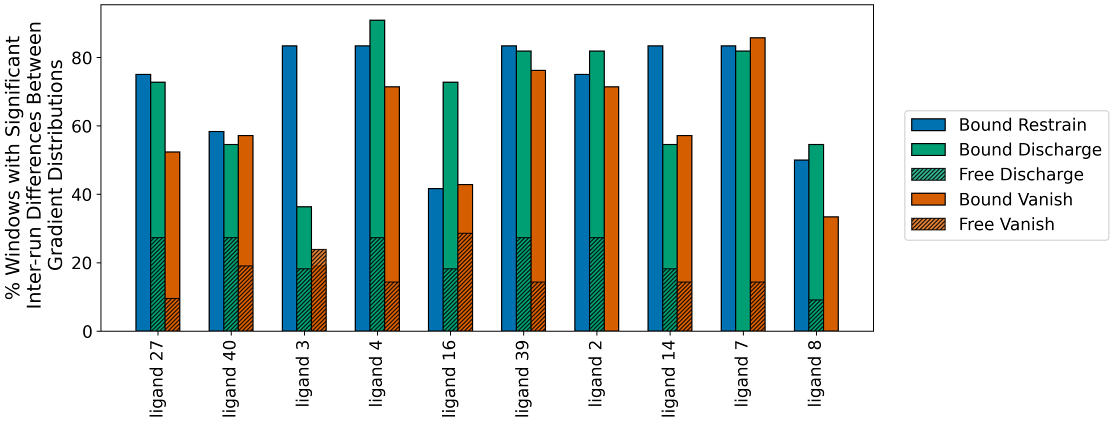

Figure S3: Percentage of λ-windows for each stage for which there is a significant difference between gradient distributions for Alibay et al.'s Cyclophilin D ABFE calculations.[10,11] Significant differences were indicated by $p < 0.05$ from the Kruskal-Wallis H-test.

S8 Standard Deviations of the Gradients and Time-Normalised Standard Errors of the Mean Gradients

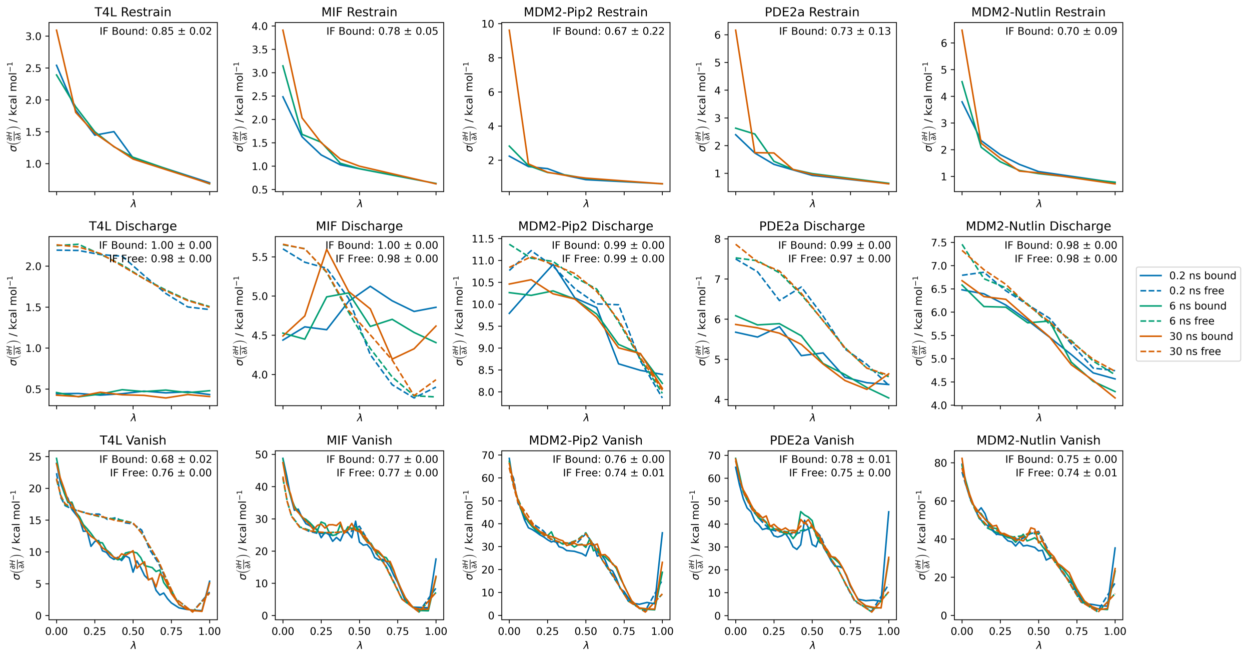

Figure S4: Standard deviation of the gradient against λ for initial non-adaptive runs. Improvement factor (IF) is calculated with respect to equally-spaced λ-windows.

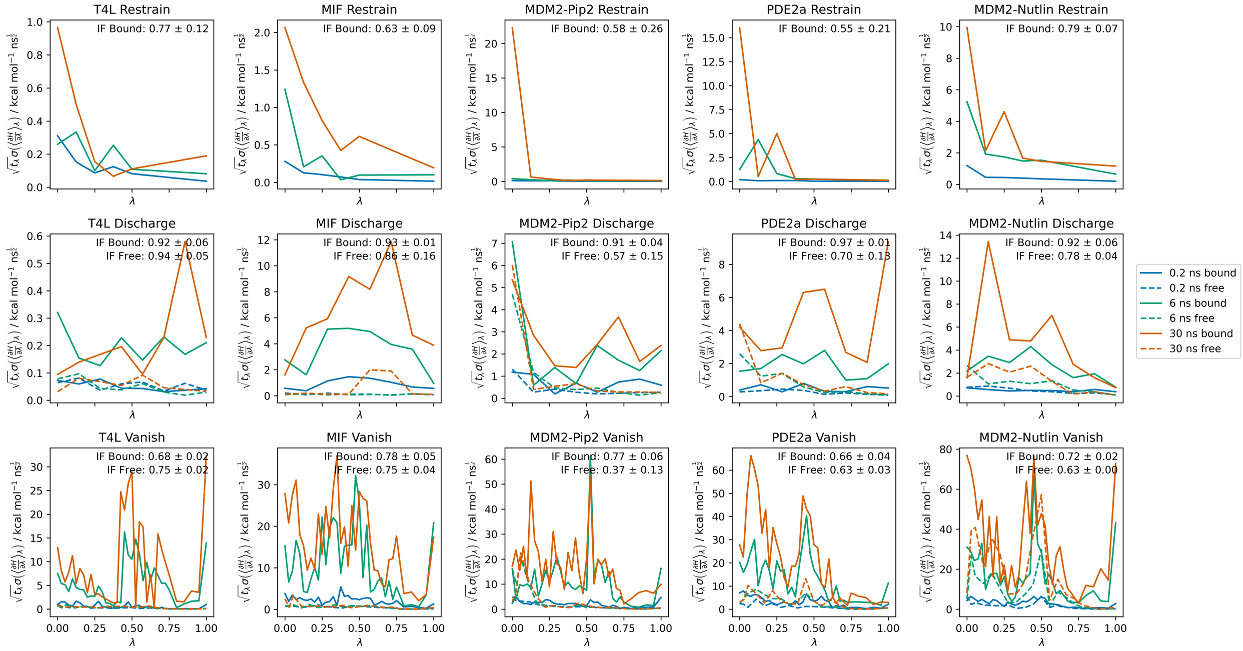

Figure S5: Time-normalised standard error of the mean gradient against λ for initial non-adaptive runs. Improvement factor (IF) is calculated with respect to equally-spaced λ-windows.

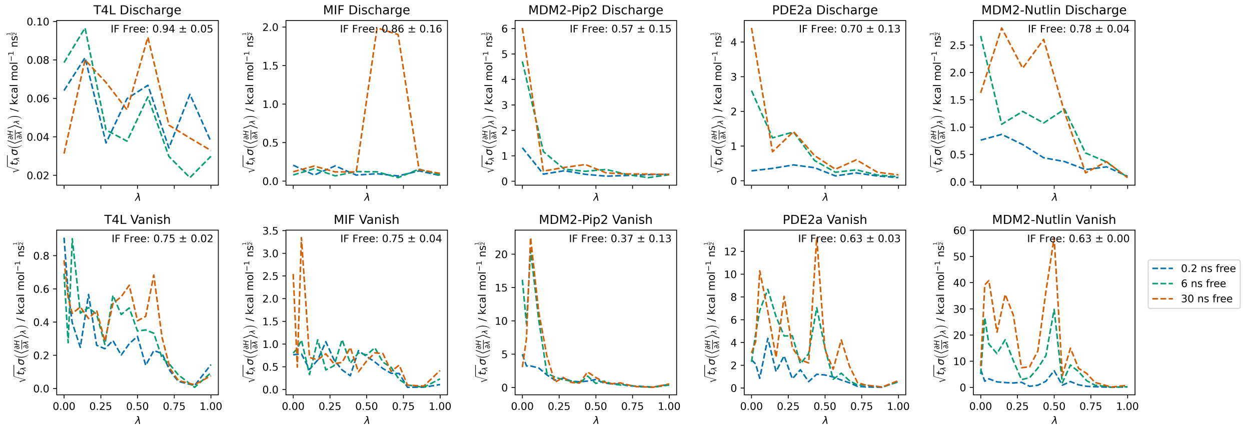

Figure S6: Time-normalised standard error of the mean gradient against λ for initial non-adaptive runs, showing only results for the free leg. Improvement factor (IF) is calculated with respect to equally-spaced λ-windows.

S9 Nutlin Conformations Observed During the Free Vanish Leg At and Above $\lambda = 0.5$

The time-normalised standard error of the mean gradients for the free vanish stage were high relative to the other ligands, and comparable to those for the bound legs. The peak around $\lambda = 0.5$ visible in Figure S6 is very likely due to different dominant conformations of Nutlin resulting in different amounts of overlap and differing gradients between runs. Below $\lambda = 0.5$, the intramolecular repulsive interactions are strong enough to avoid direct overlap as seen in b) of Figure S4. The window at $\lambda = 0.5$ is the first where this occurs, producing more negative gradients for runs 3 and 5 where conformation b) is dominant, and less negative gradients for runs 1, 2, and 4, where conformation a) is dominant.

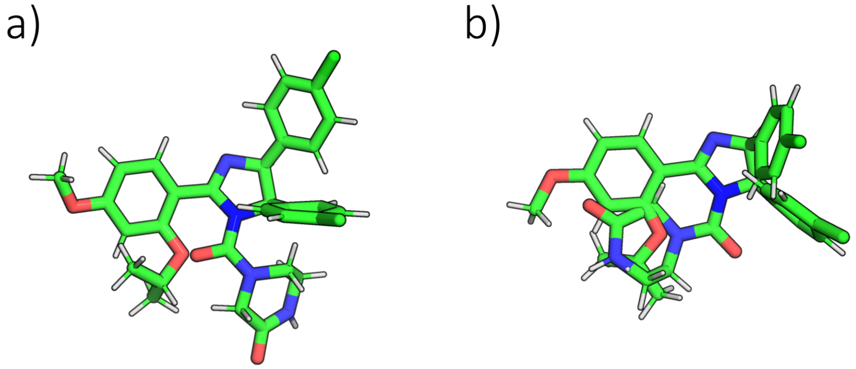

Figure S7: Examples of typical conformations observed for Nutlin during the free vanish stages a) with the piperazinone ring pointing away from the isopropoxy group, reducing atomic overlap and b) with the piperazinone ring pointing towards the isopropoxy group, resulting in substantial atomic overlap.

S10 Testing Automated Window Spacing with MIF

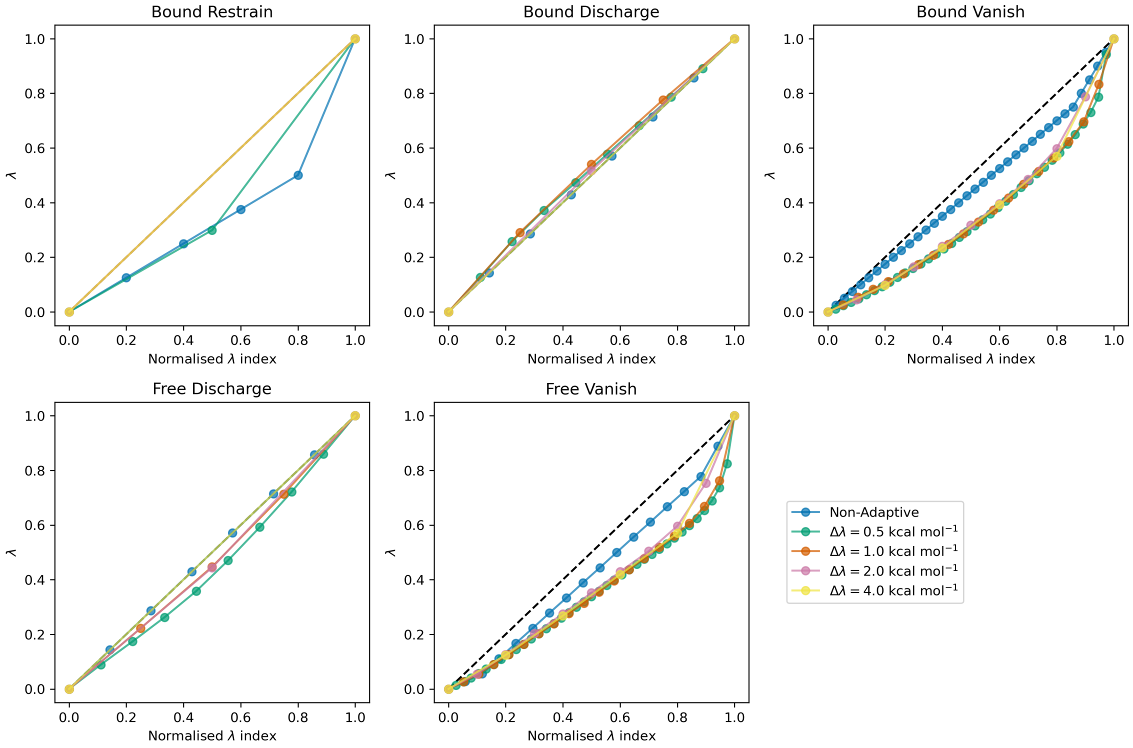

Figure S8: λ against normalised λ index ($\frac{\text{Index}}{\text{No.windows}}$) for all legs and stages for MIF using a manually-optimised λ schedule (non-adaptive), and using the automated method to space windows with thermodynamic speeds of 0.5, 1.0, 2.0, and 4.0 kcal mol^{-1}.

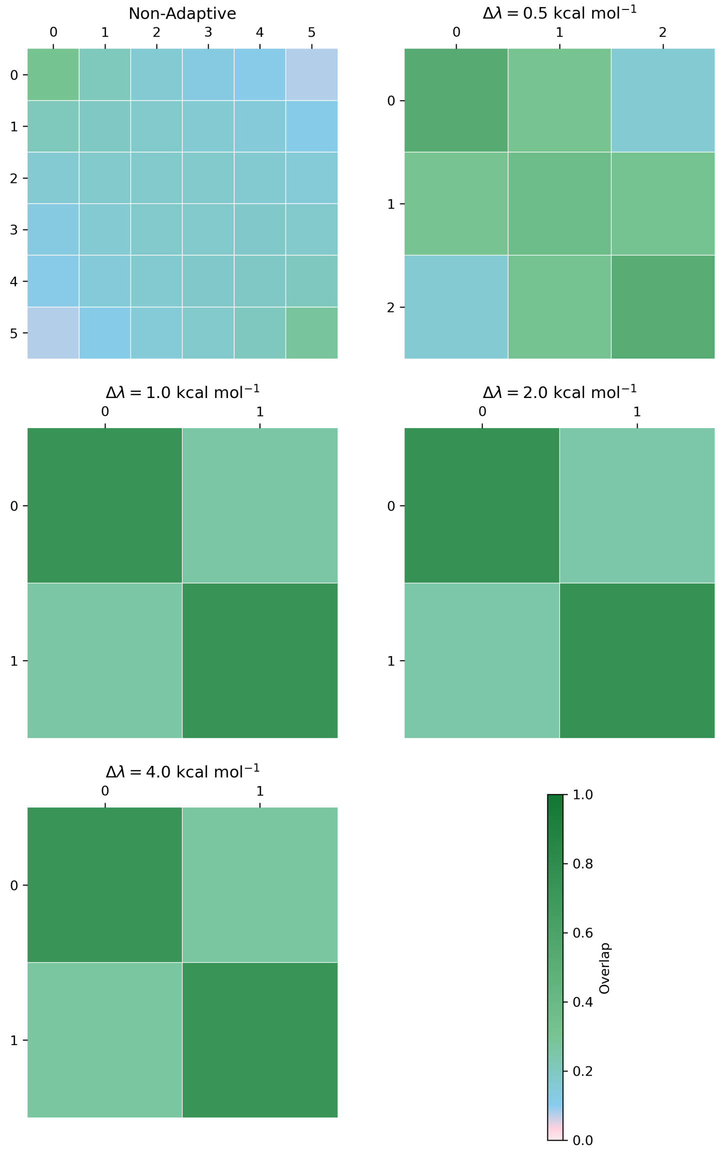

Figure S9: Overlap matrices for the bound restrain stage for MIF, using a manually-optimised λ schedule, and using the automated method to space windows with thermodynamic speeds of 0.5, 1.0, 2.0, and 4.0 kcal mol^{-1}.

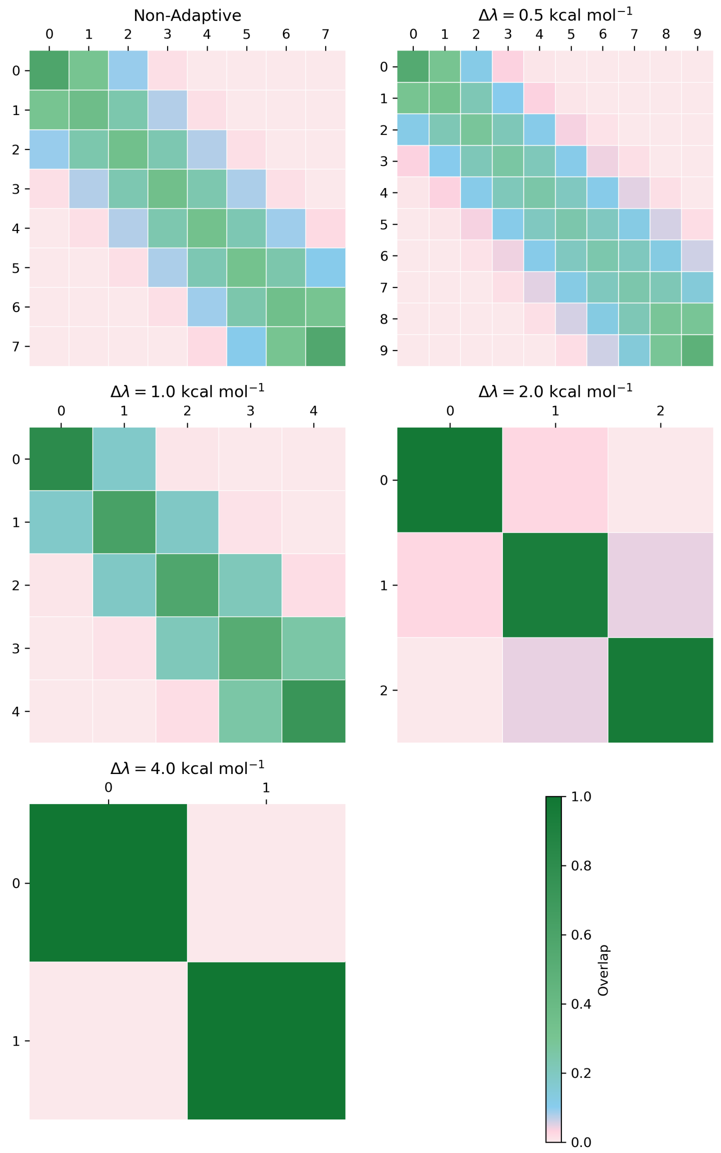

Figure S10: Overlap matrices for the bound discharge stage for MIF, using a manually-optimised λ schedule, and using the automated method to space windows with thermodynamic speeds of 0.5, 1.0, 2.0, and 4.0 kcal mol^{-1}.

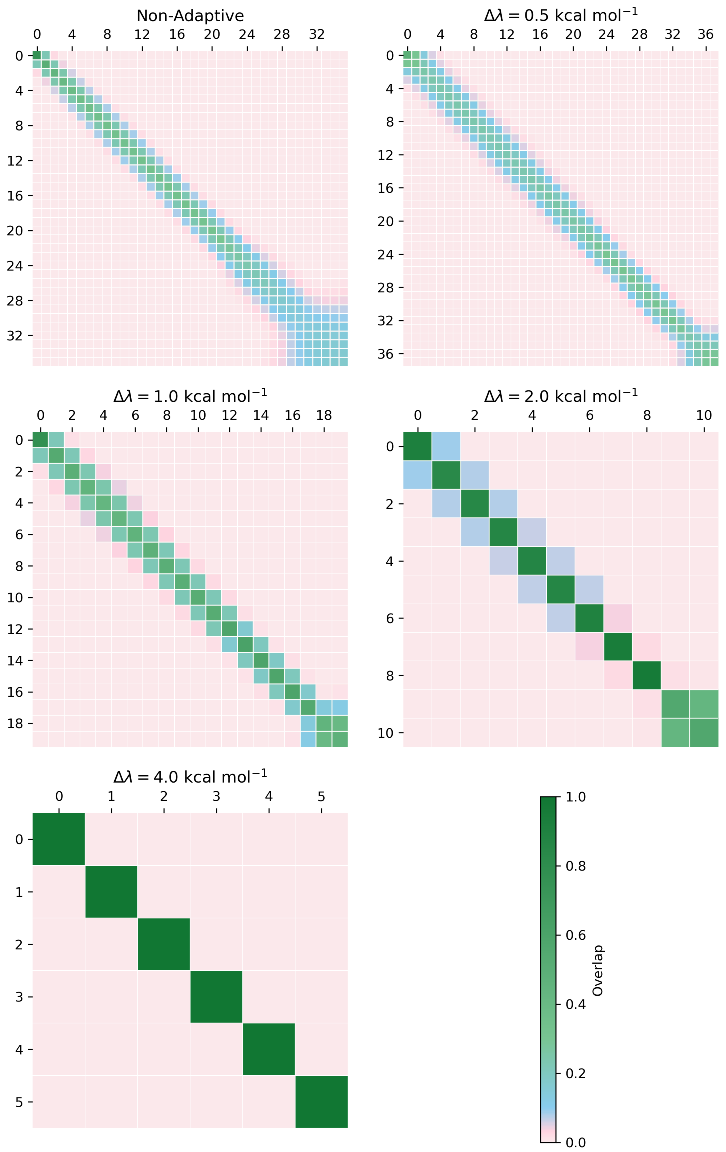

Figure S11: Overlap matrices for the bound vanish stage for MIF, using a manually-optimised λ schedule, and using the automated method to space windows with thermodynamic speeds of 0.5, 1.0, 2.0, and 4.0 kcal mol^{-1}.

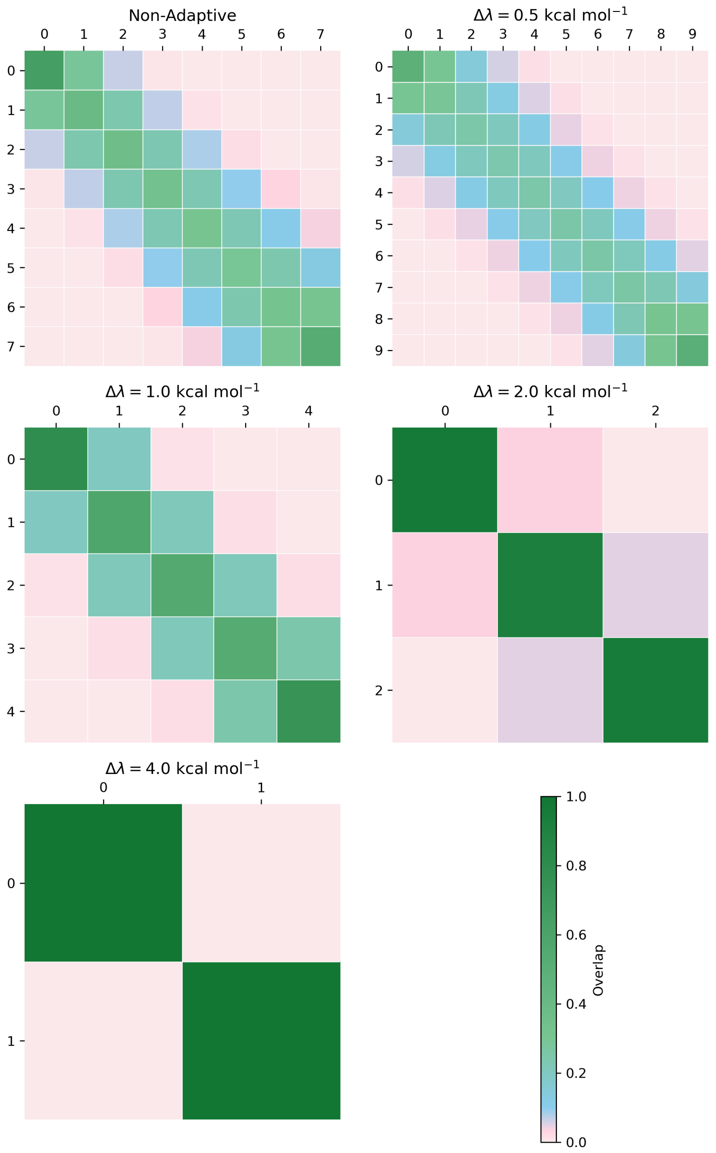

Figure S12: Overlap matrices for the free discharge stage for MIF, using a manually-optimised λ schedule, and using the automated method to space windows with thermodynamic speeds of 0.5, 1.0, 2.0, and 4.0 kcal mol^{-1}.

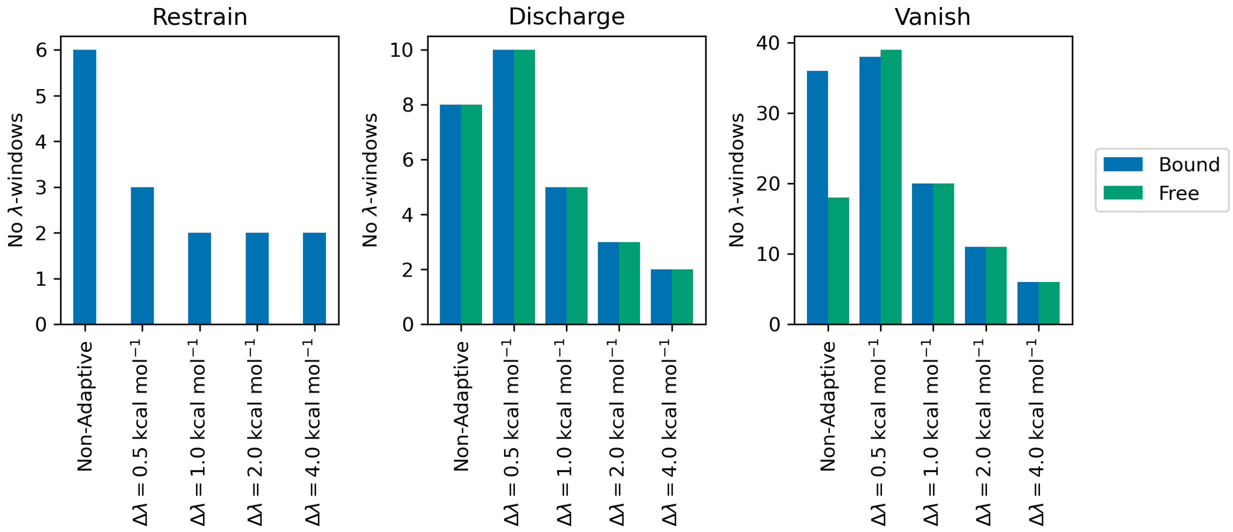

Figure S13: Number of windows selected for each stage for MIF. Non-adaptive shows the default number of windows, which were previously manually optimised for MIF/MIF180.[6] All other schedules were generated with the automated procedure using thermodynamic speeds of 0.5, 1.0, 2.0, and 4.0 kcal mol^{-1}.

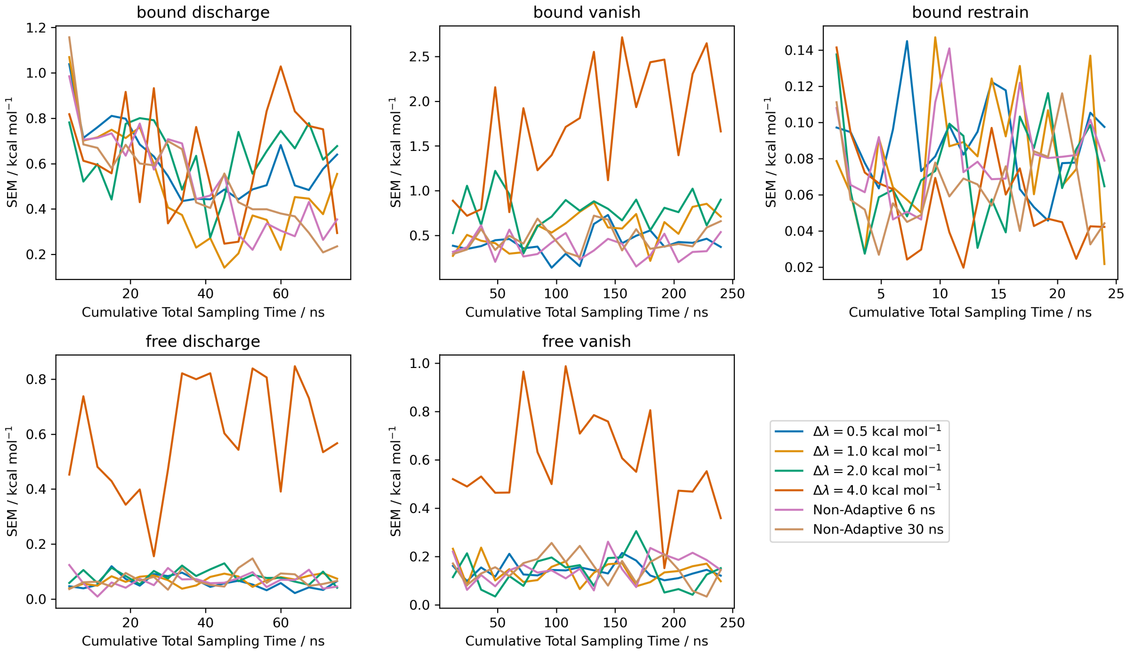

Figure S14: Non- cumulative inter-run standard errors of the mean of estimated ΔG against sampling time for all stages with different λ schedules. Equal simulation times were allocated to each window for within each schedule. Data were split into 20 equal blocks and MBAR was run on each block. By non-cumulative, we mean that the data for each block were analysed independently, so that the overall error is not expected to decrease with $\frac{1}{\sqrt{\text{SamplingTime}}}$. The data for truncated before analysis so that the per-stage computational costs were equal to that of the cheapest stage.

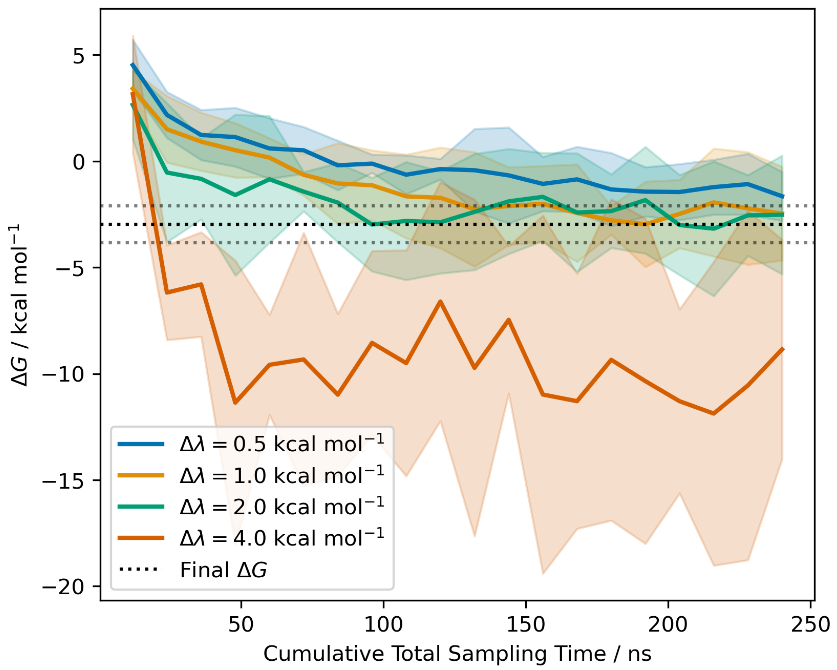

Figure S15: Longer-time equilibration of the MIF bound vanish stage against total sampling time with varying λ-window spacing. Individual replicates are shown with dashed lines, the mean is shown as a solid line, and shaded regions indicate 95 % t-based confidence intervals. Wider spacing leads to accelerated equilibration towards the 30 ns result (black-dotted line with 95 % CI shown as sparse dotted lines).

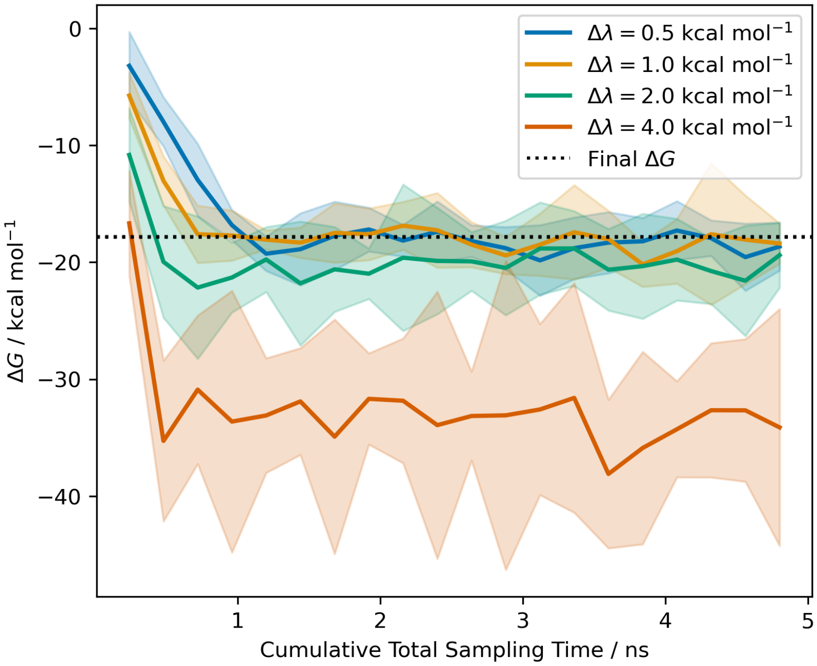

Figure S16: Short-time equilibration of the MIF free vanish stage against total sampling time with varying λ-window spacing. Individual replicates are shown with dashed lines, the mean is shown as a solid line, and shaded regions indicate 95 % t-based confidence intervals. Wider spacing leads to accelerated equilibration towards the 30 ns result (black-dotted line with 95 % CI shown as sparse dotted lines).

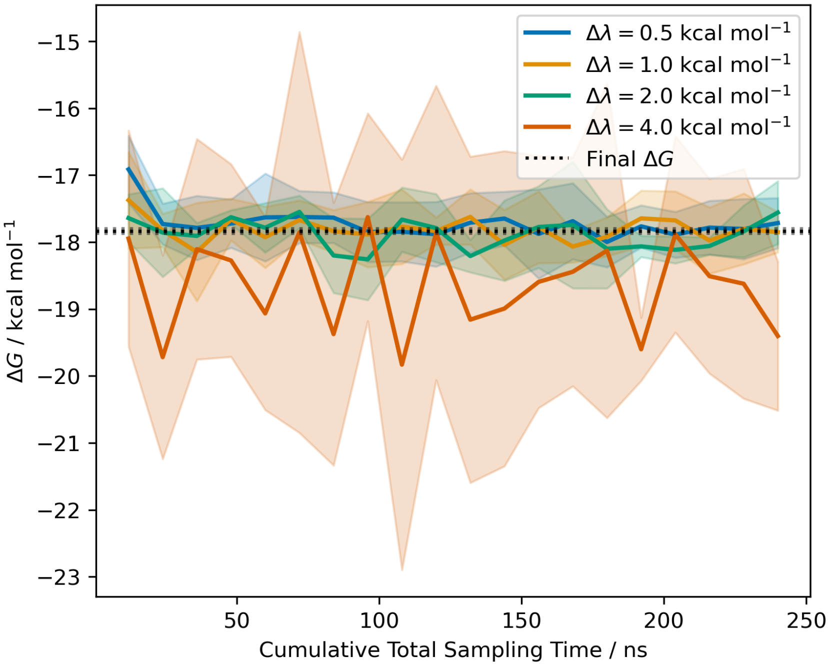

Figure S17: Longer-time equilibration of the MIF free vanish stage against total sampling time with varying λ-window spacing. Individual replicates are shown with dashed lines, the mean is shown as a solid line, and shaded regions indicate 95 % t-based confidence intervals. Wider spacing leads to accelerated equilibration towards the 30 ns result (black-dotted line with 95 % CI shown as sparse dotted lines).

S11 Free Energies for Variation of Allocated Simulation Time with Adaptive Parameters for MIF

Table S3: Components of Free Energies for Variation of Allocated Simulation Time with Simulation Parameters for MIF Experiments [a]

	Bound Restrain	Bound Discharge	Bound Vanish	Free Discharge	Free Vanish	Restraint Correction	Symmetry Correction	Exp. $\Delta G^o_{\mathrm{Bind}}$
r0.005, s1, n5, repeat 1	1.73 ± 0.15	18.17 ± 0.82	-2.13 ± 1.25	14.25 ± 0.11	-17.97 ± 0.20	-10.35	0.65	-8.98 ± 0.28
r0.005, s1, n5, repeat 2	1.72 ± 0.13	17.98 ± 1.04	-2.19 ± 0.83	14.21 ± 0.06	-17.77 ± 0.05	-10.35	0.65	-8.98 ± 0.28
r0.005, s1, n5, repeat 3	1.73 ± 0.15	18.61 ± 1.14	-2.79 ± 1.43	14.25 ± 0.06	-17.68 ± 0.37	-10.35	0.65	-8.98 ± 0.28
r0.005, sOrig., n5	1.71 ± 0.16	17.70 ± 1.06	-1.06 ± 0.64	14.16 ± 0.11	-17.62 ± 0.10	-10.35	0.65	-8.98 ± 0.28
r0.001, s1, n5	1.79 ± 0.15	18.28 ± 0.74	-2.56 ± 0.42	14.24 ± 0.04	-17.79 ± 0.08	-10.35	0.65	-8.98 ± 0.28
r0.005, s1, n10	1.73 ± 0.11	18.07 ± 0.70	-1.40 ± 0.89	14.24 ± 0.11	-17.80 ± 0.19	-10.35	0.65	-8.98 ± 0.28
r0.005, s0.5, n5	1.69 ± 0.15	18.41 ± 1.40	-1.91 ± 0.43	14.20 ± 0.12	-17.75 ± 0.20	-10.35	0.65	-8.98 ± 0.28
r0.005, s2, n5, repeat 1	1.70 ± 0.10	17.74 ± 1.35	-3.53 ± 0.94	14.35 ± 0.13	-17.66 ± 0.08	-10.35	0.65	-8.98 ± 0.28
r0.005, s2, n5, repeat 2	1.80 ± 0.21	17.74 ± 1.20	-2.08 ± 0.91	14.31 ± 0.15	-17.69 ± 0.44	-10.35	0.65	-8.98 ± 0.28
r0.005, s4, n5	1.71 ± 0.15	18.14 ± 1.02	-3.61 ± 1.59	14.78 ± 0.59	-17.70 ± 0.44	-10.35	0.65	-8.98 ± 0.28

[a] All quantities in kcal mol^{-1}. Uncertainties stated as 95 % confidence intervals based on the variance of 5 replicate runs, assuming Gaussian distributions. In the protocol names, r denotes the run time constant ($\mathrm{kcal}^2\ \mathrm{mol}^{-2}\ \mathrm{ns}^{-1}$), s denotes the thermodynamic speed (kcal mol^{-1}) used to determine the λ-window spacing, and n denotes the number of replicates in each ensemble run. sOrig denotes the use of the default λ-spacing.

S12 Standard Error of the Mean Free Energy Change for the Pip2 Free Stage with Adaptive and Non-Adaptive Protocols

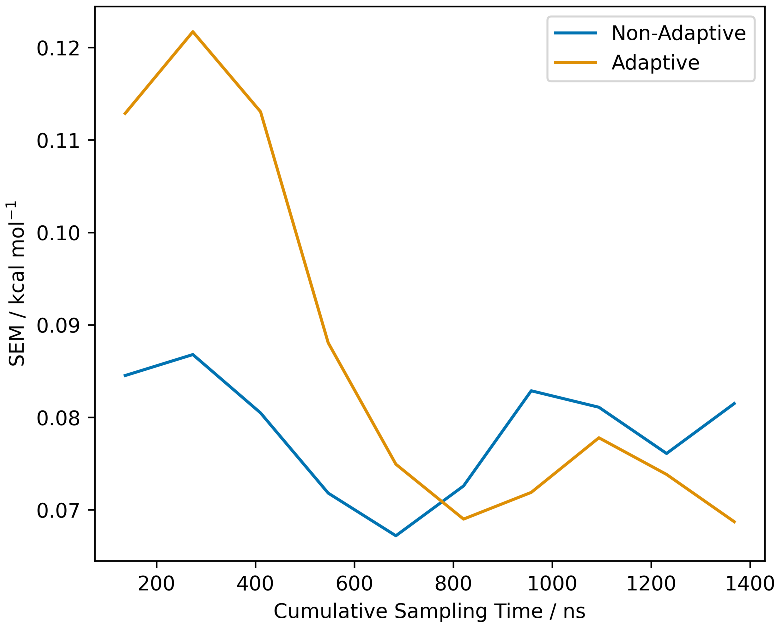

Figure S18: Standard error of the mean free energy change for the free vanish stage of Pip2. Data were split into 10 blocks prior to analysis with MBAR, and the uncertainties only account for the data in the current block (and are therefore not expected to decrease with $\sqrt{N_{\text{Blocks}}}$).

S13 Effect of the Adaptive Allocation of Sampling Time to the Bound Vanish Stage of T4L/Benzene

During the bound vanish stage of the non-adaptive 30 ns T4L run, the Gelman-Rubin $\hat{R}$ clearly identified convergence issues above $\lambda = 0.4$, and in particular close to $\lambda = 1$ (Figure S19). This was also reflected by the divergence of the potential of mean force with respect to λ in this region (Figure S20).

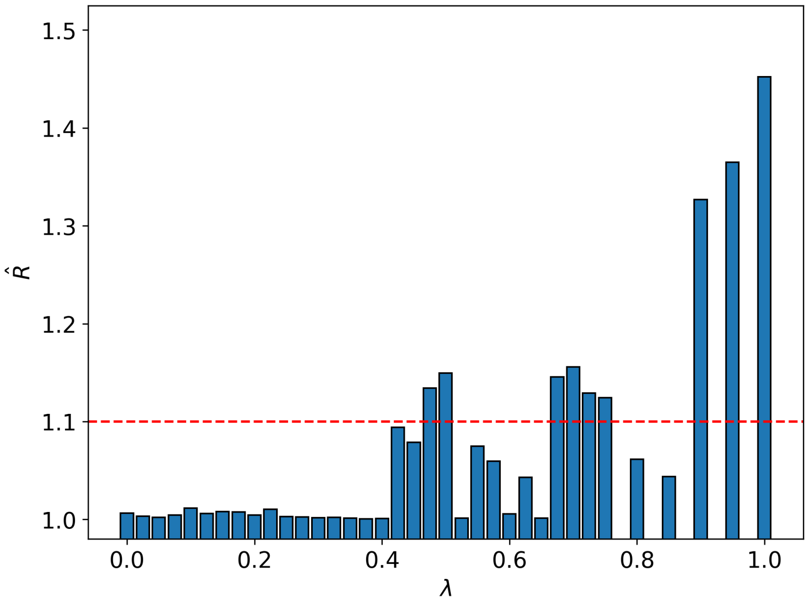

Figure S19: Gelman-Rubin $\hat{R}$ against λ for the bound vanish stage of the 30 ns T4L non-adapitve run. $\hat{R} > 1.1$ is taken to indicate convergence issues. $\hat{R}$ was computed with ArviZ 0.15.1.[12]

This was found to be due to the occasional entry of water to the binding site for some replicates above $\lambda = 0.4$. To check if we could remedy this using the adaptive sampling time algorithm, we performed adaptive and non-adaptive runs using 20 replicates and the same λ schedules (speed = 1.0 kcal mol^{-1}). The non-adaptive run was run for 20 ns per window, and the runtime constant of the adaptive run was tweaked until comparable total simulation time was achieved. As expected, the adaptive algorithm concentrated sampling time around $\lambda = 1$ (Figure S21). However, the plots of free energy estimates against simulation time for

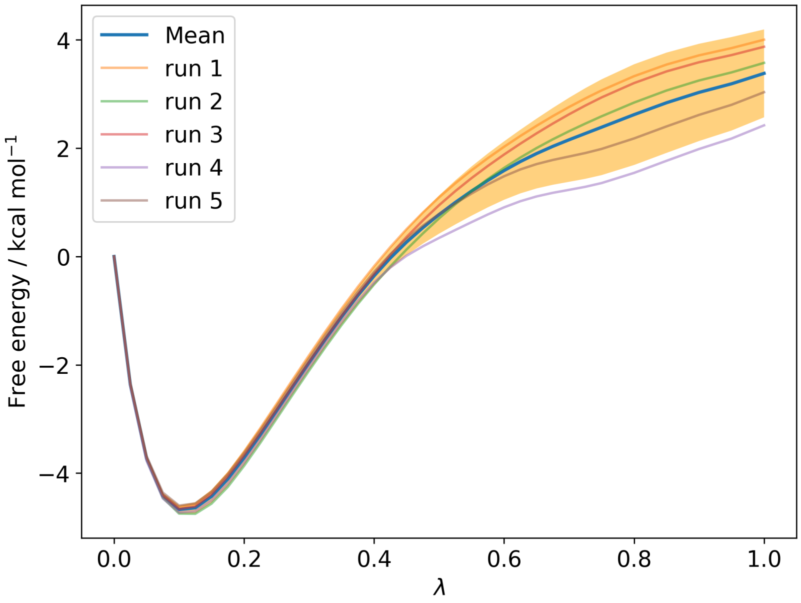

Figure S20: MBAR-derived potential of mean force against λ for the bound vanish stage of the 30 ns T4L non-adapitve run.

the adaptive and non-adaptive protocols appear very similar (Figure S22) , and there was no evidence for a significant difference in variance at 95 % confidence (Levene test, p=0.92). In addition, Figure S23 shows that the water occupancy of the binding site remained very variable between replicates and appeared similar between the adaptive and non-adaptive protocols (water occupancy was assessed by the mean number of waters with 6 Å of either of two atoms on opposite sides of the benzene rings). This suggests that the adaptive sampling algorithm is ineffective here because the timescales of water entry and exit from the binding site are beyond what can reasonably be allocated by the adaptive algorithm.

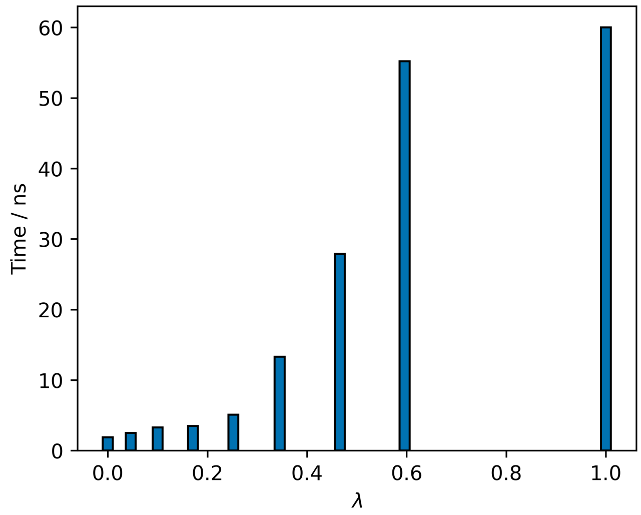

Figure S21: Allocation of sampling time against λ for the T4L bound vanish leg.

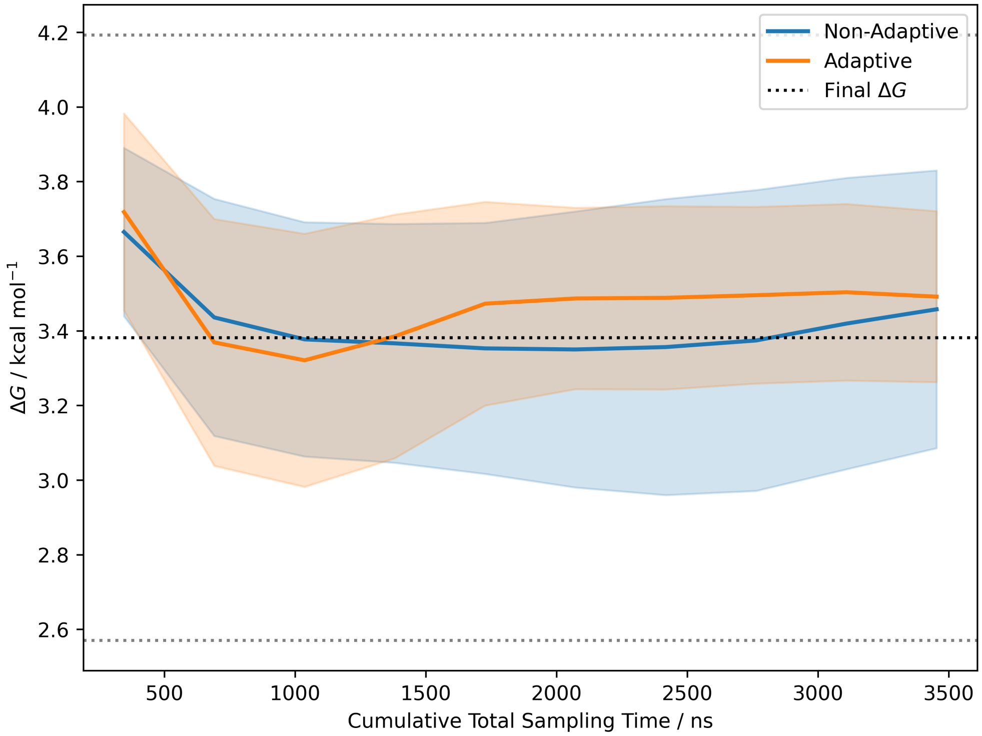

Figure S22: Estimated free energy change against simulation time for the T4L bound vanish stage with adaptive and non-adaptive protocols. The 30 ns result is shown by a black dotted line with sparser dotted lines showing 95 % CI. Data was split into 10 blocks before analysis with MBAR. Shaded areas show 95 % t-based CIs. The adaptive and non-adaptive runs use the same λ-schedule.

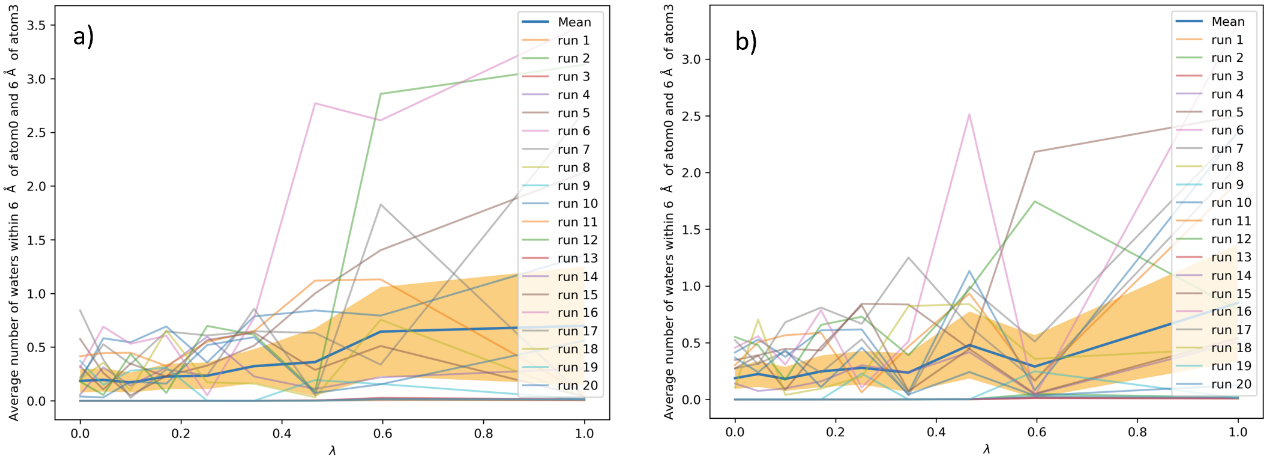

Figure S23: Average number of waters in the binding site for a) the non-adaptive and b) the adaptive protocol. Shaded area shows the 95 % t-based CI, although the assumption of Gaussian distributions is clearly unreasonable here. Atoms 0 and 3 are on opposite sides of the benzene ring.

S14 Selection of Equilibration Times for All Stages of Initial Test Systems

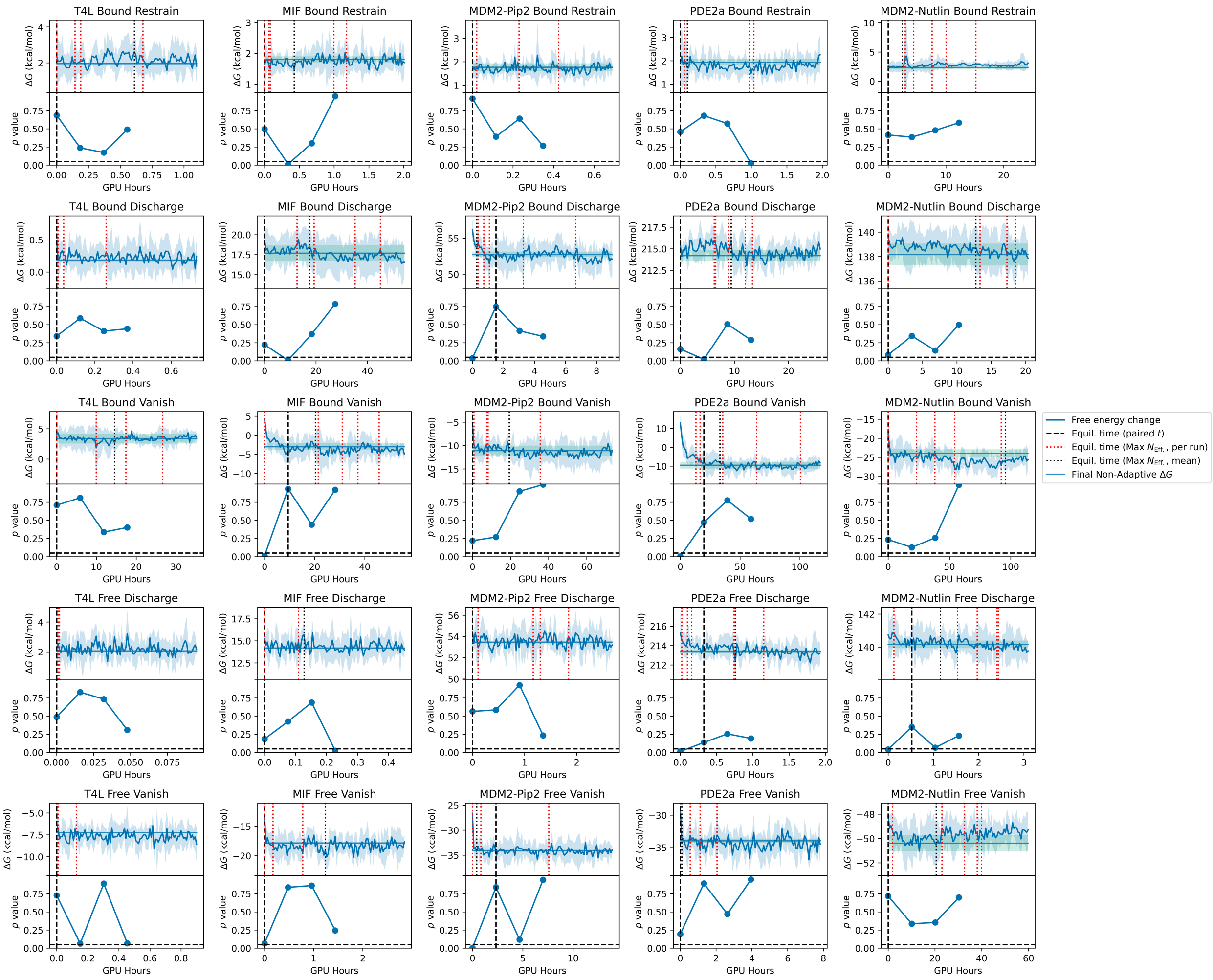

Figure S24: Selection of equilibration times for all stages for initial test systems by the paired t-test method and Chodera's method (applied to both the mean trace and individual replicates).[13] The upper windows show traces of ΔG obtained by dividing the data up into 100 equal blocks and running MBAR on each. Shaded areas indicate 95 % inter-run t-based confidence intervals. Final Non-Adaptive ΔG taken from the non-adaptive 30 ns runs. Lower windows show the p-values obtained by truncating the data up to the time shown and performing paired t-tests on the first 10 % and last 50 % of the remaining data. The first p-value > 0.05 is used as a heuristic to indicate equilibration.

S15 Comparison of Absolute Differences in Final Free Energy Estimates Compared to Long-Time Result Using Different Equilibration Methods

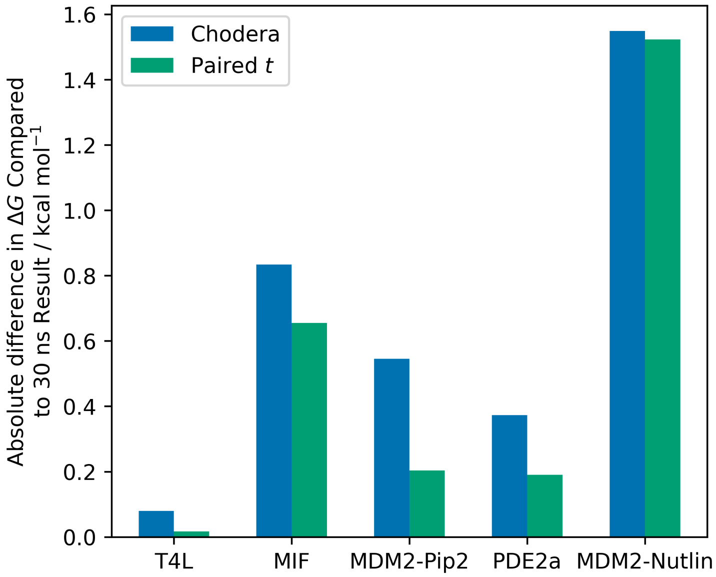

Figure S25: Comparison of the absolute error between bound vanish ΔG estimates using different equilibration methods, and the long-time non-adaptive results for these stages (from the 30 ns runs). The absolute errors are always smaller for the paired t method compared to Chodera's method (run on the mean trace). However, there is no evidence for a significant difference at 95 % based on the Wilcoxon signed-rank test ($p = 0.06$).

S16 Detailed Results for "Optimised" Adaptive ABFE Calculations on Initial Test Systems

Table S4: Components of Non-Adaptive $\Delta G^{o}_{\text{Bind}}$ for Initial Test Systems[a]

	Bound Restrain	Bound Discharge	Bound Vanish	Free Discharge	Free Vanish	Restraint Correction	Symmetry Correction	Exp. $\Delta G^{o}_{\text{Bind}}$
T4L adaptive	1.97 ± 0.06	0.23 ± 0.14	3.64 ± 0.71	2.11 ± 0.08	-7.22 ± 0.15	-7.08	-0.41	-5.19 ± 0.16
T4L non-adaptive	1.96 ± 0.02	0.18 ± 0.02	3.38 ± 0.81	2.05 ± 0.00	-7.24 ± 0.02	-7.08	-0.41	-5.19 ± 0.16
MIF adaptive	1.71 ± 0.15	17.71 ± 1.61	-2.70 ± 1.16	14.21 ± 0.14	-17.72 ± 0.13	-10.35	-0.65	-8.98 ± 0.28
MIF non-adaptive	1.81 ± 0.10	17.68 ± 1.03	-2.98 ± 0.87	14.17 ± 0.11	-17.84 ± 0.03	-10.35	-0.65	-8.98 ± 0.28
MDM2-Pip2 adaptive	1.67 ± 0.00	52.73 ± 0.68	-10.55 ± 1.18	53.50 ± 0.29	-33.78 ± 0.74	-10.14	0.00	-9.11 ± 0.01
MDM2-Pip2 non-adaptive	1.76 ± 0.17	52.74 ± 0.39	-11.06 ± 1.05	53.46 ± 0.11	-34.10 ± 0.21	-10.14	0.00	-9.11 ± 0.01
PDE2A adaptive	1.73 ± 0.15	214.69 ± 0.70	-8.22 ± 1.82	213.47 ± 0.23	-33.87 ± 0.27	-10.24	0.00	-14.35 ± 0.82
PDE2A non-adaptive	1.94 ± 0.14	214.22 ± 0.62	-9.53 ± 1.81	213.40 ± 0.12	-33.97 ± 0.34	-10.24	0.00	-14.35 ± 0.82
MDM2-Nutlin adaptive	2.61 ± 0.52	138.62 ± 1.17	-24.63 ± 0.84	140.16 ± 0.35	-49.55 ± 0.51	-9.85	0.00	-11.14 ± 0.27
MDM2-Nutlin non-adaptive	2.33 ± 0.34	138.17 ± 0.89	-23.94 ± 1.06	140.16 ± 0.23	-50.39 ± 0.67	-9.85	0.00	-11.14 ± 0.27

[a] All quantities in kcal mol^{-1}. Uncertainties stated as 95 % confidence intervals based on the variance of 5 replicate runs, assuming Gaussian distributions. "adaptive" refers to the "optimised" adaptive protocol, while "non-adaptive" refers to the the 30 ns non-adaptive protocol.

S17 Selection of Windows for Initial Test Systems with "Optimised" Adaptive Protocol

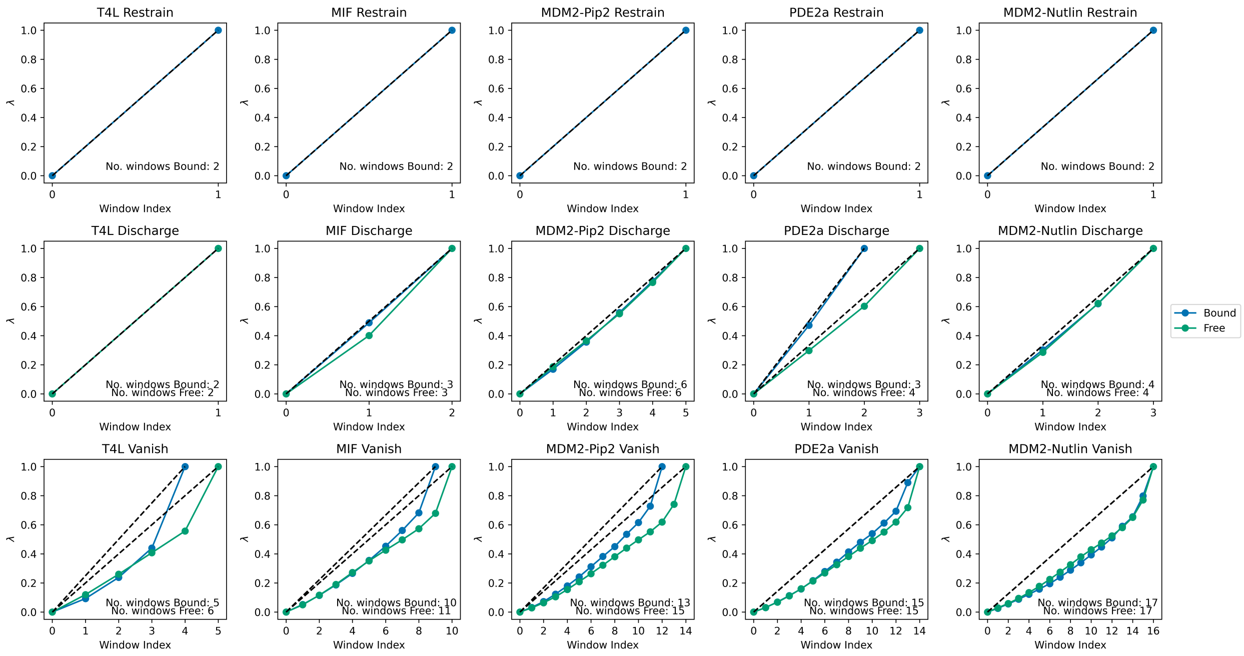

Figure S26: λ values selected for each of the initial test systems using a thermodynamic speed of 2 kcal mol^{-1}.

S18 Detailed Breakdown of Sampling Time Allocation for Initial Test Systems with "Optimised" Non-Adaptive Protocol

Note that the total allocation of simulation time depends not only on the inter-run error, but also the relative computational cost of the leg for the given system (Figure S27). If the statistical inefficiencies were the same for all windows, the allocated sampling times (Figures S28 and S29) should be a function only of the number of windows allocated. In this case, the sampling time per window would be expected to be the same for windows of the same computational cost, and hence large differences in sampling time per window (Figure S30) can highlight stages containing windows with particularly high statistical inefficiencies.

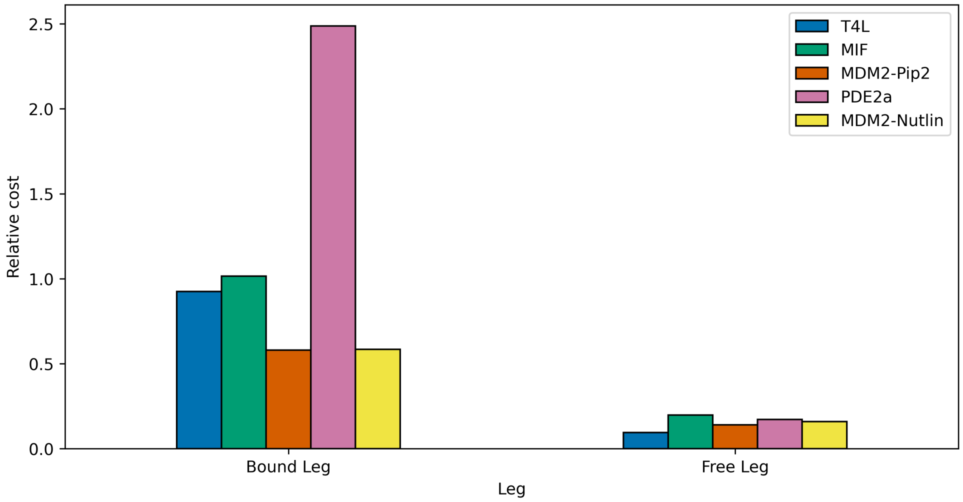

Figure S27: Relative computational costs of each leg for the initial test systems, calculated relative to the MIF180 bound leg. Calculated based on the average time taken to run a ns of simulation on a single GPU. The absolute cost of the MIF180 bound leg was 0.21 GPU hours / ns. All computational costs were assessed running on NVIDIA GeForce RTX2080 SUPER GPUs.

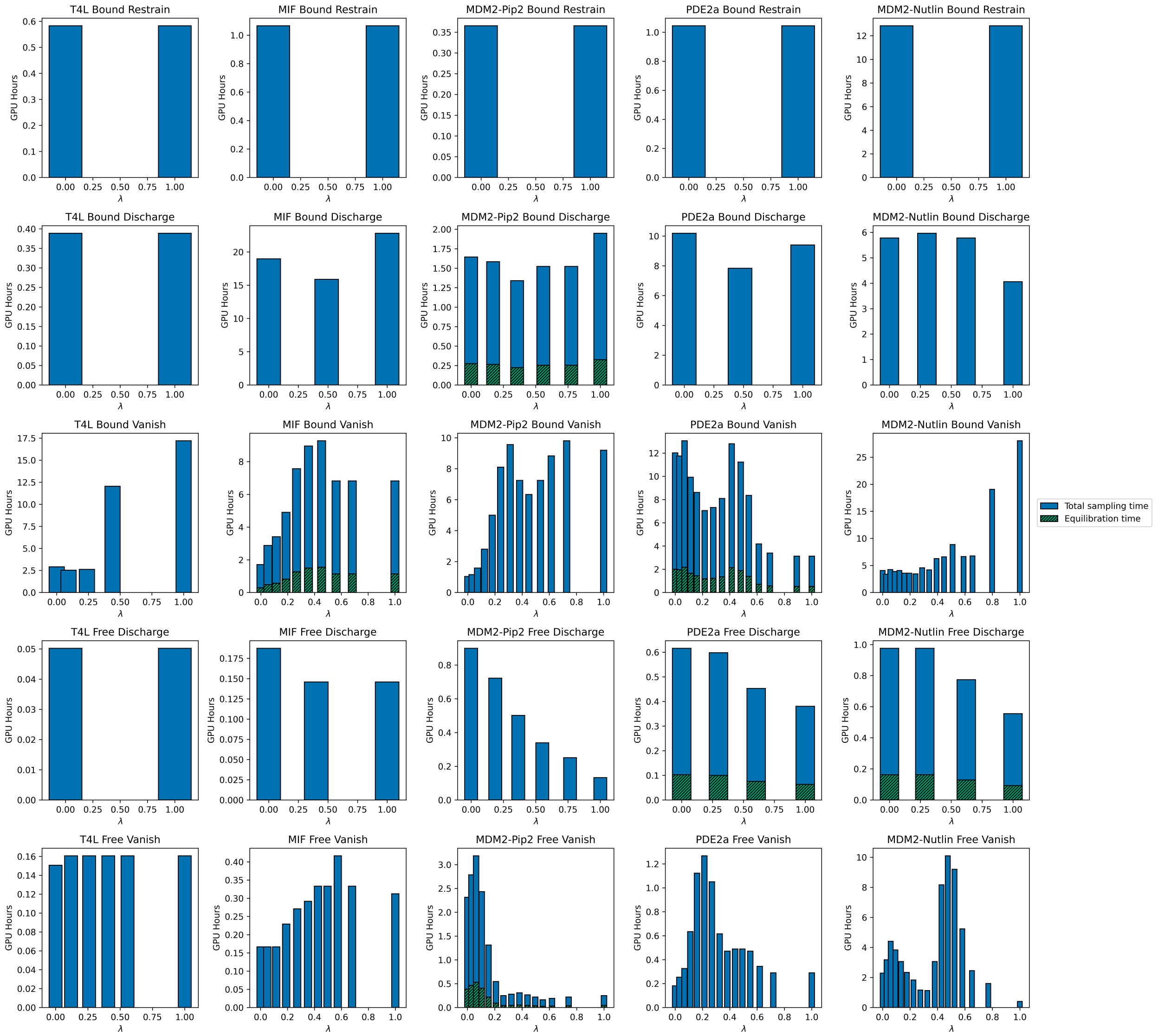

Figure S28: Per-window breakdown of sampling times allocated to the initial test systems with the “Optimised” adaptive protocol.

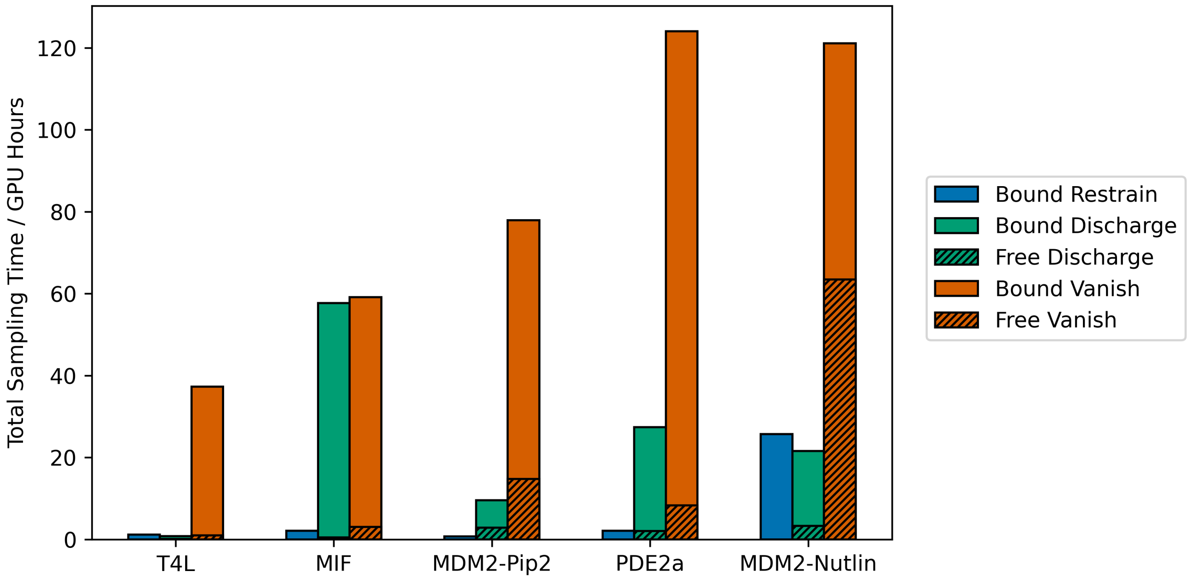

Figure S29: Per-stage breakdown of sampling times allocated to the initial test systems with the "Optimised" adaptive protocol.

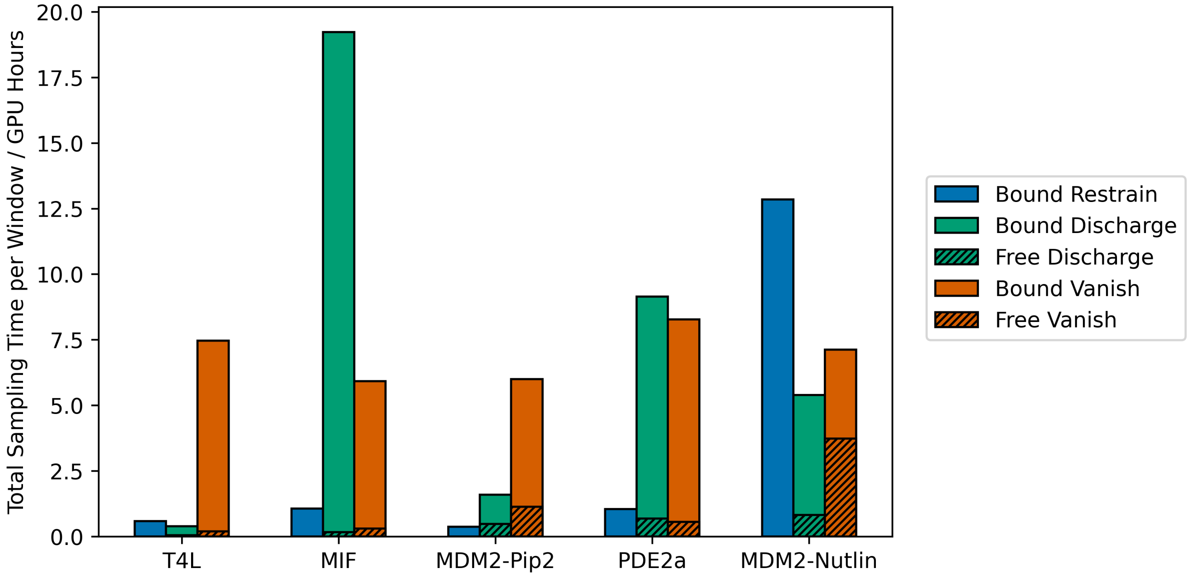

Figure S30: Per-stage breakdown of the per-window sampling times allocated to the initial test systems with the "Optimised" adaptive protocol.

S19 Total Allocated GPU Times for Adaptive and Non-Adaptive Protocols on Initial Systems

Table S5: Total Allocated GPU Times for Adaptive and Non-Adaptive Protocols on Initial Systems[a]

	T4L	MIF	MDM2-Pip2	PDE2a	MDM2-Nutlin	Total
Adaptive	40	122	106	164	235	668
0.2 ns	10	12	7	27	7	63
6 ns	377	429	250	1000	255	2311
30 ns	1536	1762	1029	4059	1053	9438

[a] All sampling times in GPU hours.

S20 Free Energy Estimates Against Sampling Time for Initial Test Systems with Adaptive and Non-Adaptive Protocols

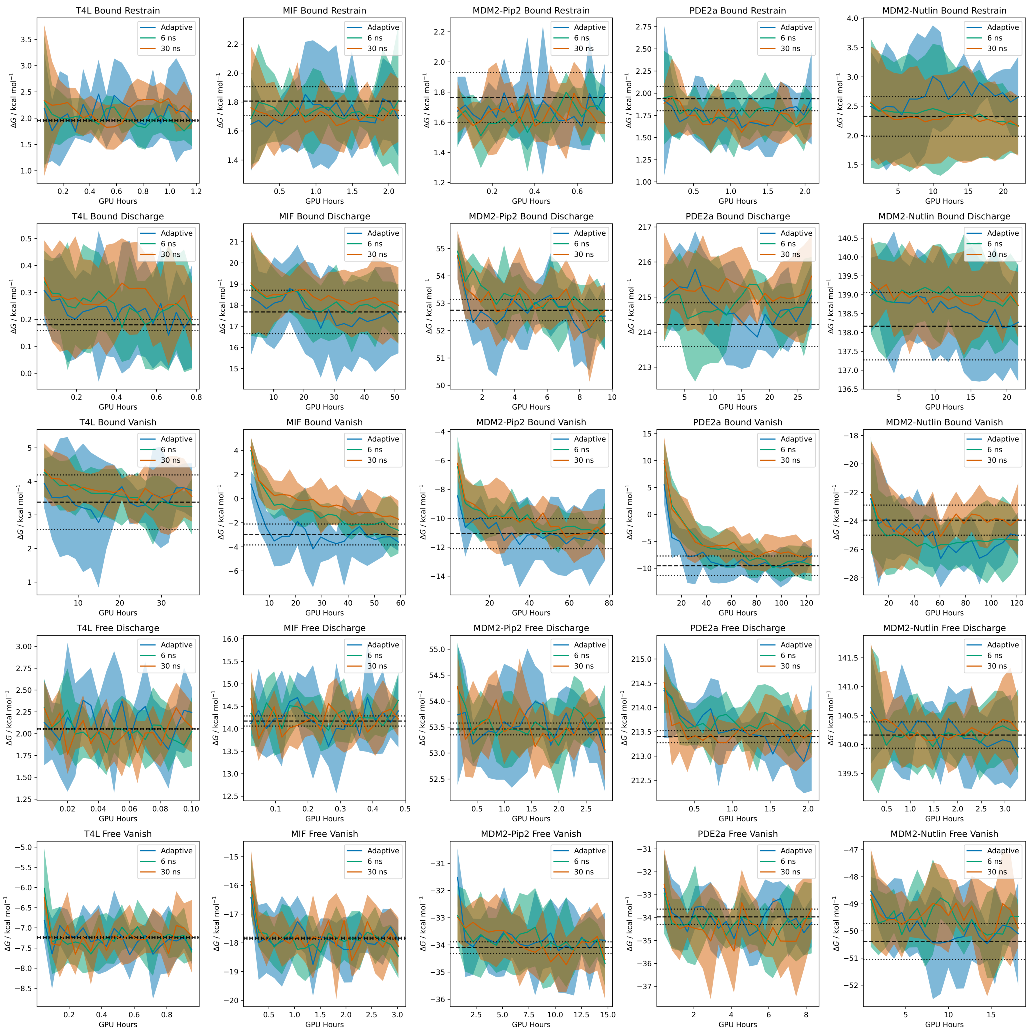

Figure S31: Estimated ΔG against sampling time for all stages of the initial test systems. Data were split into 20 equal blocks and MBAR was run on each block. "adaptive" refers to the "optimised" adaptive protocol and "6 ns" and "30 ns" refer to the respective non-adaptive protocols. The data for truncated before analysis so that the per-stage computational costs were equal to that of the cheapest stage. Shaded areas show 95 % t-based confidence intervals. The final non-adaptive ΔG is taken from the 30 ns non-adaptive result (black dotted line with 95 % CIs shown by sparser dotted lines).

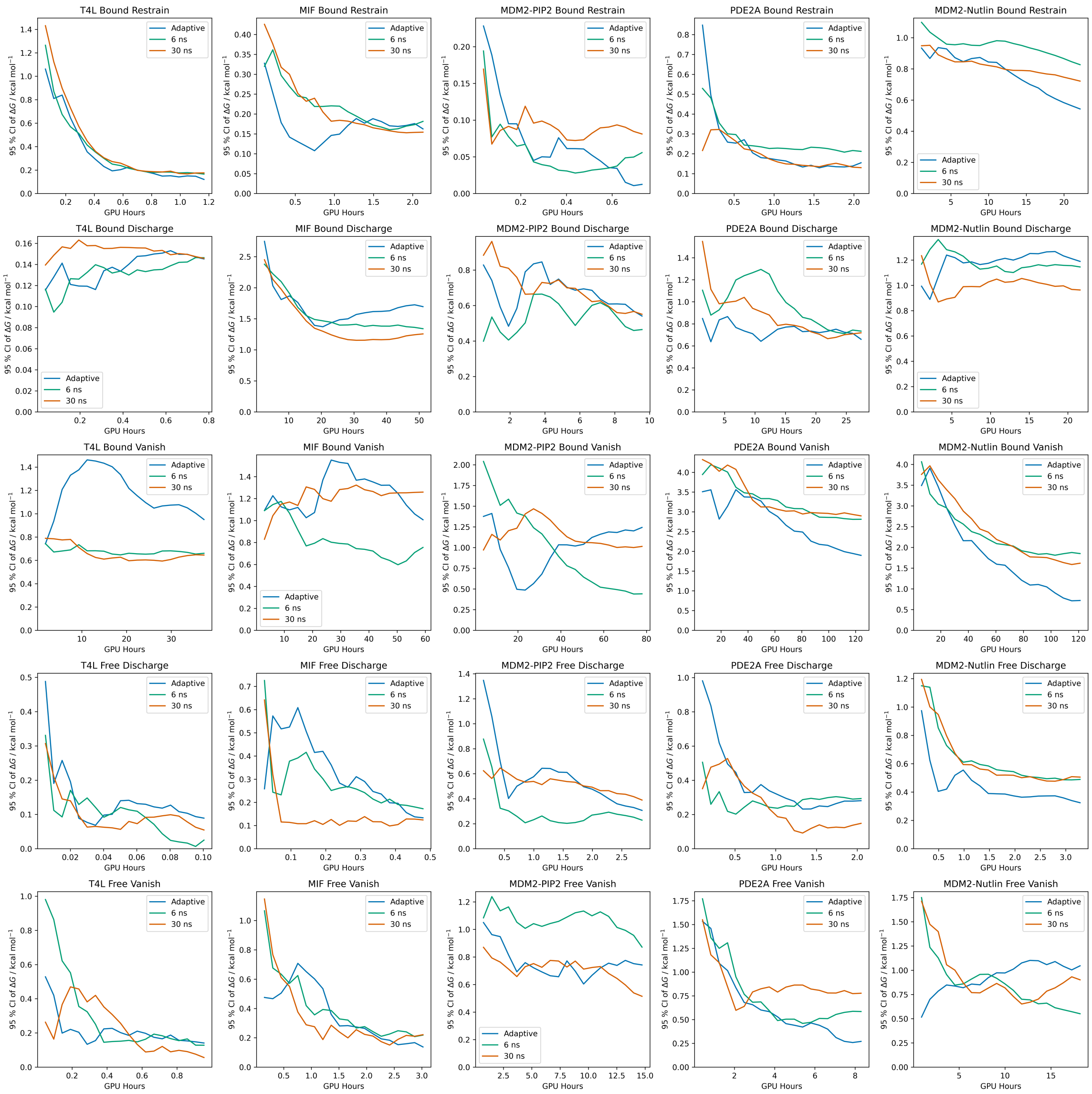

Figure S32: 95 % t-based confidence intervals of the estimated ΔG against sampling time for all stages of the initial test systems. Data were split into 20 equal blocks and MBAR was run on each block. Analysis was performed cumulatively, meaning that all prior data were included in the error calculation for each time point, and the overall error is expected to decrease with $\frac{1}{\sqrt{\text{SamplingTime}}}$. ”adaptive” refers to the “optimised” adaptive protocol and “6 ns” and “30 ns” refer to the respective non-adaptive protocols. Final portions of the data were truncated before analysis so that the per-stage computational costs were equal to that of the cheapest stage.

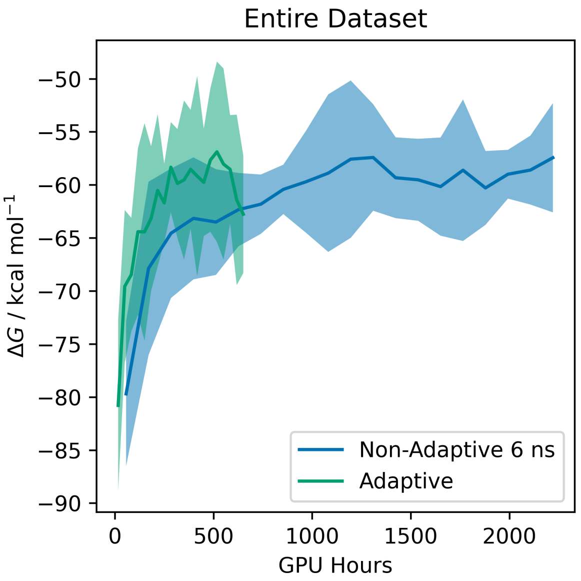

Figure S33: Estimated ΔG against sampling time for entire dataset of initial test systems. Data were split into 20 equal blocks and MBAR was run on each block. The results are shown at the centre of each block. "Adaptive" refers to the "optimised" adaptive protocol. Shaded areas show 95 % t-based confidence intervals.

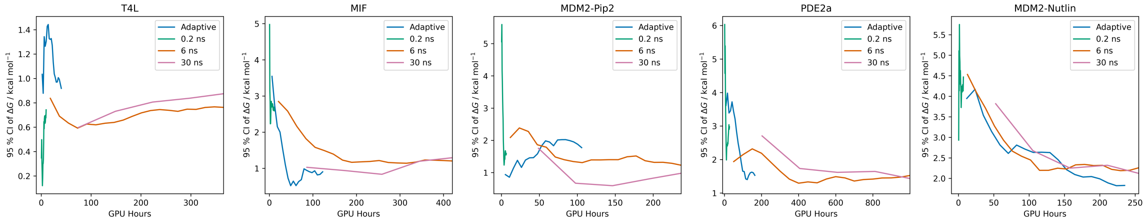

Figure S34: 95 % t-based confidence intervals of the estimated ΔG against sampling time for all stages of the initial test systems. Data were split into 20 equal blocks and MBAR was run on each block. Analysis was performed cumulatively, meaning that all prior data were included in the error calculation for each time point, and the overall error is expected to decrease with $\frac{1}{\sqrt{\text{SamplingTime}}}$. "adaptive" refers to the "optimised" adaptive protocol and "6 ns" and "30 ns" refer to the respective non-adaptive protocols. The results are shown at the centre of each block.

S21 Detailed Cyclophilin-D Results

Having excluded ligand 4, Alibay's results were re-analysed using our analysis protocol to produce Figure S35 and the corresponding metrics in Table S6.

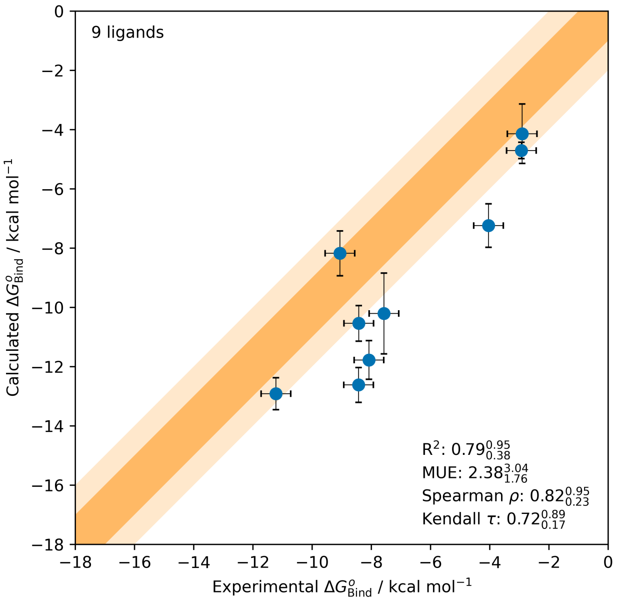

Figure S35: Experimental free energies of binding for Cyclophilin D against predicted free energies of binding as calculated by Alibay et al..[10] Experimental free energies were obtained from Grädler et al..[14] The darker and lighter shaded areas show 1 and 2 kcal mol^{-1} deviations from exact agreement, respectively. Error bars show 95 % confidence intervals, which were assumed to be 0.5 kcal mol^{-1} for experiment and calculated from the deviation between 5 replicate runs for the predictions. 95 % confidence intervals on statistics were calculated by bootstrapping with 10000 iterations of resampling.

Detailed results are summarised for our adaptive and non-adaptive runs in Table S7, while the Boresch restraint parameters are shown in Table S8. There were significant differences between the unsigned deviation from experiment and inter-replicate deviations between the results from Alibay et al. and our non-adaptive protocol (p=0.01 and 0.004, respectively, from Wilcoxon signed-rank tests). Specifically, our results showed a larger offset towards more negative free energies of binding, and larger inter-run deviations. The larger inter-run deviations may be partially explained by the much shorter run times of our non-adaptive

Table S6: Performance Metrics for Cyclophilin-D Free Energy Prediction Methods[a]

	Alibay	Non-adaptive	Adaptive	Adaptive vs Non-adaptive
r	0.89 (0.61, 0.98)	0.90 (0.68, 0.97)	0.86 (0.61, 0.95)	0.97 (0.82, 0.98)
r^2	0.79 (0.38, 0.95)	0.81 (0.46, 0.94)	0.75 (0.37, 0.91)	0.94 (0.68, 0.96)
MUE	2.38 (1.76, 3.04)	3.52 (2.61, 4.49)	2.97 (1.94, 4.15)	0.78 (0.67, 1.77)
RMSE	2.61 (1.98, 3.24)	3.84 (2.95, 4.79)	3.49 (2.31, 4.71)	1.04 (0.85, 2.19)
Spearman ρ	0.82 (0.23, 0.95)	0.77 (0.27, 0.93)	0.82 (0.28, 0.95)	0.92 (0.63, 0.97)
Kendall τ	0.72 (0.17, 0.89)	0.61 (0.17, 0.83)	0.72 (0.17, 0.83)	0.78 (0.44, 0.89)

[a] Performance metrics for the prediction of Cyclophilin-D experimental free energies using "Alibay" - the ABFE methodology of Alibay et al.;[10] "Non-adaptive" - our non-adaptive workflow; and "Adaptive" - our "optimal" adaptive workflow. "Adaptive vs Non-adaptive" shows metrics for the correlation between the adaptive and non-adaptive results. Uncertainties are given as 95 % confidence intervals, obtained by bootstrapping with 10000 iterations of resampling.

protocol (5 ns per window) compared to those used by Alibay et al. (20 ns per window). However, given the tendency of inter-run uncertainties not to decrease with increased sampling time (Section 4.1.1), combined with the larger offset to experiment, this may also suggest systematically poorer sampling in our SOMD-based protocols compared to the GROMACS-based workflow of Alibay et al.. This could be due to many factors, such as the choice of soft-core potential, or the exact definition of the decoupled state.

Finally, Figures S36 and 10 show the correlation of the non-adaptive results with experiment, and the non-adaptive results with the adaptive results, respectively.

Table S7: Detailed Breakdown of Predicted $\Delta G^{o}_{\mathrm{Bind}}$ for Cyclophilin D[a]

	Bound Restrain	Bound Discharge	Bound Vanish	Free Discharge	Free Vanish	Restraint Correction	Symmetry Correction	Exp. $\Delta G^{o}_{\mathrm{Bind}}$
2 adaptive	1.63 ± 0.05	111.38 ± 1.43	2.16 ± 1.17	106.38 ± 0.19	-12.22 ± 0.19	-10.46	0.00	-9.06 ± 0.50
2 non-adaptive	1.67 ± 0.05	111.63 ± 0.76	1.76 ± 0.70	106.26 ± 0.08	-12.20 ± 0.34	-10.46	0.00	-9.06 ± 0.50
3 adaptive	2.89 ± 2.18	12.76 ± 0.30	-0.31 ± 0.91	10.04 ± 0.09	-9.64 ± 0.22	-9.87	0.00	-2.93 ± 0.50
3 non-adaptive	1.72 ± 0.11	12.39 ± 0.52	0.80 ± 0.99	9.99 ± 0.01	-9.68 ± 0.06	-9.87	0.00	-2.93 ± 0.50
4 adaptive	1.69 ± 0.08	109.43 ± 1.28	-3.48 ± 1.58	107.30 ± 0.16	-13.98 ± 0.12	-9.39	0.00	-2.90 ± 0.50
4 non-adaptive	2.14 ± 0.77	109.98 ± 1.32	-2.52 ± 0.78	107.25 ± 0.17	-13.98 ± 0.09	-9.39	0.00	-2.90 ± 0.50
8 adaptive	1.71 ± 0.40	-54.56 ± 0.38	4.45 ± 1.07	-57.24 ± 0.24	-7.90 ± 0.18	-9.43	0.00	-4.04 ± 0.50
8 non-adaptive	1.68 ± 0.29	-54.40 ± 0.72	4.19 ± 1.02	-57.36 ± 0.15	-7.94 ± 0.10	-9.43	0.00	-4.04 ± 0.50
14 adaptive	1.72 ± 0.08	92.41 ± 0.79	1.81 ± 2.14	88.20 ± 0.23	-17.50 ± 0.35	-10.86	0.00	-11.22 ± 0.50
14 non-adaptive	1.71 ± 0.02	92.34 ± 0.62	2.46 ± 1.86	88.15 ± 0.11	-17.95 ± 0.14	-10.86	0.00	-11.22 ± 0.50
16 adaptive	1.55 ± 0.07	87.80 ± 0.37	-0.62 ± 1.66	86.61 ± 0.64	-17.35 ± 0.25	-10.19	0.00	-8.42 ± 0.50
16 non-adaptive	1.58 ± 0.03	87.95 ± 0.36	0.51 ± 2.02	86.72 ± 0.53	-17.35 ± 0.31	-10.19	0.00	-8.42 ± 0.50
27 adaptive	1.71 ± 0.07	-22.53 ± 1.14	1.52 ± 1.70	-26.52 ± 0.14	-12.84 ± 0.36	-10.92	0.00	-7.57 ± 0.50
27 non-adaptive	1.75 ± 0.05	-22.33 ± 1.27	2.59 ± 0.47	-26.74 ± 0.15	-12.96 ± 0.22	-10.92	0.00	-7.57 ± 0.50
39 adaptive	1.81 ± 0.24	73.58 ± 1.33	2.29 ± 1.31	67.45 ± 0.13	-14.22 ± 0.28	-10.46	0.00	-8.43 ± 0.50
39 non-adaptive	1.96 ± 0.70	73.54 ± 1.24	1.58 ± 1.24	67.31 ± 0.02	-14.27 ± 0.17	-10.46	0.00	-8.43 ± 0.50
40 adaptive	1.69 ± 0.25	73.82 ± 0.82	4.58 ± 0.60	67.94 ± 0.14	-12.82 ± 0.19	-10.24	0.00	-8.08 ± 0.50
40 non-adaptive	1.74 ± 0.25	73.51 ± 0.38	4.27 ± 0.78	67.78 ± 0.11	-13.02 ± 0.29	-10.24	0.00	-8.08 ± 0.50

[a] All quantities in kcal mol^{-1}. Calculation uncertainties stated as 95 % t-based confidence intervals based on the variance of 5 replicate runs, assuming Gaussian distributions. "adaptive" refers to our "optimal" adaptive protocol, and "non-adaptive" to our non-adaptive procotol. Experimental results were taken from Grädler et al., who used surface plasmon resonance.[14] The experimental uncertainties were assumed to be 0.5 kcal mol^{-1}.

Table S8: Parameters for Boresch restraints for the Cyclophilin D ligands, as labelled in Figure 3 of Clark et al..[6] K refers to a force constant and 0 denotes an equilibrium value.

	2	3	4	8	14	16	27	39	40
r1	1554	1632	1534	1535	1567	1567	1554	1558	1558
r2	1552	1630	1532	1533	1565	1565	1552	1556	1556
r3	1564	1645	1544	1545	1577	1577	1564	1568	1568
l1	13	13	11	15	20	8	4	10	7
l2	12	3	14	1	19	13	3	7	10
l3	14	6	16	4	21	25	7	12	19
r_0 / Å	5.19	6.12	4.22	4.96	4.63	6.37	3.68	5.89	5.77
θ_{A0} / Å	0.69	1.20	1.72	0.74	1.58	0.60	1.65	0.83	0.87
θ_{B0} / Rad	2.51	1.71	1.31	0.64	1.80	1.62	1.30	1.11	1.13
ϕ_{A0} / Rad	-0.24	-1.87	1.65	0.03	1.11	-0.39	1.56	0.00	-0.00
ϕ_{B0} / Rad	-0.85	0.28	-1.06	-0.93	-1.02	-0.54	-1.16	2.72	2.38
ϕ_{C0} / Rad	0.22	0.94	-2.61	-3.08	-2.51	0.15	-2.30	-3.05	3.12
k_r / kcal mol^{-1} $Å^{-2}$	19.86	9.46	8.38	17.86	18.68	16.80	21.18	18.32	19.32
$k_{\theta \mathrm{A}}$ / kcal mol^{-1} Rad^{-2}	214.50	109.66	34.20	128.18	92.20	190.46	73.02	174.74	130.04
$k_{\theta \mathrm{B}}$ / kcal mol^{-1} Rad^{-2}	103.74	34.98	30.84	61.74	128.28	110.76	64.32	81.08	90.62
$k_{\phi \mathrm{A}}$ / kcal mol^{-1} Rad^{-2}	91.72	244.78	114.02	52.02	193.68	69.42	176.98	119.12	85.94
$k_{\phi \mathrm{B}}$ / kcal mol^{-1} Rad^{-2}	25.70	102.60	69.22	13.84	144.82	33.40	216.74	77.78	91.02
$k_{\phi \mathrm{C}}$ / kcal mol^{-1} Rad^{-2}	33.22	60.16	37.46	10.04	90.32	84.82	70.06	73.10	47.98

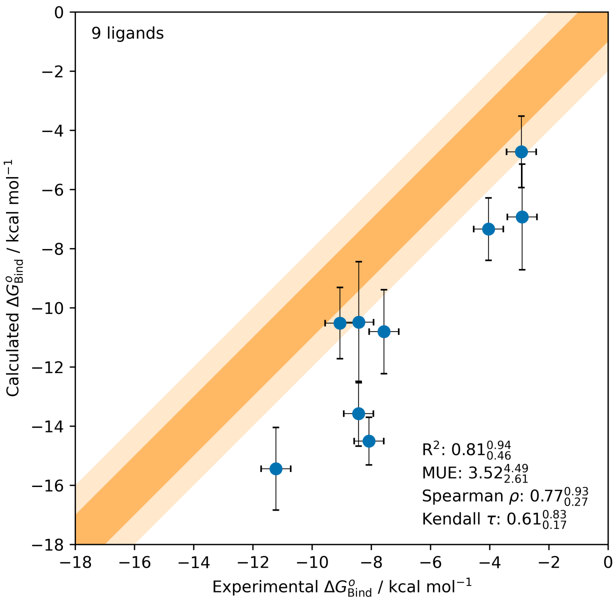

Figure S36: Experimental free energies of binding for Cyclophilin D against predicted free energies of binding obtained using the non-adaptive protocol. Experimental free energies were obtained from Grädler et al..[14] The darker and lighter shaded areas show 1 and 2 kcal mol^{-1} deviations from exact agreement, respectively. Error bars show 95 % confidence intervals, which were assumed to be 0.5 kcal mol^{-1} for experiment and calculated from the deviation between 5 replicate runs for the predictions. 95 % confidence intervals on statistics were calculated by bootstrapping with 10000 iterations of resampling.

S22 Details of Cyclophilin-D Adaptive Runs

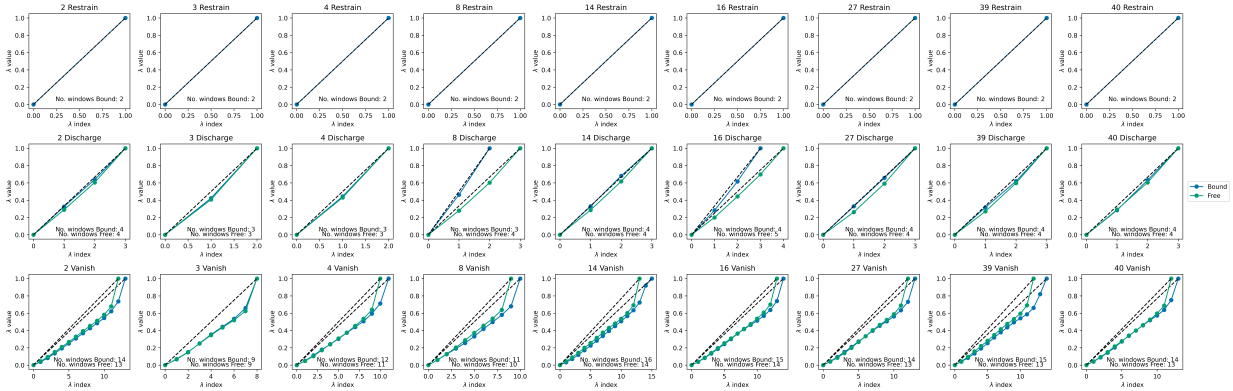

Figure S37: λ values selected for each of the Cyclophilin D ligands using a thermodynamic speed of 2 kcal mol^{-1}.

Table S9: Allocation of Sampling Time Between Cyclophilin D Ligands using Adaptive and Non-Adaptive Protocols[a]

	adaptive	non-adaptive
Ligand 2	104	161
Ligand 3	146	137
Ligand 4	216	154
Ligand 8	65	174
Ligand 14	124	164
Ligand 16	77	189
Ligand 27	123	180
Ligand 39	161	170
Ligand 40	83	177
Total	1098	1505

[a] All times given in GPU-hours.

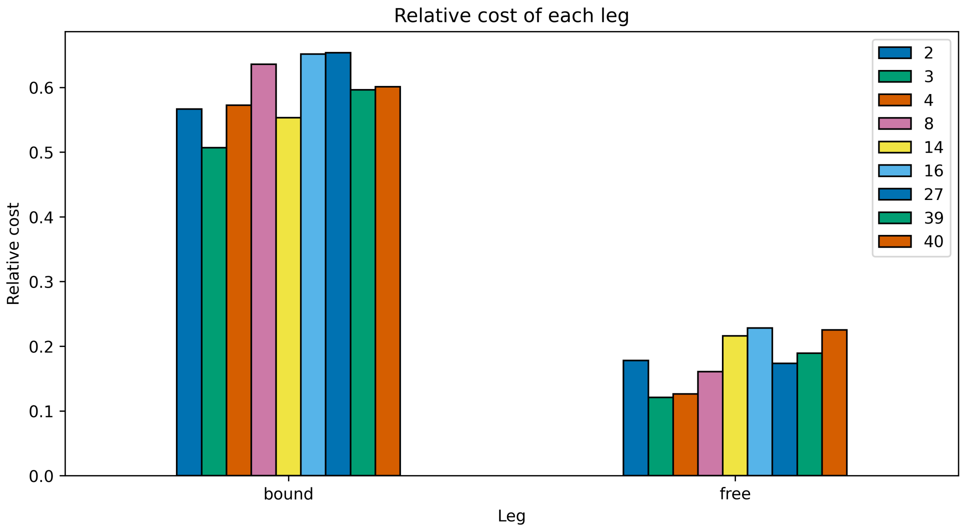

Figure S38: Relative computational costs of each leg for the Cyclophilin D systems, calculated relative to the MIF180 bound leg. Calculated based on the average time taken to run a ns of simulation on a single GPU. The absolute cost of the MIF180 bound leg was 0.21 GPU hours / ns. All computational costs were assessed running on NVIDIA GeForce RTX2080 SUPER GPUs.

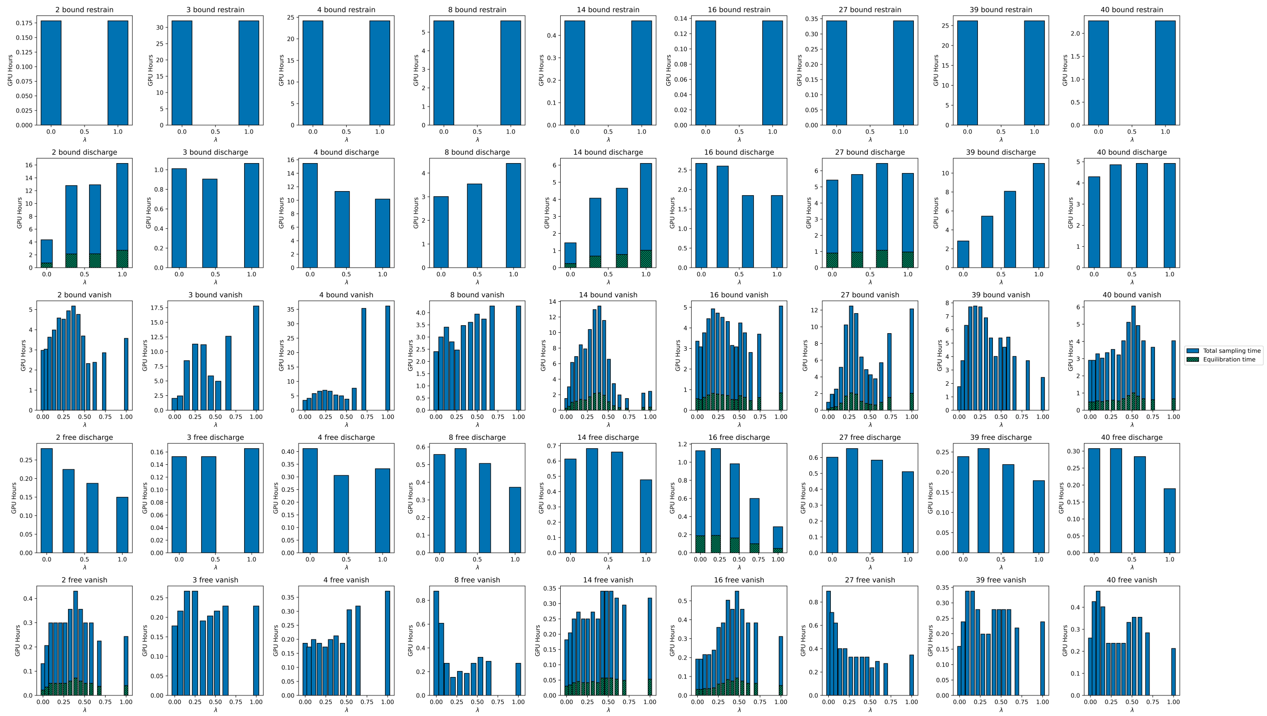

Figure S39: Per-window breakdown of sampling times allocated to the Cyclophilin D systems with the "Optimised" adaptive protocol.

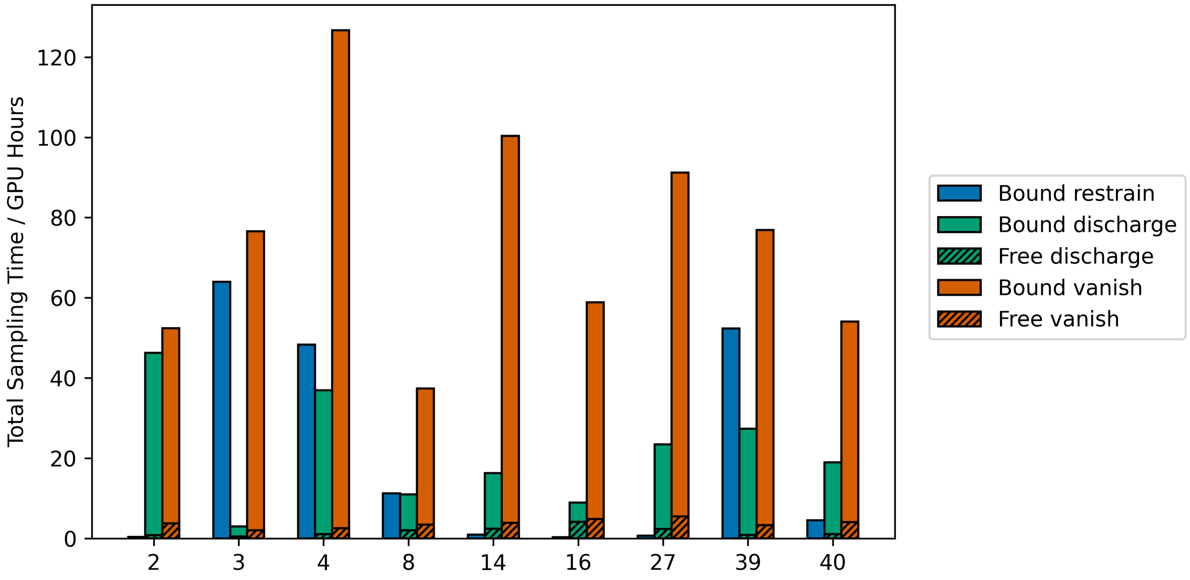

Figure S40: Per-stage breakdown of sampling times allocated to the Cyclophilin D systems with the "Optimised" adaptive protocol.

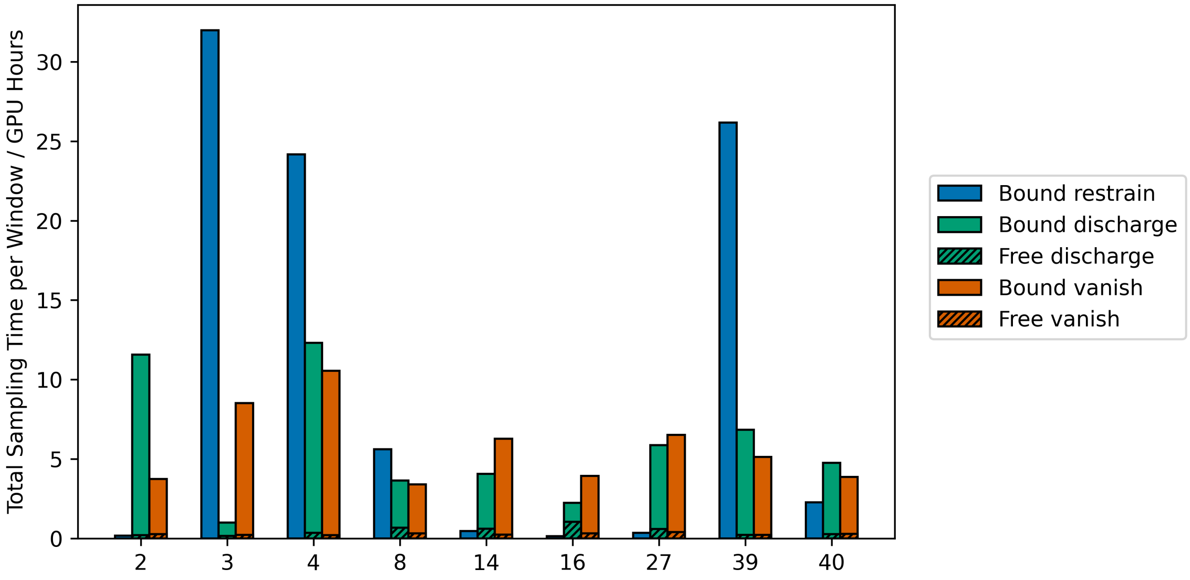

Figure S41: Per-stage breakdown of the per-window sampling times allocated to the Cyclophilin D systems with the "Optimised" adaptive protocol.

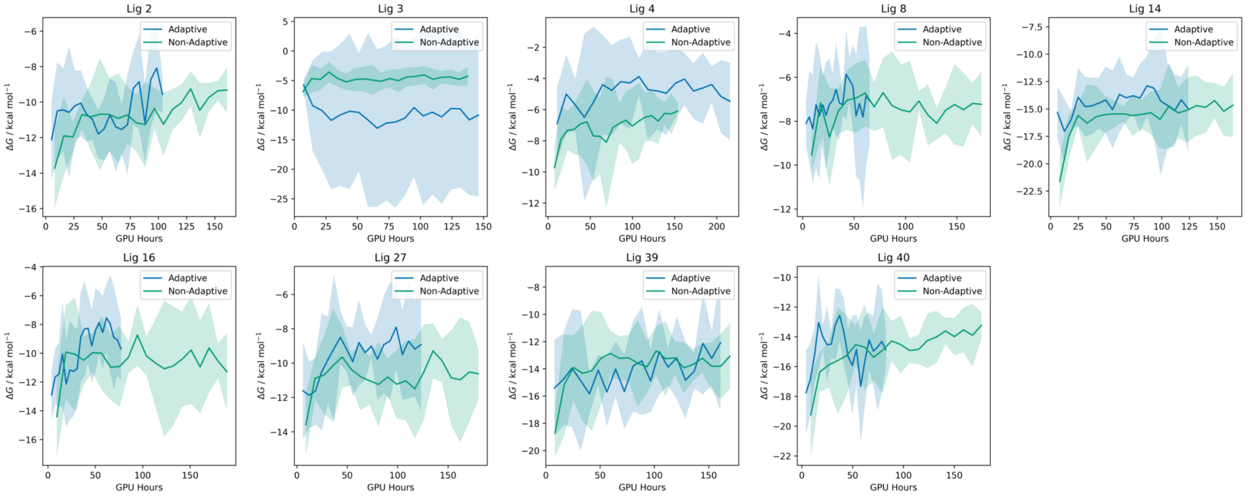

Figure S42: Estimated ΔG against sampling time for the Cyclophilin D ligands. All data were split into 20 equal blocks and MBAR was run on each block. Shaded areas show 95 % t-based confidence intervals. The apparent large deviation for ligand 3 adaptive runs occurred due to this weak binder flipping in the binding site during the $\lambda = 0$ restrain-stage window for some runs. This could equally have occurred during the non-adaptive runs. Despite the large deviation shown above, running MBAR on all the data produced a reasonable result much closer to the expected ≈ 1.23 kcal mol^{-1}, as shown in Table S7.

References

(1) Nguyen, T. H.; Minh, D. D. L. Intermediate Thermodynamic States Contribute Equally to Free Energy Convergence: A Demonstration with Replica Exchange. *J. Chem. Theory Comput.* **2016**, *12*, 2154–2161.

(2) Bennett, C. H. Efficient Estimation of Free Energy Differences from Monte Carlo Data. *J. Comput. Phys.* **1976**, *22*, 245–268.

(3) Shirts, M. R. Reweighting from the mixture distribution as a better way to describe the multistate Bennett acceptance ratio. *arXiv preprint arXiv:1704.00891* **2017**,

(4) Blondel, A. Ensemble variance in free energy calculations by thermodynamic integration: Theory, optimal "Alchemical" path, and practical solutions. *J. Comput. Chem.* **2004**, *25*, 985–993.

(5) Merski, M.; Fischer, M.; Balius, T. E.; Eidam, O.; Shoichet, B. K. Homologous Ligands Accommodated by Discrete Conformations of a Buried Cavity. *Proc. Natl. Acad. Sci. U.S.A.* **2015**, *112*, 5039–5044.

(6) Clark, F.; Robb, G.; Cole, D. J.; Michel, J. Comparison of Receptor–Ligand Restraint Schemes for Alchemical Absolute Binding Free Energy Calculations. *J. Chem. Theory Comput.* **2023**, *19*, 3686–3704.

(7) Mendoza-Martinez, C.; Papadourakis, M.; Llabrés, S.; Gupta, A. A.; Barlow, P. N.; Michel, J. Energetics of a Protein Disorder–Order Transition in Small Molecule Recognition. *Chem. Sci.* **2022**, *13*, 5220–5229.

(8) Michelsen, K.; Jordan, J. B.; Lewis, J.; Long, A. M.; Yang, E.; Rew, Y.; Zhou, J.; Yakowec, P.; Schnier, P. D.; Huang, X.; Poppe, L. Ordering of the N-Terminus of Human MDM2 by Small Molecule Inhibitors. *J. Am. Chem. Soc.* **2012**, *134*, 17059–17067.

(9) Huggins, D. J. Comparing the Performance of Different AMBER Protein Forcefields, Partial Charge Assignments, and Water Models for Absolute Binding Free Energy Calculations. *J. Chem. Theory Comput.* **2022**, *18*, 2616–2630.

(10) Alibay, I.; Magarkar, A.; Seeliger, D.; Biggin, P. C. Evaluating the Use of Absolute Binding Free Energy in the Fragment Optimisation Process. *Commun. Chem.* **2022**, *5*, 105.

(11) Alibay, I.; Magarkar, A.; Seeliger, D.; Philip, B. C. Sampled $\Delta H/\Delta\lambda$ and ΔH Data from ABFE Calculations of 10 Ligands Bound to Cyclophilin D. 2022.

(12) Kumar, R.; Carroll, C.; Hartikainen, A.; Martin, O. ArviZ a Unified Library for Exploratory Analysis of Bayesian Models in Python. *J. Open Source Softw.* **2019**, *4*, 1143.

(13) Chodera, J. D. A Simple Method for Automated Equilibration Detection in Molecular Simulations. *J. Chem. Theory Comput.* **2016**, *12*, 1799–1805.

(14) Grädler, U.; Schwarz, D.; Blaesse, M.; Leuthner, B.; Johnson, T. L.; Bernard, F.; Jiang, X.; Marx, A.; Gilardone, M.; Lemoine, H.; Roche, D.; Jorand-Lebrun, C. Discovery of Novel Cyclophilin D Inhibitors Starting from Three Dimensional Fragments with Millimolar Potencies. *Bioorg. Med. Chem. Lett.* **2019**, *29*, 126717.
